# Biological and target synthetic treatments for chronic spontaneous urticaria: A systematic review and network meta‐analysis

**DOI:** 10.1002/clt2.70052

**Published:** 2025-05-06

**Authors:** Zuotao Zhao, Yaqi Zheng, Xiaoting Song, Chengyue Peng, Shuanglu Liao, Peixin Zhang, Yen Tan, Xiaojie Huang, Litao Zhang

**Affiliations:** ^1^ Department of Dermatology Tianjin Academy of Traditional Chinese Medicine Affiliated Hospital Tianjin China; ^2^ Tianjin Institute of Integrative Dermatology Tianjin China; ^3^ Department of Dermatology and Venerology Peking University First Hospital Beijing China; ^4^ Beijing Key Laboratory of Molecular Diagnosis on Dermatoses Beijing China; ^5^ National Clinical Research Center for Skin and Immune Diseases Beijing China; ^6^ NMPA Key Laboratory for Quality Control and Evaluation of Cosmetics Beijing China; ^7^ Peking University School of Nursing Beijing China; ^8^ Clinical and Research Center for Infectious Diseases Beijing China; ^9^ Beijing Youan Hospital Capital Medical University Beijing China

**Keywords:** biologics, BTK inhibitor, chronic spontaneous urticaria, network meta analysis, synthetic target‐specific drugs, treatment comparison

## Abstract

**Background:**

Most biological and synthetic target‐specific drugs for antihistamine‐refractory chronic spontaneous urticaria (CSU) have not been compared head‐to‐head. This systematic review and network meta‐analysis evaluated their relative efficacy and safety.

**Methods:**

Searches were conducted on PubMed, Embase, Web of Science and Cochrane library databases to March 25, 2024 for randomized trials. A random‐effects model was used to calculate the network estimates reported as mean differences (MD) and odds ratios (OR) with corresponding 95% CIs. Main outcomes included the weekly urticaria activity score (UAS7), adverse events (AEs) and serious adverse events (SAEs).

**Results:**

23 randomized clinical trials with 6933 participants that compared 26 different interventions or dosages and placebos were included. Omalizumab 300 mg [MD −10.07, 95% CI (−11.35; −8.82)] continues to demonstrate superior efficacy compared with placebo. Remibrutinib, at doses of 35 mg once daily [MD −7.80, 95% CI (−12.76; −2.51)], 25 mg twice daily [MD −7.69, 95% CI (−9.85; −5.76)], and 10 mg twice daily [MD −7.61, 95% CI (−12.59; −2.47)], had the best overall performance for efficacy and safety. Dupilumab, fenebrutinib, and rilzabrutinib also showed greater efficacy than placebo. The results were similar for the proportion of patients who achieved UAS7 ≤ 6 or UAS7 = 0. No significant differences were found among all treatment comparisons for AEs and SAEs.

**Conclusion:**

The findings of this study indicate that the biological agent omalizumab 300 mg and the oral small molecule remibrutinib at doses of 35 mg, 25 mg, or 10 mg are recommended for patients with antihistamine‐refractory CSU.

**Trial Registration:**

PROSPERO: CRD42024516289

## 1 INTRODUCTION

Chronic spontaneous urticaria (CSU) is a mast cell‐driven disease, featured by the recurrence of hives (wheals), angioedema, or both for at least 6 weeks, with or without known causes.^1^ The point prevalence of CSU has been reported to range from 0.1% to 3.4% across different studies, with a higher incidence observed in females and individuals over the age of 20.^2^, ^3^, ^4^ This condition significantly impairs patients' physical, emotional, and social well‐being.^5^

A stepwise treatment algorithm is the cornerstone of CSU management, including second‐generation H1‐antihistamines (H1‐AH), omalizumab and cyclosporine.^6^ However, many patients remain refractory to these therapies.^7^ Standard and even up‐dosing of H1‐AH often fail to provide adequate relief for many CSU patients,^8^ leading to a considerable socio‐economic burden of H1‐AH refractory CSU.^9^ Many randomized clinical trials (RCTs) and real‐world studies have evaluated the efficacy of omalizumab in patients with CSU, demonstrating favorable outcomes.^7^, ^10^, ^11^, ^12^, ^13^, ^14^, ^15^, ^16^, ^17^, ^18^, ^19^, ^20^ A previous meta‐analysis including seven trials reported that patients treated with omalizumab had significantly reduced itch and wheal scores compared to placebo, with the greatest improvement in weekly itch scores observed with a 300 mg dose administered every 4 weeks.^21^ The response to omalizumab therapy depends on the patient's disease endotype, and up‐dosing or shortening the interval may benefit in refractory cases.^22^ Nevertheless, despite omalizumab's favorable efficacy and widespread use, with studies showing that up to 66% of patients achieve well‐controlled disease, 34%–48% still demonstrate a partial or no response,^14^, ^17^, ^18^, ^20^ highlighting the need for novel therapeutic options to improve CSU management. In some cases, other available treatments such as phototherapy, methotrexate, mycophenolate mofetil, dapsone, dupilumab, and tumor necrosis factor alpha (TNF‐a) inhibitors have been employed with varying degrees of efficacy.^23^ Furthermore, the emergence of more biologics and small molecule drugs is promising, of which is currently being studied in randomized clinical trials.^24^, ^25^, ^26^, ^27^, ^28^, ^29^, ^30^, ^31^ To date, approved or investigational biological and targeted synthetic therapies for CSU include anti‐IgE monoclonal antibodies (omalizumab, ligelizumab, UB221), Bruton tyrosine kinase (BTK) inhibitors (fenebrutinib, remibrutinib, rilzabrutinib), anti‐IL‐5 monoclonal antibodies (mepolizumab, reslizumab, benralizumab), anti‐thymic stromal lymphopoietin (TSLP) monoclonal antibody (tezepelumab), anti‐Siglec‐8 monoclonal antibody (lirentelimab), CRTH2 antagonist (AZD1981), and anti‐KIT monoclonal antibody (barzolvolimab). The increasing availability of mechanistic treatment options holds significant promise for both healthcare providers and patients, potentially improving individualized care and treatment outcomes in CSU.

Significant knowledge gaps remain regarding the efficacy and safety of various biologics and synthetic target‐specific drugs for the treatment of CSU. Previous network meta‐analyses (NMA) revealed that the biological agents ligelizumab 72 or 240 mg and omalizumab 300 or 600 mg are effective treatment options for CSU^32^, ^33^; however, ligelizumab did not demonstrate superiority over omalizumab in Phase III trials and was subsequently discontinued by Novartis.^34^ In addition, with the development of novel therapeutic agents and additional randomized controlled trials (RCTs), an updated NMA is needed Therefore, we performed a comprehensive systematic review and NMA of randomized clinical trials that investigated pharmacologic treatments for H1‐AH refractory CSU, aiming to provide evidence‐based insights for clinical decision‐making.

## 2 METHODS

This systematic review and NMA was performed using a prespecified protocol for literature search and synthesis as recommended by the statement “Preferred Reporting Items for Systematic Reviews and Meta‐Analyses” (PRISMA).^35^ The prespecified protocol was registered on PROSPERO (CRD42024516289).

### 2.1 Search strategy and selection criteria

We searched PubMed, Embase, Web of Science and Cochrane library databases to March 25, 2024, for randomized trials addressing biologics and small‐molecule drugs for CSU, with no language restrictions. The ClinicalTrials.gov search functions enable us to search for new and updated entries. The full search strategy can be found in Supporting Information S1: eTable 1. For published trials, we will conduct a comprehensive search of trial registries to obtain additional results. Furthermore, we will thoroughly review the reference lists of eligible studies and actively contact pharmaceutical companies in the field to acquire potentially relevant studies and data.

### 2.2 Eligibility criteria

We included randomized clinical trials involving populations of all ages with H1‐AH refractory CSU. Interventions of interest include biologics (omalizumab, ligelizumab, quilizumab, dupilumab, benralizumab, barzolvolimab, lirentelimab, tezepelumab, mepolizumab, tezepelumab, AZD 1981) and small‐molecule drugs (remibrutinib, fenebrutinib, rilzabrutinib). Comparators may be an active comparator, as mentioned above, an antihistamine agent or placebo. In alignment with the 2021 joint guidelines from the European Academy of Allergology and Clinical Immunology (EAACI), the Global Allergy and Asthma European Network (GA^2^ LEN), the European Dermatology Forum, and the World Allergy Organization, we adopted the weekly urticaria activity score (UAS7) as the main metric for assessing urticaria symptom.^6^ The primary outcomes included change from baseline in UAS7, the proportion of patients achieving complete response (UAS7 = 0), and those with well‐controlled disease (UAS7 ≤ 6), occurrence of adverse events (AEs) and serious adverse events (SAEs). The secondary outcome included change from baseline in weekly itch severity score (ISS7), weekly hive severity score (HSS7), and dermatology life quality index (DLQI). Studies with inaccessible full‐text or insufficient data for data pooling and analysis, meeting abstracts, trials with trial registration entry but early termination, and comments will be excluded.

### 2.3 Screening and abstraction process

Two reviewers (Y.Z and P.Z) independently screened all related articles to determine which studies should be included or excluded according to the prespecified eligibility criteria. The reasons for the exclusion of any study will be listed.

Two reviewers (Y.Z and C.P) independently extracted data from the included studies using a predefined data extraction form. The extracted information included the first author, year of publication, region where the study was conducted, study design, sample size, intervention, treatment duration, age and sex of participants, baseline symptom or severity score reported by patients, and duration of CSU.^32^ For continuous outcomes, the difference in mean change from baseline and the standard deviation were extracted for different treatments and placebo. If the median of the baseline change was available but not the mean of the baseline change, the median was used instead of the mean because we assumed the distribution to be symmetric.^36^, ^37^ If no baseline change value was provided, the difference was calculated using the baseline and follow‐up values. For dichotomous results, the sample size and number of affected patients were extracted. If results were only available in figures without the exact values provided, we used WebPlotDigitizer to estimate the values.^38^ Two reviewers independently derived these estimates, and we used the mean of their estimates. Any discrepancies were resolved through consultation with a third reviewer.

### 2.4 Data analysis

We performed random‐effects NMA for each outcome within a Bayesian framework using R statistical software (gemtc package).^39^, ^40^ A network graph was generated to visualize the overall structure of the network. A forest plot was created to display the effect size of each treatment relative to the placebo. The summary results will be presented as odds ratio (OR) or mean difference (MD) with 95% confidence interval (95% CI). To rank the treatment hierarchy in the NMA, the surface under the cumulative ranking curve (SUCRA) was calculated for each treatment comparison.^41^, ^42^

### 2.5 Subgroup and sensitivity analyzes

Subgroup analyses were planned for patients of different ages and for patients receiving short‐term treatment (≤16 weeks) and long‐term treatment (>16 weeks). Treatment of more than 16 weeks was defined as long‐term treatment according to the NMA by Drucker et al.^39^ and Kendziora et al.^33^ Further sensitivity analyses may be conducted for trials with a high risk of bias.

### 2.6 Risk of bias and certainty of evidence

The methodological quality of each randomized controlled trial included in this study was evaluated by the Cochrane Risk of Bias Tool 2.^43^ The tools assessed the domain‐specific quality in 7 aspects as follows: random sequence generation, allocation concealment, blinding of participants and personnel, blinded outcome assessment, incomplete outcome data and selective reporting. Two reviewers (Y.Z and P.Z) independently evaluated the risk of bias in seven domains and overall as “high”, “low”, or “some concerns.” Disagreements will be resolved by discussion. If an agreement may not be reached, a third reviewer will be consulted. Heterogeneity was assessed by the *I* ^*2*^ statistic for each comparison as well as for the overall network.^44^ For all outcomes, the consistency of the network meta‐analysis models will be evaluated by the node‐splitting approach.^45^, ^46^ Based on the GRADE framework,^47^ the quality of evidence was independently graded by Confidence In Network Meta‐Analysis (CINeMA), considering six domains including within‐study bias, cross‐study bias, indirectness, imprecision, heterogeneity, and inconsistency. Certainty of evidence was categorized as very low, low, medium, and high quality.^32^, ^48^

## 3 RESULTS

After title, abstract, and full text review, 23 trials were included in the NMA.^7^, ^12^, ^13^, ^14^, ^15^, ^16^, ^17^, ^18^, ^19^, ^20^, ^24^, ^25^, ^26^, ^27^, ^28^, ^29^, ^30^, ^31^, ^34^, ^49^, ^50^, ^51^, ^52^ The detailed process of study selection is demonstrated in Supporting Information S1: eFigure 1. Based on the overall risk‐of‐bias assessment, 15 trials had a low risk of bias,^12^, ^13^, ^14^, ^15^, ^16^, ^17^, ^18^, ^20^, ^24^, ^28^, ^29^, ^34^, ^49^, ^50^, ^52^ 8 trials had some concerns,^7^, ^19^, ^25^, ^26^, ^27^, ^30^, ^31^, ^51^ and no trial had a high risk of bias (Supporting Information S1: eFigure 2). These trials investigated 25 different interventions or dosages and 1 placebo. The included studies differed regarding clinical study stage, primary and secondary endpoints, and inclusion of only adult,^7^, ^12^, ^13^, ^16^, ^19^, ^24^, ^25^, ^26^, ^27^, ^28^, ^29^, ^30^, ^31^, ^34^, ^49^, ^50^, ^51^ only adolescents^52^ or both adults and adolescents.^14^, ^15^, ^17^, ^18^, ^20^ In total, 6933 participants with CSU were identified, with mean age ranging from 15.0 to 47.1 years, proportion of females ranging from 40% to 90%, baseline UAS7 ranging from 11 to 32.2, and disease duration ranging from 1.6 to 5.2 years. The main characteristics of all 23 included studies and participants are listed in Table 1.

**TABLE 1:** Design and characteristics of the population included in the NMA.

Author, year	Phase	Study design (sample size)	Treatment duration (wks)	Intervention	Age, mean ± SD, y		Females, No. (%)		Baseline UAS7, mean ± SD		Duration of CSU, mean ± SD, y		Outcome	Overall risk of bias
					Intervention	Control	Intervention	Control	Intervention	Control	Intervention	Control		
Altrichter et al., 2024	2b	RCT, DB (*N* = 155)	24	Benralizumab 30 or 60 mg q4w	46.2 ± 14.7	47.1 ± 14.3	89 (77.4)	25 (62.5)	32.0 ± 7.5	31.6 ± 7.2	5.2 ± 7.0	4.4 ± 7.8	ISS7, UAS7, HSS7, UCT, DLQI, CU‐Q2oL, AEs	Some concerns
Saini S et al., 2023	3	RCT, DB (*N* = 925)	24	Remibrutinib 25 mg bid	43.3 ± 14.4	43.6 ± 11.1	409 (66.7)	209(70.0)	30.5 ± 7.9	29.6 ± 7.6	6.2 ± 8.5	5.4 ± 6.8	UAS7, ISS7, HSS7, AEs	Low
Maurer et al., 2022	2b	RCT, DB (*N* = 311)	12	Remibrutinib 10, 35 or 100 mg qd; remibrutinib10, 25 or 100 mg bid	45.0 ± 14.9	45.1 ± 15.2	197 (73.5)	25 (58.1)	30.4 ± 7.1	27.6 ± 7.6	5.1 ± 6.4	3.6 ± 4.8	UAS7, DLQI	Low
Staubach et al., 2022	2b	RCT, DB (*N* = 49)	24	Ligelizumab 24 or 120 mg q4w	15.0 ± 1.7	14.4 ± 1.5	19 (51.3)	9 (75.0)	30 ± 7.4	32.5 ± 9.0	2.6 ± 2.4	4.1 ± 4.1	UAS7, ISS7, HSS7, DLQI, AEs	Low
Maurer et al., 2019	2	RCT, DB (*N* = 340)	20	Ligelizumab 24, 72 or 240 mg q4w; omalizumab 300 mg q4w	Ligelizumab, 43.8 ± 12.1; omalizumab, 41.8 ± 13.1	45.4 ± 11.2	Ligelizumab, 159 (75.0); omalizumab, 66 (77.6)	31 (72.1)	Ligelizumab, 30.5±7.3; omalizumab, 29.3 ± 7.9	31.1 ± 6.8	Ligelizumab, 3.8 ± 5.2; omalizumab, 5.1 ± 7.5	3.6 ± 3.5	HSS7, ISS7, UAS7, AEs	Low
Maurer et al., 2023	3	RCT, DB (*N* = 2057)	52	Ligelizumab, 72 or 120 mg q4w; omalizumab 300 mg q4w	Ligelizumab, 42.6 ± 13.5; omalizumab, 43.2 ± 13.7	43.1 ± 13.5	Ligelizumab, 891 (72.4); omalizumab, 443 (71.7)	156 (74.6)	Ligelizumab, 29.7 ± 7.6; omalizumab, 29.8 ± 7.7	31.0 ± 7.0	NR	NR	ISS7, UAS7, AEs	Low
Giminez et al., 2022	2b	RCT, DB (*N* = 382)	20	Ligelizumab 72 or 240 mg q4w; omalizumab 300 mg q4w	Ligelizumab, 43.8 ± 12.1; omalizumab, 41.8 ± 13.1	45.4 ± 11.2	Ligelizumab, ; omalizumab, 66 (77.6)	31 (72)	Ligelizumab, 30.5 ± 7.3; omalizumab, 29.3 ± 7.9	31.1 ± 6.8	Ligelizumab, 3.8 ± 5.2; omalizumab, 5.1 ± 7.5	3.6 ± 3.5	DLQI	Low
Maurer et al., 2024	3	RCT, DB (*N* = 108)	24	Dupilumab 300NR200 mg q2w	44.1 ± 16.4	44.1 ± 15.6	78 (62.9)	91 (74.6)	31.5 ± 7.5	31.3 ± 8.1	6.9 ± 8.7	7.6 ± 9.0	HSS7, ISS7, UAS7, AEs	Low
Harris et al., 2016	NR	RCT, DB (*N* = 32)	20	Quilizumab 450 mg q4w	44.7 ± 13.2	41.8 ± 12.3	10 (67)	13 (77)	28.7 ± 7.1	29.3 ± 7.2	8.3 (13.0)	14.2 ± 19.0	ISS7, UAS7, HSS7	Some concerns
Oliver et al., 2019	2	RCT, DB (*N* = 28)	4	AZD1981 40 mg tid	41.8 ± 12.3	45.2 ± 12.5	10 (71.4)	10 (83.3)	20.6 ± NR	22.8 ± NR	NR	NR	UAS7, ISS7, DLQI, AEs	Some concerns
Metz et al., 2021	2	RCT, DB (*N* = 93)	8	Fenebrutinib 50 or 150 mg qd; fenebrutinib 200 mg bid	44.2 ± 14.2	40.2 ± 14.7	54 (77.1)	17 (74)	28.1 ± 7.9	25.5 ± 4.7	FEN50mg: 2.1 (range, 0–40.0); FEN150mg: 2.6 (range, 0.5–36.6); FEN200mg: 3.9 (range, 0.6–45.3)	1.2 (range, 0–14.4)	ISS7, UAS7, HSS7, AEs	Some concerns
Hide et al., 2017	3	RCT, DB (*N* = 218)	12	Omalizumab 150 or 300 mg q4w	43.6 ± 12.2	42.5 ± 14.3	83 (57.6)	48 (64.9)	30.7 ± 7.3	30.1 ± 6.5	4.0 ± 4.7	4.7 ± 6.2	ISS7, UAS7, HSS7, DLQI, AEs	Low
Saini et al., 2011	2	RCT, DB (*N* = 90)	4	Omalizumab 75, 300 or 600 mg q4w	40.6 ± 14.2	41.2 ± 16.2	44 (63.8)	17(81.0)	27.3 ± 7.4	31.0 ± 7.3	NR	NR	ISS7, UAS7, HSS7, AEs	Low
Saini et al., 2015	3	RCT, DB (*N* = 319)	24	Omalizumab 75, 150 or 300 mg q4w	41.4 ± 14.1	40.4 ± 15.6	179 (75.2)	52 (65.0)	31.1 ± 6.6	31.1 ± 6.7	6.9 ± 9.0	7.0 ± 9.7	ISS7, UAS7, HSS7, DLQI, CU‐Q2oL, AEs	Low
Kaplan et al., 2013	3	RCT, DB (*N* = 335)	24	Omalizumab 300 mg q4w	42.7 ± 13.9	44.3 ± 14.7	186 (73.8)	55 (66.3)	31.2 ± 6.6	30.2 ± 6.7	7.0 ± 8.8	8.8 ± 11.2	ISS7, UAS7, HSS7, DLQI, CU‐Q2oL, AEs	Low
Staubach et al., 2016	3	RCT, DB (*N* = 91)	28	Omalizumab 300 mg q4w	44.9 (range, 20–73)	41.4 (range, 20–61)	30 (68.2)	33 (70.2)	26.5 ± 8.2	27.9 ± 8.7	8.4 ± 9.3	7.4 ± 8.8	CU‐Q2oL, DLQI, UAS7, AEs	Low
Jörg et al., 2018	NR	RCT, DB (*N* = 30)	16	Omalizumab 300 mg q4w	41.8 ± 15.2	42.4 ± 13.3	8 (40.0)	8 (80.0)	11.0 (IQR, 2.5; 21.5)	18.5 (IQR, 11.3; 23.5)	1.6 (IQR, 1.0; 2.8)	2.25 (IQR, 1.7; 5.4)	UAS7	Some concerns
Maurer et al., 2013	3	RCT, DB (*N* = 323)	12	Omalizumab 75, 150 or 300 mg q4w	42.3 ± 14.0	43.1 ± 12.5	189 (77.8)	55 (69.6)	30.6 ± 6.9	31.0 ± 6.6	6.2 ± 7.8	7.2 ± 10.7	ISS7, UAS7, HSS7, DLQI, AEs	Low
Metz et al., 2017	2	RCT, DB (*N* = 30)	12	Omalizumab 300 mg q4w	37.5 ± 11.0	41.1 ± 8.0	18 (90.0)	8 (80.0)	32.2 ± 8.0	31.6 ± 7.7	NR	NR	UAS7; ISS7, DLQI	Low
Yuan et al., 2022	3	RCT, DB (*N* = 418)	20	Omalizumab 300 or 150 mg q4w	OMA300: 38.0 (range, 20–67); OMA150: 36.0 (range, 18–67)	42.0 (range, 22–72)	223 (66.6)	53 (63.9)	31.2 ± 7.3	33.96 ± 6.81	4.1 ± 5.7	4.79 ± 6.46	ISS7, UAS7, DLQI, AEs	Low
Sussman et al., 2020	3b	Randomized, OL (*N* = 314)	24	Omalizumab 300 mg q4w	45.8 ± 13.6	NR	99 (72.8)	NR	30.0 ± 7.5	NR	NR	NR	UAS7, AEs	Some concerns
Maurer et al., 2024	2b	RCT; TB (*N* = 125)	16	Tezepelumab 210 mg q4w; tezepelumab 420 mg q2w; omalizumab 300 mg q4w	NR	NR	NR	NR	NR	NR	NR	NR	UAS7	Some concerns
Maurer et al., 2024	2	RCT; DB (*N* = 160)	12	Rilzabrutinib 400 mg qd, bid or tid	NR	NR	NR	NR	NR	NR	NR	NR	ISS7, UAS7	Some concerns

Abbreviations: AEs, advent events; bid, twice daily; CU‐Q2oL, chronic urticaria quality of life question‐naire; DB, double‐blind; DLQI, dermatology life quality index; HSS7, weekly hive severity score; ISS7, weekly itch severity score; NR, not reported; OL, open label; q2w, every 2 weeks; q4w, every 4 weeks; qd, once daily; RCT, randomized controll trial; SAEs, serious advent events; SD: standard deviation; TB, triple‐blind; tid, three times daily; UAS7, the weekly urticaria activity score; UCT, urticaria control test.

### 3.1 Outcomes

#### 3.1.1 Weekly UAS7

**FIGURE 1:**
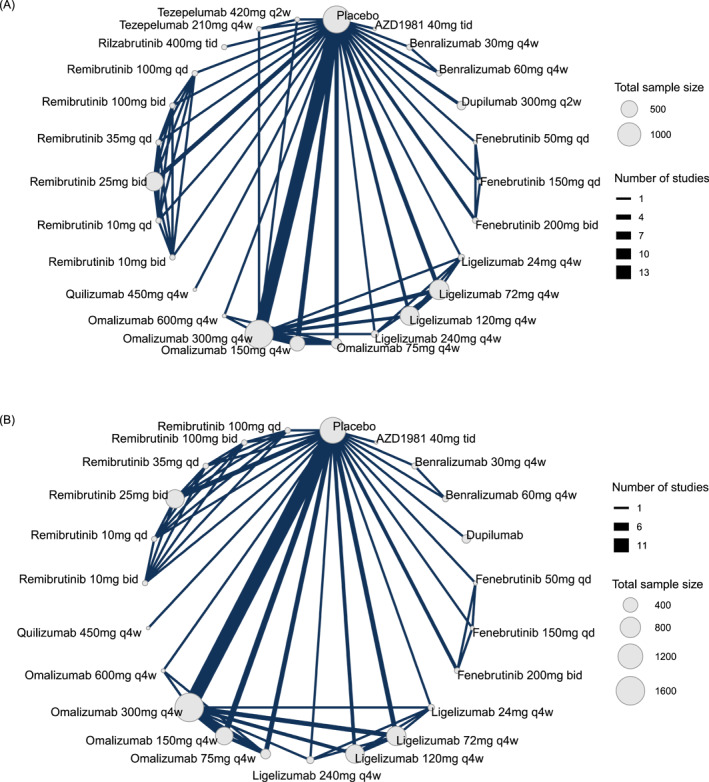
Network graph of studies included in the network. (A) Change from baseline in the weekly urticaria activity score, and (B) the occurrence of advent events. The width of each line connecting 2 treatments (nodes) is proportional to the number of head‐to‐head trials for that comparison. Qd indicates once daily; bid, twice daily; tid, three times daily; q4w, every 4 weeks.

**FIGURE 2:**
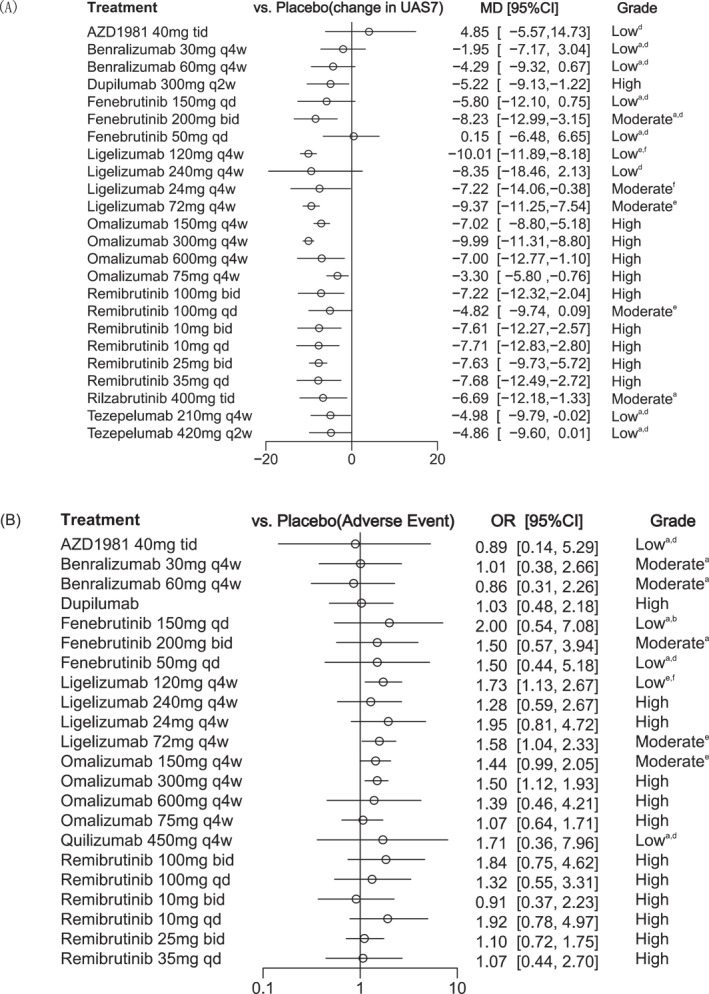
Forest plot for the network analysis. a: Within‐study bias; b: Reporting bias; c: Indirectness; d: Imprecision; e: Heterogeneity; f: Incoherence. Forest plots showing (A) change from baseline in the weekly urticaria activity score, and (B) the occurrence of advent events. Results are presented for medications versus placebo. Results are presented as mean difference and odds ratios with 95% credible intervals (95% CI).

In the network analysis, 20 studies involving 6313 patients^12^, ^13^, ^14^, ^15^, ^16^, ^17^, ^18^, ^20^, ^24^, ^25^, ^26^, ^27^, ^28^, ^29^, ^30^, ^31^, ^34^, ^50^, ^51^, ^52^ randomized to 26 different treatments or dosages assessed change from baseline in the weekly UAS7, while 12 studies with 3281 patients^7^, ^12^, ^14^, ^17^, ^18^, ^19^, ^20^, ^24^, ^28^, ^29^, ^31^, ^51^ evaluated the proportions of patients who achieved well‐controlled disease (UAS7 ≤ 6) and 13 studies with 5430 patients^12^, ^14^, ^18^, ^19^, ^20^, ^24^, ^28^, ^29^, ^31^, ^34^, ^50^, ^51^, ^52^ evaluated the proportion achieving complete response (UAS7 = 0). Figure 1A, Supporting Information S1: eFigures 3 and 5 show the structure of the network and how these treatments were compared with each other in the original studies. Placebo was used by most studies as a comparator, several studies compared different dosages of the same agent, and only 3 studies directly compared different systemic medications. Figure 2A, Supporting Information S1: eFigures 4 and 6 show the efficacy of the different drugs in reducing UAS7 and the proportion of patients who achieved UAS7 ≤ 6 or UAS7 = 0 compared to placebo. Estimates for all possible treatment comparisons are presented in Supporting Information S1: eTables 2, 4, and 6.

##### 3.1.1.1 Change from baseline in UAS7

For the change from baseline in UAS7 compared with placebo, the NMA estimates illustrated that the most efficacious treatments were achieved with ligelizumab 120 mg [MD −10.01, 95% CI (−11.89; −8.18)], omalizumab 300 mg [MD −9.99, 95% CI (−11.31; −8.80)] and ligelizumab 72 mg [MD −9.37, 95% CI (−11.25; −7.54)]. Additionally, fenebrutinib 200 mg [MD −8.23, 95% CI (−12.99; −3.15)], along with remibrutinib administered at various dosages of 35 mg once daily [MD −7.80, 95% CI (−12.76; −2.51)], 25 mg twice daily [MD −7.69, 95% CI (−9.85; −5.76)], and 10 mg twice daily [MD −7.61, 95% CI (−12.59; −2.47)], demonstrated significant efficacy in reducing UAS7 compared to placebo. Other treatments, such as rilzabrutinib 400 mg [MD −6.68, 95% CI (−12.08; −1.12)], dupilumab 300 mg [MD −5.22, 95% CI (−9.13; −1.22)], and tezepelumab 210 mg [MD −4.98, 95% CI (−9.79; −0.02)], were also more effective than placebo. However, no statistical difference in efficacy was observed for AZD1981 and benralizumab compared with placebo.

##### 3.1.1.2 Proportion of patients achieved UAS7 ≤ 6

In terms of the proportions of patients achieving UAS7 ≤ 6, omalizumab 300 mg administered every 4 weeks was ranked as the most effective available treatment, demonstrating a ratio of 7.41 (95% CI: 5.15; 10.90) compared to placebo. Omalizumab 150 mg given every 4 weeks also showed significant efficacy, with a ratio of 4.72 (95% CI: 3.13; 7.12). Additionally, fenebrutinib 200 mg taken twice daily exhibited a ratio of 4.09 (95% CI: 1.43; 11.40), while remibrutinib 25 mg dosed twice daily revealed a ratio of 3.32 (95% CI: 2.25; 4.95) relative to placebo. Data for this metric of the AZD were lacking. Similar to the change from baseline in UAS7, there was also no significant difference in the efficacy of benralizumab compared with placebo.

##### 3.1.1.3 Proportion of patients achieved UAS7 = 0

Among all included interventions, the proportion of patients achieving UAS7 = 0 demonstrated a strong advantage with omalizumab 300 mg [OR 7.11, 95% CI (4.28; 19.40)], and oral remibrutinib 25 mg twice daily [OR 4.46, 95% CI (2.10; 9.99)]. Omalizumab 150 mg [OR 3.25, 95% CI (1.53; 7.11)] achieved a relatively smaller proportion of patients with UAS7 = 0 compared to the above drugs. Additionally, no statistically significant differences in efficacy were observed for the other drugs compared with placebo in this metric. Fenebrutinib and omalizumab 75 mg are not shown in the forest plot due to large confidence intervals, with specific values provided in Supporting Information S1: eTable 6. SUCRA values for the change in UAS7 from baseline and the proportion of patients achieving UAS7 = 0 are presented in Supporting Information S1: eFigure 15.

#### 3.1.2 Adverse events and serious adverse events

In total, 22 studies were included in the assessment of AEs and SAEs. Figure 1B and Supporting Information S1: eFigure 7 illustrate the network structure. Figure 2B and Supporting Information S1: eFigure 8 lists the estimated odds ratios for the occurrence of AEs for all medications using placebo as the comparator. Fenebrutinib 150 mg daily, with an odds ratio of 2.00 (95% CI: 0.54; 7.08), was ranked as the least safe in terms of AE frequency. Compared to placebo, benralizumab 60 mg given subcutaneously every 4 weeks had an odds ratio of 0.86 (95% CI: 0.31; 2.26), whereas remibrutinib at doses of 10 and 25 mg administered orally twice daily had odds ratios of 0.91 (95% CI: 0.37; 2.23) and 1.10 (95% CI: 0.72; 1.75), respectively. We noted that individuals receiving ligelizumab 120 mg [OR 1.73, 95% CI (1.13; 2.67)], ligelizumab 72 mg [OR 1.58, 95% CI (1.04; 2.33)], and omalizumab 300 mg [OR 1.50, 95% CI (1.12; 1.93)] experienced a significantly higher risk of AEs compared to placebo. Estimates for all possible treatment comparisons are presented in Supporting Information S1: eTables 8 and 10. Further subgroup analyses based on treatment duration and adult age revealed no significant differences in AE occurrence among the treatment comparisons (Supporting Information S1: eFigure 18D). Similarly, no significant differences were found in SAEs across all treatment comparisons (Supporting Information S1: eFigure 8). The combined SUCRA values for AEs and SAEs showed that ligelizumab 120 mg was ranked as the least desirable treatment, showing a higher risk of both AEs and SAEs (Supporting Information S1: eFigure 17).

**FIGURE 3:**
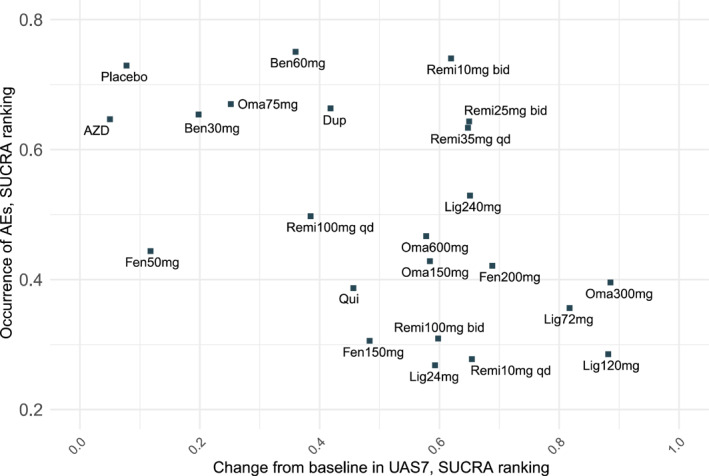
Cluster ranking plot for the efficacy and safety of treatments for chronic spontaneous urticaria in network meta‐analyses. Both in efficacy and safety, a higher score on the lower surface under the cumulative ranking curve indicates better efficacy and higher safety.

Taken together, based on the SUCRA values for efficacy and incidence of AEs shown in Figure 3, no drug was found to be optimal for both efficacy and safety. Omalizumab 300 mg every 4 weeks still exhibited notable efficacy. However, when considering both benefits and harms together, the network estimates illustrated that remibrutinib, at doses of 35 mg daily, 25 mg twice daily, and 10 mg twice daily, showed the best overall performance.

#### 3.1.3 Other outcomes

The complete network analysis also includes ISS7, HSS7, and DLQI, which showed similar results to the above, as detailed in Supporting Information S1: eFigures 9–14 and eTables 11–13. The combined SUCRA values for ISS7 and HSS7 are shown in Supporting Information S1: eFigure 16.

### 3.2 Quality of evidence

The global *I* ^*2*^ statistic indicated no significant heterogeneity within the network (*I* ^*2*^ < 50%), detailed in Supporting Information S1: eTable 14. Net splitting showed inconsistency in the network for the comparisons between ligelizumab in different dosages and placebo (Supporting Information S1: eFigure 19). Egger's regression test did not show publication bias across studies (*p* > 0.05). However, the comparison‐adjusted funnel plot symmetry indicated small‐study effects for most outcomes, except for the change in the weekly HSS7 (Supporting Information S1: eFigure 20). The evidence certainty grading according to the GRADE approach, along with the reasons for downgrading each treatment, is detailed in Supporting Information S1: eTables 3, 5, 7 and 9 in the Supplement. Most treatment comparisons had limited evidence certainty due to within‐study bias, imprecision, heterogeneity, and incoherence.

### 3.3 Subgroup and sensitivity analysis

Sixteen studies included only adults,^7^, ^12^, ^13^, ^16^, ^18^, ^19^, ^24^, ^25^, ^26^, ^27^, ^29^, ^30^, ^31^, ^49^, ^50^, ^51^, ^52^ one included adolescents,^52^ and six included both adults and adolescents.^14^, ^15^, ^17^, ^20^, ^28^, ^34^ Subgroup analysis restricted to studies with only adults showed no significant differences compared to the overall dataset except for AEs.

Regarding treatment duration, limited efficacy data were available on long‐term treatment (>16 weeks),^28^, ^30^ and subgroup analyses focusing on studies with ≤16 weeks showed no statistically significant differences from the overall results. In terms of AEs, 10 studies were performed with treatment durations ≤16 weeks^15^, ^16^, ^17^, ^19^, ^20^, ^25^, ^26^, ^27^, ^29^, ^51^ and subgroup analyses of these treatments showed no statistically significant differences in safety compared to placebo, whereas for studies with treatment durations >16 weeks, the subgroup analysis indicated a slightly increased risk of AEs for omalizumab 300 mg every 4 weeks [OR 1.59, 95% CI (1.07; 2.22)] and ligelizumab 120 mg every 4 weeks [OR 1.75, 95% CI (1.02; 2.96)] compared to placebo.

Post hoc analysis excluding studies with a high overall risk of bias could not be included as none of the included studies were assessed to have a high risk of bias. Findings from other sensitivity analyses were robust and did not substantially change from the main findings. Specific details of the subgroup analyses and sensitivity analyses are provided in Supporting Information S1: eFigure 18.

## 4 DISCUSSION

In this systematic review and NMA, we combined direct and indirect evidence from 23 randomized clinical trials of 6933 participants with CSU. The systemic drugs for CSU mainly include biological agents and small molecule drugs. Previous meta‐analysis studies^53^ investigating conventional systemic drugs indicated that cyclosporine A combined with antihistamine resulted in greater improvements regarding the UAS7 among CSU patients. However, conventional systemic drugs have a wide range of pharmacological effects and can induce immunosuppression, raising safety concerns that may limit their clinical application. As biologics and small molecule drugs are more targeted in their mechanisms of action, their efficacy can provide valuable insights into the role of specific targets in the pathogenesis of CSU. Regarding biologics, previous NMA revealed that the biological agents ligelizumab 72 or 240 mg and omalizumab 300 or 600 mg are effective treatments for CSU.^32^, ^33^ Given the extensive ongoing clinical trials in CSU, an updated analysis of the findings is necessary. Our study provides updated evidence of established biological agents and novel small molecule drugs, with the strength of comprehensive systematic review, robust design, and a focus on clinically relevant symptoms of urticaria.

In terms of efficacy, our findings are in line with previous pairwise meta‐analyse,^33^, ^39^, ^54^ demonstrating the benefits of omalizumab, ligelizumab and remibrutinib, which highlights the critical role of free immunoglobulin E in the pathogenesis of CSU. In a previous NMA, the most efficacious treatments were achieved with ligelizumab, 72 or 240 mg and omalizumab, 300 or 600 mg^32^; in our updated NMA, omalizumab 300 mg and ligelizumab 120 mg administered every 4 weeks showed the best efficacy. However, the safety profile analysis in our study indicated that individuals receiving ligelizumab 120 mg or 72 mg, and omalizumab 300 mg experienced a significantly higher risk of AEs compared to placebo, which is different from the previous results, in which omalizumab 150 mg [OR 1.44, 95% CI (1.02–2.03)], experienced a statistically significantly higher risk of AEs compared with placebo, but ligelizumab 24 mg [OR 0.44, 95% CI (0.21; 0.93)], showed a protective effect.^32^ Most AEs reported were mild to moderate. Based on the results from subgroup analyses, this discrepancy may be related to longer treatment durations and the inclusion of adolescent populations. Our findings suggest that additional data are needed to evaluate the safety of omalizumab in specific populations and with long‐term treatment durations. Notably, the Phase III trial (PEARL) of ligelizumab confirmed that it can achieve clinically meaningful improvements in disease activity, but it did not demonstrate superiority over omalizumab.^35^ Although the Phase IIb trial showed a higher percentage of patients achieving complete control of CSU symptoms,^37^ this led to its discontinuation. Since it will not be available to the public in the future, it is not recommended in this article.

Furthermore, the results of this NMA showed that remibrutinib strikes a good balance between efficacy and safety, highlighting the potential of this novel BTK inhibitor, in which phase 3 studies (NCT05032157 and NCT05030311) have been completed and showed promising efficacy in H1‐AH refractory CSU. We know that two endotypes of CSU have been identified: type I autoimmune CSU (autoallergic) driven by IgE against autoallergens and type IIb autoimmune CSU associated with IgG against autoantibodies lgE or FcεRI.^55^ The two CSU endotypes exhibit distinct clinical responses to anti‐IgE therapy.^56^ In both types, FcεRI cross‐linking‐mediated mast cell and basophil activation and degranulation is a hallmark of pathogenesis.^29^, ^57^ This cross‐linking promptly activates Bruton's tyrosine kinase (BTK), a cytoplasmic kinase downstream of the IgE receptor, which is expressed in mast cells, basophils, and B cells. Therefore, BTK may be particularly effective in type IIb CSU patients who have higher disease activity, severity, and a poorer response to conventional therapy.^29^ Remibrutinib provides rapid and effective disease control with a favorable safety profile, and therefore has the potential to be an attractive alternative therapy for patients as well as the preferred oral treatment option for patients with moderate‐to‐severe CSU.^24^, ^29^

There are several limitations in this NMA. Firstly, this analysis was constrained by differences in the design of the included trials, such as variations in patient populations and dosing schedules, which can affect the comparability of the results across studies. Secondly, some treatment comparisons were based on a single trial or trials with small sample sizes, potentially limiting the robustness of the findings. Thirdly, there is limited evidence for comparisons involving active comparators, reducing the ability to draw definitive conclusions about the relative efficacy of different treatments. Additionally, the underrepresentation of certain populations in clinical trials, including adolescents, older patients and patients with comorbidities, may limit the generalizability to populations. This underrepresentation means that the results may not be fully applicable to these groups, who may respond differently to the treatments. Given these limitations, our results should be interpreted with caution. Further research is warranted, particularly head‐to‐head trials comparing active comparators to provide more direct evidence of relative efficacy. Long‐term safety trials are also needed to assess the prolonged use of these treatments. Moreover, considering the high costs associated with novel therapies, research on cost‐effectiveness is crucial to inform healthcare decision‐making and ensure that treatments are accessible and affordable.

## 5 CONCLUSION

Overall, the results of this systematic review and network meta‐analysis suggest that the biological agent omalizumab 300 mg and the oral small molecule remibrutinib, administered at doses of 35 mg once daily, 25 mg twice daily, and 10 mg twice daily, are effective pharmacologic treatments with respect to the benefits and harms of H1‐AH refractory CSU.

## AUTHOR CONTRIBUTIONS

**Zuotao Zhao:** Writing—review and editing; conceptualization; supervision; funding acquisition. **Yaqi Zheng:** Writing—original draft; writing—review and editing; software; formal analysis; investigation; methodology; data curation; validation; visualization; project administration; resources. **Xiaoting Song:** Writing—review and editing; writing—original draft. **Chengyue Peng:** Investigation. **Shuanglu Liao:** Investigation. **Peixin Zhang:** Investigation. **Yen Tan:** Investigation. **Xiaojie Huang:** Writing—review and editing. **Litao Zhang:** Writing—review and editing.

## CONFLICT OF INTEREST STATEMENT

Dr. Zhao is the speaker/advisor for and/or has received research funding from Novartis, Pfizer, Astellas, Galderma, Janssen, GSK, BAYER, LEO, MEDA Pharma and ALK Pharma outside the submitted work. The rest of the authors declare that they have no conflicts of interest.

## Supporting information

Supporting Information S1

## Data Availability

All data generated or analyzed during this study are included in this article. Further inquiries can be directed to the corresponding authors.
