# Supplementary material for: Biological and target synthetic treatments for chronic spontaneous urticaria: A systematic review and network meta‐analysis
Source: Clin Transl Allergy. 2025 May 6;15(5):e70052. doi: 10.1002/clt2.70052 (PMC12053928; doi:10.1002/clt2.70052)

***Supplementary information***

**eTable 1.** Systematic review search strategy.

**eFigure 1.** PRISMA flowchart for the detailed process of study selection.

**eFigure 2.** Risk of bias graph

**eTable 2.** League table and the Surface Under the Cumulative Ranking (SUCRA): change from baseline in the weekly urticaria activity score (UAS7).

**eTable 3.** GRADE assessment by CINeMA: change from baseline in the weekly urticaria activity score (UAS7).

**eFigure 3**. Network graphs for proportion of patients achieved well-controlled disease (UAS7≤6).

**eFigure 4**. Forest plot for proportion of patients achieved well-controlled disease (UAS7≤6).

**{Bei, 2023 #1277}eTable 4.** League table and the Surface Under the Cumulative Ranking (SUCRA): proportion of patients achieved well-controlled disease (UAS7≤6).

**eTable 5.** GRADE assessment by CINeMA: proportion of patients achieved well-controlled disease (UAS7≤6).

**eFigure 5**. Network graphs for proportion of patients achieved complete response (UAS7=0).

**eFigure 6**. Forest plot for proportion of patients achieved complete response (UAS7=0).

**eTable 6.** League table and the Surface Under the Cumulative Ranking (SUCRA): proportion of patients achieved complete response (UAS7=0).

**eTable 7.** GRADE assessment by CINeMA: proportion of patients achieved complete response (UAS7=0).

**eTable 8.** League table and the Surface Under the Cumulative Ranking (SUCRA): adverse events (AEs).

**eTable 9.** GRADE assessment by CINeMA: adverse events (AEs).

**eFigure 7**. Network graphs for the incidence of serious adverse events (SAEs).

**eFigure 8**. Forest plot for the incidence of serious adverse events (SAEs).

**eTable 10.** League table and the Surface Under the Cumulative Ranking (SUCRA): serious adverse events (SAEs).

**eFigure 9**. Network graphs for the change from baseline in the weekly itch severity score (ISS7).

**eFigure 10**. Forest plot for the change from baseline in the weekly itch severity score (ISS7).

**eTable 11.** League table and the Surface Under the Cumulative Ranking (SUCRA): change from baseline in the weekly itch severity score (ISS7).

**eFigure 11**. Network graphs for the change from baseline in the weekly hive severity score (HSS7).

**eFigure 12**. Forest plot for the change from baseline in the weekly hive severity score (HSS7).

**eTable 12.** League table and the Surface Under the Cumulative Ranking (SUCRA): change from baseline in the weekly hive severity score (HSS7).

**eFigure 13**. Network graphs for the change from baseline in the dermatology life quality index (DLQI).

**eFigure 14**. Forest plot for the change from baseline in the dermatology life quality index (DLQI).

**eTable 13.** League table and the Surface Under the Cumulative Ranking (SUCRA): change from baseline in the dermatology life quality index (DLQI).

**eFigure 15.** Cluster ranking plot of change in weekly urticaria activity score (UAS7) and proportion of patients achieved complete response (UAS7=0)

**eFigure 16.** Cluster ranking plot of itch severity and hives severity.

**eFigure 17.** Cluster ranking plot of adverse events and serious adverse events.

**eTable 14.** Analysis of heterogeneity.

**eFigure 18.** Subgroup and sensitivity analysis.

**eFigure 19.** Evaluation of inconsistency: node-splitting model.

**eFigure 20.** Comparison-adjusted funnel plot.

**eTable 1.** Search Strategy from PubMed.

| **Search** | **Query** | **Results** |
| --- | --- | --- |
| 30 | (((((((((((((("Urticaria"[Mesh]) OR (urticaria[Title/Abstract])) OR (Hive*[Title/Abstract])) OR (Wheal*[Title/Abstract])) OR (chronic Urticaria[Title/Abstract])) OR (chronic spontaneous urticaria[Title/Abstract])) OR (chronic idiopathic urticaria[Title/Abstract])) OR (Autoimmune Urticaria*[Title/Abstract])) OR (refractory urticaria[Title/Abstract])) OR (antihistamin* resistant chronic urticaria[Title/Abstract])) OR (CU[Title/Abstract])) OR (CSU[Title/Abstract])) OR (CIU[Title/Abstract])) AND ((((((((((((((((("Omalizumab"[Mesh]) OR (Omalizumab[Title/Abstract])) OR (Xolair[Title/Abstract])) OR (((("ligelizumab" [Supplementary Concept]) OR (ligelizumab[Title/Abstract])) OR (QGE-031[Title/Abstract])) OR (QGE031[Title/Abstract]))) OR (UB-221[Title/Abstract])) OR (Quilizumab[Title/Abstract])) OR (((((("mepolizumab" [Supplementary Concept]) OR (mepolizumab[Title/Abstract])) OR (Bosatria[Title/Abstract])) OR (SB-240563[Title/Abstract])) OR (SB240563[Title/Abstract])) OR (Nucala[Title/Abstract]))) OR (Vixarelimab[Title/Abstract])) OR ((((((("dupilumab" [Supplementary Concept]) OR (dupilumab[Title/Abstract])) OR (SAR231893[Title/Abstract])) OR (SAR-231893[Title/Abstract])) OR (Dupixent[Title/Abstract])) OR (REGN668[Title/Abstract])) OR (REGN-668[Title/Abstract]))) OR (((((("benralizumab" [Supplementary Concept]) OR (benralizumab[Title/Abstract])) OR (MEDI-563[Title/Abstract])) OR (MEDI 563[Title/Abstract])) OR (Fasenra[Title/Abstract])) OR (BIW-8405[Title/Abstract]))) OR (AZD 1981[Title/Abstract])) OR (Barzolvolimab[Title/Abstract])) OR (Lirentelimab[Title/Abstract])) OR (((("remibrutinib" [Supplementary Concept]) OR (Remibrutinib[Title/Abstract])) OR (LOU064[Title/Abstract])) OR (N-(3-(6-amino-5-(2-(methyl(prop-2-enoyl)amino)ethoxy)pyrimidin-4-yl)-5-fluoro-2-methylphenyl)-4-cyclopropyl-2-fluorobenzamide[Title/Abstract]))) OR (Rilzabrutinib[Title/Abstract])) OR (((("fenebrutinib" [Supplementary Concept]) OR (fenebrutinib[Title/Abstract])) OR (2-(3'-(hydroxymethyl)-1-methyl-5-((5-(2-methyl-4-(oxetan-3-yl)piperazin-1-yl)pyridin-2-yl)amino)-6-oxo-1,6-dihydro-(3,4'-bipyridin)-2'-yl)-7,7-dimethyl-3,4,7,8-tetrahydro-2H-cyclopenta(4,5)pyrrolo(1,2-a)pyrazin-1(6H)-one[Title/Abstract])) OR (GDC-0853[Title/Abstract]))) OR (((((((("tezepelumab" [Supplementary Concept]) OR (tezepelumab[Title/Abstract])) OR (tezepelumab-ekko[Title/Abstract])) OR (AMG-157[Title/Abstract])) OR (tezspire[Title/Abstract])) OR (MEDI-9929[Title/Abstract])) OR (MEDI-19929[Title/Abstract])) OR (MEDI9929[Title/Abstract])))) AND ((randomized controlled trial[pt] OR controlled clinical trial[pt] OR clinical trials as topic[mesh:noexp] OR trial[ti] OR random*[tiab] OR placebo*[tiab])) | 160 |
| 29 | (((((((((((("Urticaria"[Mesh]) OR (urticaria[Title/Abstract])) OR (Hive*[Title/Abstract])) OR (Wheal*[Title/Abstract])) OR (chronic Urticaria[Title/Abstract])) OR (chronic spontaneous urticaria[Title/Abstract])) OR (chronic idiopathic urticaria[Title/Abstract])) OR (Autoimmune Urticaria*[Title/Abstract])) OR (refractory urticaria[Title/Abstract])) OR (antihistamin* resistant chronic urticaria[Title/Abstract])) OR (CU[Title/Abstract])) OR (CSU[Title/Abstract])) OR (CIU[Title/Abstract]) | 174,005 |
| 26 | (randomized controlled trial[pt] OR controlled clinical trial[pt] OR clinical trials as topic[mesh:noexp] OR trial[ti] OR random*[tiab] OR placebo*[tiab]) | 1,979,984 |
| 25 | (((((((((((((((("Omalizumab"[Mesh]) OR (Omalizumab[Title/Abstract])) OR (Xolair[Title/Abstract])) OR (((("ligelizumab" [Supplementary Concept]) OR (ligelizumab[Title/Abstract])) OR (QGE-031[Title/Abstract])) OR (QGE031[Title/Abstract]))) OR (UB-221[Title/Abstract])) OR (Quilizumab[Title/Abstract])) OR (((((("mepolizumab" [Supplementary Concept]) OR (mepolizumab[Title/Abstract])) OR (Bosatria[Title/Abstract])) OR (SB-240563[Title/Abstract])) OR (SB240563[Title/Abstract])) OR (Nucala[Title/Abstract]))) OR (Vixarelimab[Title/Abstract])) OR ((((((("dupilumab" [Supplementary Concept]) OR (dupilumab[Title/Abstract])) OR (SAR231893[Title/Abstract])) OR (SAR-231893[Title/Abstract])) OR (Dupixent[Title/Abstract])) OR (REGN668[Title/Abstract])) OR (REGN-668[Title/Abstract]))) OR (((((("benralizumab" [Supplementary Concept]) OR (benralizumab[Title/Abstract])) OR (MEDI-563[Title/Abstract])) OR (MEDI 563[Title/Abstract])) OR (Fasenra[Title/Abstract])) OR (BIW-8405[Title/Abstract]))) OR (AZD 1981[Title/Abstract])) OR (Barzolvolimab[Title/Abstract])) OR (Lirentelimab[Title/Abstract])) OR (((("remibrutinib" [Supplementary Concept]) OR (Remibrutinib[Title/Abstract])) OR (LOU064[Title/Abstract])) OR (N-(3-(6-amino-5-(2-(methyl(prop-2-enoyl)amino)ethoxy)pyrimidin-4-yl)-5-fluoro-2-methylphenyl)-4-cyclopropyl-2-fluorobenzamide[Title/Abstract]))) OR (Rilzabrutinib[Title/Abstract])) OR (((("fenebrutinib" [Supplementary Concept]) OR (fenebrutinib[Title/Abstract])) OR (2-(3'-(hydroxymethyl)-1-methyl-5-((5-(2-methyl-4-(oxetan-3-yl)piperazin-1-yl)pyridin-2-yl)amino)-6-oxo-1,6-dihydro-(3,4'-bipyridin)-2'-yl)-7,7-dimethyl-3,4,7,8-tetrahydro-2H-cyclopenta(4,5)pyrrolo(1,2-a)pyrazin-1(6H)-one[Title/Abstract])) OR (GDC-0853[Title/Abstract]))) OR (((((((("tezepelumab" [Supplementary Concept]) OR (tezepelumab[Title/Abstract])) OR (tezepelumab-ekko[Title/Abstract])) OR (AMG-157[Title/Abstract])) OR (tezspire[Title/Abstract])) OR (MEDI-9929[Title/Abstract])) OR (MEDI-19929[Title/Abstract])) OR (MEDI9929[Title/Abstract])) | 7,982 |
| 23 | ((((((("tezepelumab" [Supplementary Concept]) OR (tezepelumab[Title/Abstract])) OR (tezepelumab-ekko[Title/Abstract])) OR (AMG-157[Title/Abstract])) OR (tezspire[Title/Abstract])) OR (MEDI-9929[Title/Abstract])) OR (MEDI-19929[Title/Abstract])) OR (MEDI9929[Title/Abstract]) | 203 |
| 22 | ((("fenebrutinib" [Supplementary Concept]) OR (fenebrutinib[Title/Abstract])) OR (2-(3'-(hydroxymethyl)-1-methyl-5-((5-(2-methyl-4-(oxetan-3-yl)piperazin-1-yl)pyridin-2-yl)amino)-6-oxo-1,6-dihydro-(3,4'-bipyridin)-2'-yl)-7,7-dimethyl-3,4,7,8-tetrahydro-2H-cyclopenta(4,5)pyrrolo(1,2-a)pyrazin-1(6H)-one[Title/Abstract])) OR (GDC-0853[Title/Abstract]) | 48 |
| 21 | Rilzabrutinib[Title/Abstract] | 29 |
| 20 | ((("remibrutinib" [Supplementary Concept]) OR (Remibrutinib[Title/Abstract])) OR (LOU064[Title/Abstract])) OR (N-(3-(6-amino-5-(2-(methyl(prop-2-enoyl)amino)ethoxy)pyrimidin-4-yl)-5-fluoro-2-methylphenyl)-4-cyclopropyl-2-fluorobenzamide[Title/Abstract]) | 22 |
| 19 | "remibrutinib" [Supplementary Concept] | 6 |
| 18 | Lirentelimab[Title/Abstract] | 23 |
| 17 | Barzolvolimab[Title/Abstract] | 3 |
| 16 | AZD 1981[Title/Abstract] | 2 |
| 15 | ((((("benralizumab" [Supplementary Concept]) OR (benralizumab[Title/Abstract])) OR (MEDI-563[Title/Abstract])) OR (MEDI 563[Title/Abstract])) OR (Fasenra[Title/Abstract])) OR (BIW-8405[Title/Abstract]) | 860 |
| 14 | "benralizumab" [Supplementary Concept] | 348 |
| 13 | (((((("dupilumab" [Supplementary Concept]) OR (dupilumab[Title/Abstract])) OR (SAR231893[Title/Abstract])) OR (SAR-231893[Title/Abstract])) OR (Dupixent[Title/Abstract])) OR (REGN668[Title/Abstract])) OR (REGN-668[Title/Abstract]) | 2,993 |
| 12 | "dupilumab" [Supplementary Concept] | 1,566 |
| 11 | Vixarelimab[Title/Abstract] | 3 |
| 10 | ((((("mepolizumab" [Supplementary Concept]) OR (mepolizumab[Title/Abstract])) OR (Bosatria[Title/Abstract])) OR (SB-240563[Title/Abstract])) OR (SB240563[Title/Abstract])) OR (Nucala[Title/Abstract]) | 1,547 |
| 9 | "mepolizumab" [Supplementary Concept] | 671 |
| 8 | "tezepelumab" [Supplementary Concept] | 85 |
| 7 | Quilizumab[Title/Abstract] | 9 |
| 6 | UB-221[Title/Abstract] | 5 |
| 5 | ((("ligelizumab" [Supplementary Concept]) OR (ligelizumab[Title/Abstract])) OR (QGE-031[Title/Abstract])) OR (QGE031[Title/Abstract]) | 64 |
| 4 | "ligelizumab" [Supplementary Concept] | 30 |
| 3 | (("Omalizumab"[Mesh]) OR (Omalizumab[Title/Abstract])) OR (Xolair[Title/Abstract]) | 3,864 |
| 2 | "Omalizumab"[Mesh] | 2,469 |

**eTable 1.** Search Strategy from Embase. (continued)

| **Search** | **Query** | **Results** |
| --- | --- | --- |
| #51 | #3 AND #45 AND #50 | 605 |
| #50 | #48 AND #49 | 2451463 |
| #49 | [embase]/lim | 31667357 |
| #48 | #46 OR #47 | 2909182 |
| #47 | 'controlled clinical trial':ab,ti OR random*:ab,ti OR placebo*:ab,ti OR trial:ab,ti | 2686680 |
| #46 | 'controlled clinical trial'/exp | 997325 |
| #45 | #6 OR #9 OR #10 OR #13 OR #16 OR #19 OR #22 OR #25 OR #28 OR #29 OR #32 OR #35 OR #38 OR #41 OR #44 | 22102 |
| #44 | #42 OR #43 | 204 |
| #43 | fenebrutinib:ab,ti OR ('2 [3`':ab,ti AND hydroxymethyl:ab,ti AND '1 methyl 5 [ [5 [2 methyl 4':ab,ti AND '3 oxetanyl':ab,ti AND '1 piperazinyl] 2 pyridinyl] amino] 6 oxo 1, 6 dihydro':ab,ti AND '3, 4` bipyridin':ab,ti AND '2` yl] 7, 7 dimethyl 3, 4, 7, 8 tetrahydro 2h cyclopenta':ab,ti AND '4, 5':ab,ti AND pyrrolo:ab,ti AND '1, 2 a':ab,ti AND 'pyrazin 1':ab,ti AND 6h:ab,ti AND one:ab,ti) OR ('2 [3`':ab,ti AND hydroxymethyl:ab,ti AND '1 methyl 5 [ [5 [2 methyl 4':ab,ti AND 'oxetan 3 yl':ab,ti AND 'piperazin 1 yl] pyridin 2 yl] amino] 6 oxo 1, 6 dihydro':ab,ti AND '3, 4` bipyridin':ab,ti AND '2` yl] 7, 7 dimethyl 3, 4, 7, 8 tetrahydro 2h cyclopenta':ab,ti AND '4, 5':ab,ti AND pyrrolo:ab,ti AND '1, 2 a':ab,ti AND 'pyrazin 1':ab,ti AND 6h:ab,ti AND one:ab,ti) OR (hydroxymethyl:ab,ti AND ', 6':ab,ti AND 'tetramethyl 1':ab,ti AND 4:ab,ti AND 7:ab,ti AND ', 1':ab,ti AND 8:ab,ti AND 'tetrahydro 4 aza 1':ab,ti AND 2:ab,ti AND 'cyclopenta [4, 5] pyrrolo [1, 2 a] pyrazina 5':ab,ti AND '1, 4':ab,ti AND 'piperazina 2':ab,ti AND '2, 4':ab,ti AND '3, 5':ab,ti AND ', 5':ab,ti AND '2, 5':ab,ti AND 'tripyridina 7':ab,ti AND 'oxetanaheptaphane 1':ab,ti AND ', 3':ab,ti AND 6:ab,ti AND 3:ab,ti AND 1:ab,ti AND h:ab,ti AND dione:ab,ti) OR ('3 [3':ab,ti AND hydroxymethyl:ab,ti AND '4 [1 methyl 5 [ [5 [2 methyl 4':ab,ti AND '3 oxetanyl':ab,ti AND '1 piperazinyl] 2 pyridinyl] amino] 6 oxo 3 pyridinyl] 2 pyridinyl] 7, 7 dimethyl 1, 2, 6, 8 tetrahydrocyclopenta [3, 4] pyrrolo [3, 5 b] pyrazin 4 one':ab,ti) OR ('3 [3':ab,ti AND hydroxymethyl:ab,ti AND '4 [1 methyl 5 [ [5 [2 methyl 4':ab,ti AND 'oxetan 3 yl':ab,ti AND 'piperazin 1 yl] pyridin 2 yl] amino] 6 oxopyridin 3 yl] pyridin 2 yl] 7, 7 dimethyl 1, 2, 6, 8 tetrahydrocyclopenta [3, 4] pyrrolo [3, 5 b] pyrazin 4 one':ab,ti) OR 'g 02599853':ab,ti OR 'g 2599853':ab,ti OR g02599853:ab,ti OR g2599853:ab,ti OR 'gdc 0853':ab,ti OR 'gdc 853':ab,ti OR gdc0853:ab,ti OR gdc853:ab,ti OR 'rg 7845':ab,ti OR rg7845:ab,ti OR 'ro 7010939':ab,ti OR ro7010939:ab,ti | 77 |
| #42 | 'fenebrutinib'/exp | 197 |
| #41 | #39 OR #40 | 133 |
| #40 | rilzabrutinib:ab,ti OR ('2 [3 [4 amino 3':ab,ti AND '2 fluoro 4 phenoxyphenyl':ab,ti AND '1h pyrazolo [3, 4 d] pyrimidin 1 yl] 1 piperidinylcarbonyl] 4 methyl 4 [4':ab,ti AND '3 oxetanyl':ab,ti AND '1 piperazinyl] 2 pentenenitrile':ab,ti) OR ('2 [3 [4 amino 3':ab,ti AND '2 fluoro 4 phenoxyphenyl':ab,ti AND '1h pyrazolo [3, 4 d] pyrimidin 1 yl] piperidine 1 carbonyl] 4 methyl 4 [4':ab,ti AND 'oxetan 3 yl':ab,ti AND 'piperazin 1 yl] pent 2 enenitrile':ab,ti) OR ('3 [4 amino 3':ab,ti AND '2 fluoro 4 phenoxyphenyl':ab,ti AND '1h pyrazolo [3, 4 d] pyrimidin 1 yl] alpha [2 methyl 2 [4':ab,ti AND '3 oxetanyl':ab,ti AND '1 piperazinyl] propylidene] beta oxo 1 piperidinepropanenitrile':ab,ti) OR ('3 [4 amino 3':ab,ti AND '2 fluoro 4 phenoxyphenyl':ab,ti AND '1h pyrazolo [3, 4 d] pyrimidin 1 yl] alpha [2 methyl 2 [4':ab,ti AND 'oxetan 3 yl':ab,ti AND 'piperazin 1 yl] propylidene] beta oxopiperidine 1 propanenitrile':ab,ti) OR (8:ab,ti AND 4:ab,ti AND 'amino 9':ab,ti AND 2:ab,ti AND 'fluoro 3, 3 dimethyl 6 oxo 10 oxa 8':ab,ti AND 'pyrazolo [3, 4 d] pyrimidina 2':ab,ti AND piperazina7:ab,ti AND '1, 3':ab,ti AND 'piperidina 1':ab,ti AND 3:ab,ti AND 'oxetana 9':ab,ti AND '1, 4':ab,ti AND ', 11':ab,ti AND 1:ab,ti AND 'dibenzenaundecaphan 4 ene 5 carbonitrile':ab,ti) OR 'prn 1008':ab,ti OR prn1008:ab,ti OR 'sar 444671':ab,ti OR sar444671:ab,ti | 62 |
| #39 | 'rilzabrutinib'/exp | 126 |
| #38 | #36 OR #37 | 98 |
| #37 | remibrutinib:ab,ti OR 'lou 064':ab,ti OR lou064:ab,ti OR ('n [3 [6 amino 5 [2':ab,ti AND 'n methyl 2 propenamido':ab,ti AND 'ethoxy] 4 pyrimidinyl] 5 fluoro 2 methylphenyl] 4 cyclopropyl 2 fluorobenzamide':ab,ti) OR ('n [3 [6 amino 5 [2':ab,ti AND 'n methylprop 2 enamido':ab,ti AND 'ethoxy] pyrimidin 4 yl] 5 fluoro 2 methylphenyl] 4 cyclopropyl 2 fluorobenzamide':ab,ti) | 50 |
| #36 | 'remibrutinib'/exp | 94 |
| #35 | #33 OR #34 | 172 |
| #34 | lirentelimab:ab,ti OR 'ak 002':ab,ti OR ak002:ab,ti OR antolimab:ab,ti | 89 |
| #33 | 'lirentelimab'/exp | 161 |
| #32 | #30 OR #31 | 41 |
| #31 | barzolvolimab:ab,ti OR 'cdx 0158':ab,ti OR 'cdx 0159':ab,ti OR cdx0158:ab,ti OR cdx0159:ab,ti OR 'ktn 0158':ab,ti OR ktn0158:ab,ti | 21 |
| #30 | 'barzolvolimab'/exp | 30 |
| #29 | 'azd 1981':ab,ti | 3 |
| #28 | #26 OR #27 | 2854 |
| #27 | benralizumab:ab,ti OR 'biw 8405':ab,ti OR biw8405:ab,ti OR fasenra:ab,ti OR 'khk 4563':ab,ti OR khk4563:ab,ti OR 'medi 563':ab,ti OR medi563:ab,ti | 1415 |
| #26 | 'benralizumab'/exp | 2782 |
| #25 | #23 OR #24 | 7398 |
| #24 | dupilumab:ab,ti OR bat2406:ab,ti OR 'bat 2406':ab,ti OR dupixent:ab,ti OR sar231893:ab,ti OR 'regn 668':ab,ti OR regn668:ab,ti OR 'sar 231893':ab,ti | 4908 |
| #23 | 'dupilumab'/exp | 7211 |
| #22 | #20 OR #21 | 38 |
| #21 | vixarelimab:ab,ti OR 'biib 069':ab,ti OR biib069:ab,ti OR 'kpl 716':ab,ti OR kpl716:ab,ti | 9 |
| #20 | 'vixarelimab'/exp | 33 |
| #19 | #17 OR #18 | 5357 |
| #18 | mepolizumab:ab,ti OR 'bat 2606':ab,ti OR bat2606:ab,ti OR bosatria:ab,ti OR nucala:ab,ti OR 'sb 240563':ab,ti OR sb240563:ab,ti | 2601 |
| #17 | 'mepolizumab'/exp | 5227 |
| #16 | #14 OR #15 | 822 |
| #15 | tezepelumab:ab,ti OR tezspire:ab,ti OR 'tezepelumab ekko':ab,ti OR medi9929:ab,ti OR 'medi 9929':ab,ti OR amg157:ab,ti OR 'amg 157':ab,ti | 346 |
| #14 | 'tezepelumab'/exp | 797 |
| #13 | #11 OR #12 | 84 |
| #12 | quilizumab:ab,ti OR 'memp 1972a':ab,ti OR memp1972a:ab,ti OR 'mep 1972a':ab,ti OR mep1972a:ab,ti OR 'rg 7449':ab,ti OR rg7449:ab,ti | 16 |
| #11 | 'quilizumab'/exp | 83 |
| #10 | 'ub 221':ab,ti | 4 |
| #9 | #7 OR #8 | 303 |
| #8 | ligelizumab:ab,ti OR 'qge 031':ab,ti OR qge031:ab,ti OR 'gbr 310':ab,ti | 120 |
| #7 | 'ligelizumab'/exp | 296 |
| #6 | #4 OR #5 | 12401 |
| #5 | omalizumab:ab,ti OR 'fb 317':ab,ti OR fb317:ab,ti OR 'gbr 310':ab,ti OR gbr310:ab,ti OR 'hu 901':ab,ti OR hu901:ab,ti OR 'monoclonal antibody e 25':ab,ti OR 'monoclonal antibody e25':ab,ti OR olizumab:ab,ti OR 'rg 3648':ab,ti OR rg3648:ab,ti OR rhumab:ab,ti OR 'rhumab e25':ab,ti OR 'sti 004':ab,ti OR sti004:ab,ti OR 'syn 008':ab,ti OR syn008:ab,ti OR xolair:ab,ti | 6981 |
| #4 | 'omalizumab'/exp | 12003 |
| #3 | #1 OR #2 | 210158 |
| #2 | urticaria:ab,ti OR hives:ab,ti OR wheal*:ab,ti OR 'urticarial disease':ab,ti OR 'urticarial disorder':ab,ti OR 'chronic urticaria':ab,ti OR 'chronic spontaneous urticaria':ab,ti OR 'chronic idiopathic urticaria':ab,ti OR 'chronic idiopathic/spontaneous urticaria':ab,ti OR 'chronic spontaneous/idiopathic urticaria':ab,ti OR 'refractory urticaria':ab,ti OR 'antihistamin* resistant chronic urticaria':ab,ti OR cu:ab,ti OR csu:ab,ti OR ciu:ab,ti | 180519 |
| #1 | 'urticaria'/exp | 56164 |

**eTable 1.** Search Strategy from Cochrane Library. (continued)

| **Search** | **Query** | **Results** | |
| --- | --- | --- | --- |
| #1 | MeSH descriptor: [Chronic Urticaria] explode all trees | | 113 |
| #2 | (Urticaria):ti,ab,kw OR (Hive*):ti,ab,kw OR (Wheal*):ti,ab,kw OR (chronic Urticaria):ti,ab,kw OR (chronic spontaneous urticaria):ti,ab,kw | | 4615 |
| #3 | (chronic idiopathic urticaria):ti,ab,kw OR (Autoimmune Urticaria*):ti,ab,kw OR (refractory urticaria):ti,ab,kw OR (antihistamin* resistant chronic urticaria):ti,ab,kw OR (CU):ti,ab,kw | | 1947 |
| #4 | (CSU):ti,ab,kw OR (CIU):ti,ab,kw | | 489 |
| #5 | #1 OR #2 OR #3 OR #4 | | 5954 |
| #6 | MeSH descriptor: [Omalizumab] explode all trees | | 403 |
| #7 | (Xolair):ti,ab,kw | | 199 |
| #8 | #6 OR #7 | | 521 |
| #9 | (ligelizumab):ti,ab,kw | | 71 |
| #10 | (UB-221):ti,ab,kw | | 3 |
| #11 | (quilizumab):ti,ab,kw | | 8 |
| #12 | (tezepelumab):ti,ab,kw | | 185 |
| #13 | (mepolizumab):ti,ab,kw | | 435 |
| #14 | (Vixarelimab):ti,ab,kw | | 4 |
| #15 | (dupilumab):ti,ab,kw | | 1114 |
| #16 | (benralizumab):ti,ab,kw | | 288 |
| #17 | (AZD 1981):ti,ab,kw | | 2 |
| #18 | (barzolvolimab):ti,ab,kw | | 5 |
| #19 | (lirentelimab):ti,ab,kw | | 24 |
| #20 | (remibrutinib):ti,ab,kw | | 39 |
| #21 | (rilzabrutinib):ti,ab,kw | | 18 |
| #22 | (fenebrutinib):ti,ab,kw | | 30 |
| #23 | #8 OR #9 OR #10 OR #11 OR #12 OR #13 OR #14 OR #15 OR #16 OR #17 OR #18 OR #19 OR #20 OR #21 OR #22 | | 2660 |
| #24 | #5 AND #23 in Trials | | 256 |

**eTable 1.** Search Strategy from Web of Science. (continued)

| **Search** | **Query** | **Results** |
| --- | --- | --- |
| 1 | TS=(Urticaria) and Preprint Citation Index (Exclude – Database) | 43503 |
| 2 | ((((((((((((TS=(Urticaria)) OR TI=(urticaria)) OR TI=(Hive*)) OR TI=(Wheal*)) OR TI=(chronic Urticaria)) OR TI=(chronic spontaneous urticaria)) OR TI=(chronic idiopathic urticaria)) OR TI=(Autoimmune Urticaria*)) OR TI=(refractory urticaria)) OR TI=(antihistamin* resistant chronic urticaria)) OR TI=(CU)) OR TI=(CSU)) OR TI=(CIU) and Preprint Citation Index (Exclude – Database) | 252380 |
| 3 | TS=(omalizumab) and Preprint Citation Index (Exclude – Database) | 7818 |
| 4 | TS=(Omalizumab) and Preprint Citation Index (Exclude – Database) | 7818 |
| 5 | ((TS=(Omalizumab)) OR TI=(omalizumab)) OR TI=(Xolair) and Preprint Citation Index (Exclude – Database) | 7837 |
| 6 | TS=(Ligelizumab) and Preprint Citation Index (Exclude – Database) | 160 |
| 7 | TS=(ligelizumab) and Preprint Citation Index (Exclude – Database) | 160 |
| 8 | (((TS=(Ligelizumab)) OR TI=(ligelizumab)) OR TI=(QGE-031)) OR TI=(QGE031) and Preprint Citation Index (Exclude – Database) | 163 |
| 9 | TS=(UB-221) and Preprint Citation Index (Exclude – Database) | 5 |
| 10 | (TS=(UB-221)) OR TI=(UB-221) and Preprint Citation Index (Exclude – Database) | 5 |
| 11 | (TS=(Quilizumab)) OR TI=(Quilizumab) and Preprint Citation Index (Exclude – Database) | 22 |
| 12 | (((((((TS=(Tezepelumab)) OR TI=(tezepelumab)) OR TI=(tezepelumab-ekko)) OR TI=(tezspire)) OR TI=(MEDI-9929)) OR TI=(MEDI-19929)) OR TI=(MEDI9929)) OR TI=(AMG-157) and Preprint Citation Index (Exclude – Database) | 430 |
| 13 | (((((TS=(mepolizumab)) OR TI=(mepolizumab)) OR TI=(Bosatria)) OR TI=(SB-240563)) OR TI=(SB240563)) OR TI=(Nucala) and Preprint Citation Index (Exclude – Database) | 3456 |
| 14 | (TS=(Vixarelimab)) OR TI=(Vixarelimab) and Preprint Citation Index (Exclude – Database) | 3 |
| 15 | ((((((TS=(Dupilumab)) OR TI=(Dupilumab)) OR TI=(SAR231893)) OR TI=(SAR-231893)) OR TI=(Dupixent)) OR TI=(REGN668)) OR TI=(REGN-668) and Preprint Citation Index (Exclude – Database) | 5183 |
| 16 | (((((TS=(benralizumab)) OR TI=(benralizumab)) OR TI=(MEDI-563)) OR TI=(MEDI 563)) OR TI=(Fasenra)) OR TI=(BIW-8405) and Preprint Citation Index (Exclude – Database) | 1614 |
| 17 | (TS=(AZD 1981)) OR TI=(AZD 1981) and Preprint Citation Index (Exclude – Database) | 8 |
| 18 | (TS=(barzolvolimab)) OR TI=(barzolvolimab) and Preprint Citation Index (Exclude – Database) | 6 |
| 19 | (TS=(lirentelimab)) OR TI=(lirentelimab) and Preprint Citation Index (Exclude – Database) | 38 |
| 20 | (((TS=(remibrutinib)) OR TI=(remibrutinib)) OR TI=(LOU064)) OR TI=(• N-(3-(6-amino-5-(2-(methyl(prop-2-enoyl)amino)ethoxy)pyrimidin-4-yl)-5-fluoro-2-methylphenyl)-4-cyclopropyl-2-fluorobenzamide) and Preprint Citation Index (Exclude – Database) | 66 |
| 21 | (TS=(rilzabrutinib)) OR TI=(rilzabrutinib) and Preprint Citation Index (Exclude – Database) | 53 |
| 22 | (((TS=(Fenebrutinib)) OR TI=(Fenebrutinib)) OR TI=(• 2-(3'-(hydroxymethyl)-1-methyl-5-((5-(2-methyl-4-(oxetan-3-yl)piperazin-1-yl)pyridin-2-yl)amino)-6-oxo-1,6-dihydro-(3,4'-bipyridin)-2'-yl)-7,7-dimethyl-3,4,7,8-tetrahydro-2H-cyclopenta(4,5)pyrrolo(1,2-a)pyrazin-1(6H)-one)) OR TI=(GDC-0853) and Preprint Citation Index (Exclude – Database) | 99 |
| 23 | #22 OR #21 OR #20 OR #19 OR #18 OR #17 OR #16 OR #15 OR #14 OR #13 OR #12 OR #11 OR #10 OR #8 OR #5 and Preprint Citation Index (Exclude – Database) | 16009 |
| 24 | #22 OR #21 OR #20 OR #19 OR #18 OR #17 OR #16 OR #15 OR #14 OR #13 OR #12 OR #11 OR #10 OR #8 OR #5 and Preprint Citation Index (Exclude – Database) | 16009 |
| 25 | #2 AND #24 and Preprint Citation Index (Exclude – Database) | 2282 |
| 26 | TI=(random* OR placebo* OR trial) and Preprint Citation Index (Exclude – Database) | 1123420 |
| 27 | AB=(random* OR placebo*) and Preprint Citation Index (Exclude – Database) | 3408240 |
| 28 | #27 OR #26 and Preprint Citation Index (Exclude – Database) | 3909322 |
| 29 | #28 AND #25 and Preprint Citation Index (Exclude – Database) | 227 |

**eFigure 1.** PRISMA flowchart for the detailed process of study selection.


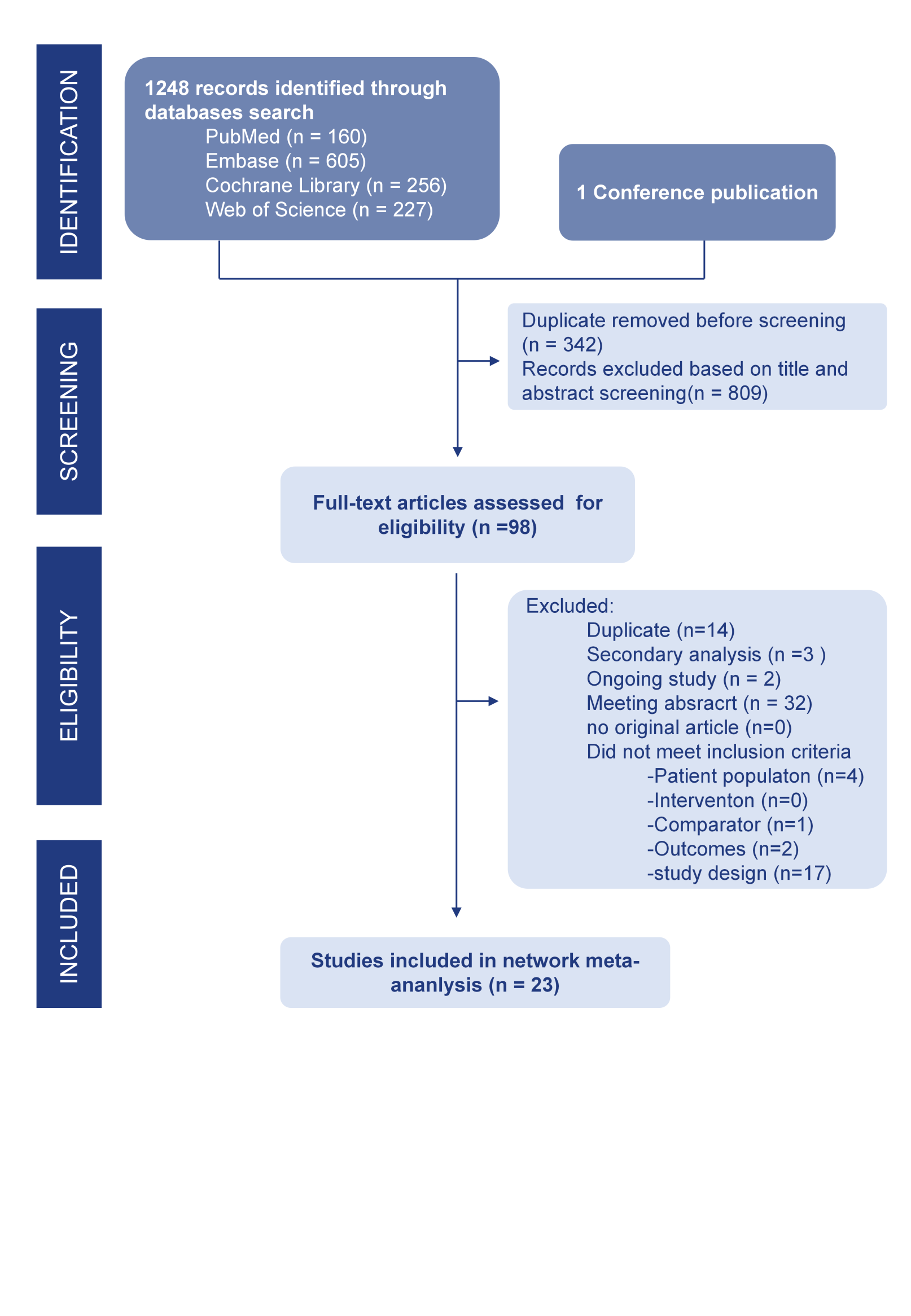


**eFigure 2.** Risk of bias graph

**
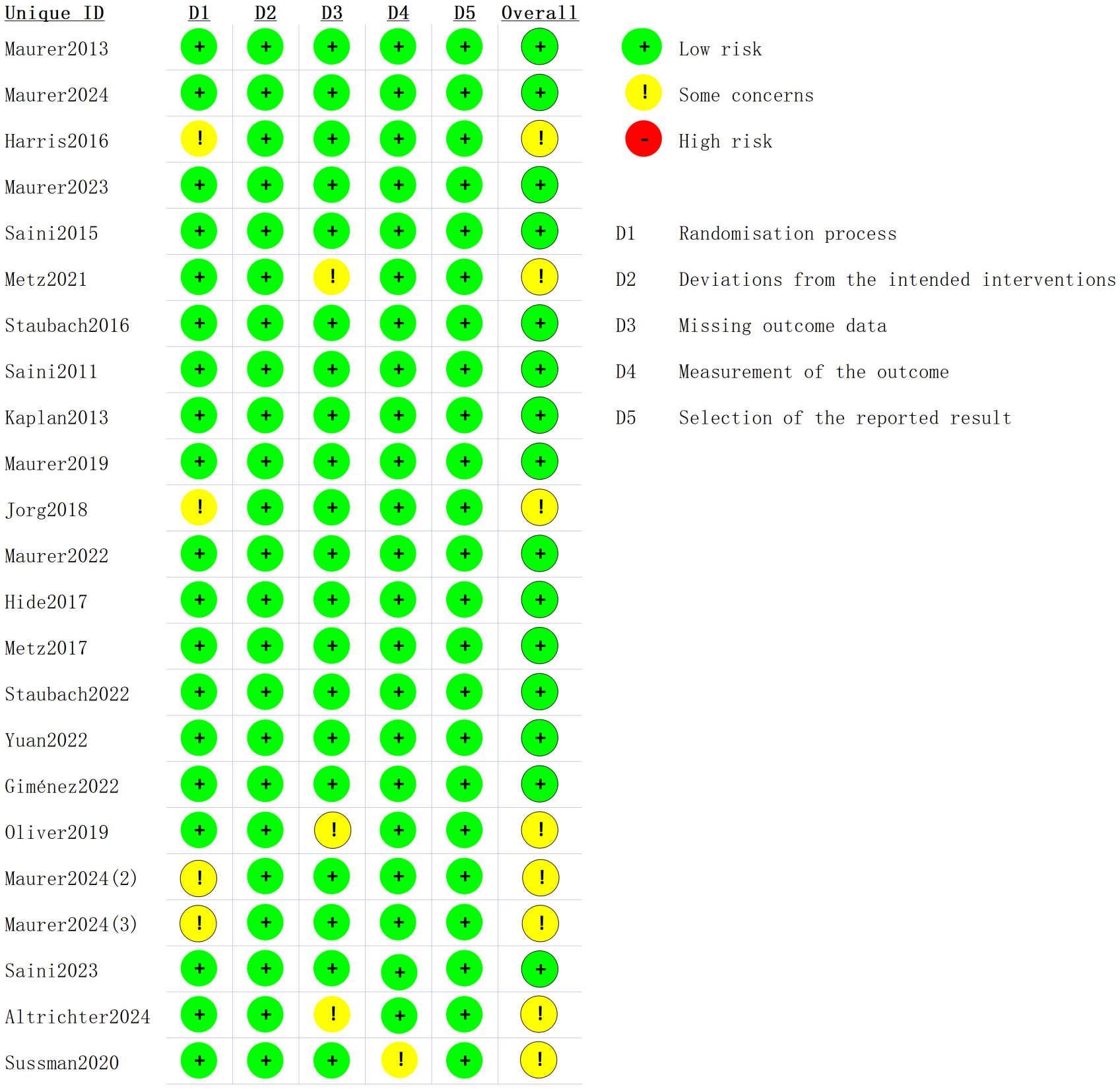
**

**eTable 2.** League table and the Surface Under the Cumulative Ranking (SUCRA): change from baseline in the weekly urticaria activity score (UAS7).

|  | AZD | Ben30mg | Ben60mg | Dup | Fen150mg | Fen200mg | Fen50mg | Lig120mg | Lig240mg | Lig24mg | Lig72mg | Oma150mg | Oma300mg | Oma600mg | Oma75mg | Placebo | Qui | Remi100mg bid | Remi100mg qd | Remi10mg bid | Remi10mg qd | Remi25mg bid | Remi35mg qd | Ril | Tez210mg | Tez420mg |
| --- | --- | --- | --- | --- | --- | --- | --- | --- | --- | --- | --- | --- | --- | --- | --- | --- | --- | --- | --- | --- | --- | --- | --- | --- | --- | --- |
| AZD | AZD | -6.79 (-17.64, 4.71) | -9.11 (-20.17, 2) | -10.1 (-20.72, 1.16) | -10.58 (-22.49, 2.52) | -13.03 (-23.92, -1.43) | -4.57 (-16.7, 7.84) | -14.87 (-24.97, -4.37) | -12.87 (-27.21, 0.79) | -11.92 (-24.46, 0.91) | -14.19 (-24.31, -3.55) | -11.9 (-21.8, -1.12) | -14.84 (-24.85, -4.41) | -11.66 (-22.71, 0.42) | -8 (-18.31, 2.54) | -4.85 (-14.73, 5.57) | -9.98 (-24.04, 4.57) | -11.9 (-22.84, 0.15) | -9.55 (-20.87, 1.69) | -12.18 (-22.95, -1.04) | -12.78 (-23.49, -0.91) | -12.44 (-22.56, -1.67) | -12.34 (-23.46, -1.1) | -11.4 (-23.06, 0.02) | -9.8 (-20.82, 1.82) | -9.57 (-20.66, 2.38) |
| Ben30mg | 6.79 (-4.71, 17.64) | Ben30mg | -2.34 (-7.01, 2.54) | -3.16 (-9.5, 3.26) | -3.74 (-11.84, 4.44) | -6.24 (-13.04, 1) | 2.36 (-6.09, 10.33) | -8.02 (-13.35, -2.42) | -6.36 (-17.58, 5.19) | -5.25 (-13.58, 3.48) | -7.42 (-12.77, -1.88) | -5.07 (-10.32, 0.33) | -8.04 (-13.15, -2.72) | -4.9 (-12.27, 2.86) | -1.28 (-6.86, 4.46) | 1.95 (-3.04, 7.17) | -3.35 (-14.25, 7.23) | -5.17 (-12.46, 2.15) | -2.98 (-9.88, 4.37) | -5.47 (-12.37, 1.63) | -5.77 (-12.75, 1.27) | -5.71 (-11.07, -0.34) | -5.71 (-12.52, 1.41) | -4.71 (-12.35, 2.94) | -3.11 (-9.91, 4.21) | -2.86 (-9.85, 4.42) |
| Ben60mg | 9.11 (-2, 20.17) | 2.34 (-2.54, 7.01) | Ben60mg | -0.85 (-7.17, 5.52) | -1.44 (-9.53, 6.86) | -3.91 (-10.72, 3.21) | 4.63 (-3.74, 12.51) | -5.68 (-11, -0.44) | -4.08 (-15.35, 7.26) | -2.91 (-11.16, 5.78) | -5.1 (-10.36, 0.27) | -2.73 (-7.98, 2.57) | -5.71 (-10.8, -0.57) | -2.57 (-9.79, 5.14) | 1.04 (-4.39, 6.59) | 4.29 (-0.67, 9.32) | -0.92 (-11.98, 9.56) | -2.91 (-9.89, 4.28) | -0.47 (-7.64, 6.56) | -3.12 (-10.06, 3.92) | -3.4 (-10.58, 3.47) | -3.34 (-8.72, 1.98) | -3.31 (-10.37, 3.64) | -2.34 (-10.01, 5.13) | -0.76 (-7.53, 6.34) | -0.42 (-7.41, 6.52) |
| Dup | 10.1 (-1.16, 20.72) | 3.16 (-3.26, 9.5) | 0.85 (-5.52, 7.17) | Dup | -0.54 (-8.57, 7.22) | -2.82 (-9.84, 3.25) | 5.37 (-3.01, 13.1) | -4.8 (-9, -0.56) | -3.38 (-14.2, 7.57) | -1.91 (-10.04, 5.69) | -4.15 (-8.31, 0.06) | -1.8 (-5.93, 2.62) | -4.81 (-8.78, -0.7) | -1.84 (-8.43, 5.36) | 1.87 (-2.58, 6.61) | 5.22 (1.22, 9.13) | -0.13 (-10.69, 9.84) | -1.96 (-8.1, 4.7) | 0.36 (-5.74, 6.55) | -2.16 (-8.29, 4.04) | -2.55 (-9.4, 3.67) | -2.42 (-6.73, 2.01) | -2.5 (-8.99, 3.71) | -1.49 (-8.13, 5.2) | 0.27 (-6.49, 6.61) | 0.5 (-6.1, 6.87) |
| Fen150mg | 10.58 (-2.52, 22.49) | 3.74 (-4.44, 11.84) | 1.44 (-6.86, 9.53) | 0.54 (-7.22, 8.57) | Fen150mg | -2.29 (-8.92, 4.14) | 6.05 (-1.11, 13.01) | -4.21 (-11.02, 2.36) | -2.62 (-14.51, 9.26) | -1.51 (-11.17, 7.89) | -3.54 (-10.4, 2.97) | -1.23 (-8.03, 5.35) | -4.22 (-10.98, 2.18) | -1.23 (-10.28, 7.45) | 2.44 (-4.54, 9.48) | 5.8 (-0.75, 12.1) | 0.34 (-10.94, 12.16) | -1.42 (-9.49, 6.84) | 0.96 (-7.2, 8.87) | -1.67 (-9.61, 6.37) | -1.98 (-10.11, 6.12) | -1.87 (-8.72, 4.84) | -1.87 (-10.14, 5.89) | -0.94 (-9.32, 7.13) | 0.76 (-7.3, 9.03) | 1.01 (-7.08, 9) |
| Fen200mg | 13.03 (1.43, 23.92) | 6.24 (-1, 13.04) | 3.91 (-3.21, 10.72) | 2.82 (-3.25, 9.84) | 2.29 (-4.14, 8.92) | Fen200mg | 8.25 (1.69, 15.06) | -1.9 (-7.17, 3.34) | -0.04 (-11, 10.5) | 0.94 (-7.38, 9.05) | -1.24 (-6.53, 4.06) | 1.08 (-4.07, 6.32) | -1.8 (-7.03, 3.21) | 1.17 (-6.05, 8.36) | 4.82 (-0.57, 10.39) | 8.23 (3.15, 12.99) | 2.87 (-8.17, 13.67) | 1.01 (-6.13, 7.99) | 3.46 (-3.59, 10.16) | 0.8 (-5.93, 7.68) | 0.49 (-6.31, 7.42) | 0.56 (-4.82, 5.7) | 0.49 (-6.47, 7.3) | 1.43 (-5.91, 8.53) | 3.28 (-3.59, 10.11) | 3.35 (-3.76, 10.28) |
| Fen50mg | 4.57 (-7.84, 16.7) | -2.36 (-10.33, 6.09) | -4.63 (-12.51, 3.74) | -5.37 (-13.1, 3.01) | -6.05 (-13.01, 1.11) | -8.25 (-15.06, -1.69) | Fen50mg | -10.23 (-16.89, -3.27) | -8.38 (-20.52, 3.06) | -7.49 (-16.67, 1.95) | -9.57 (-16.37, -2.59) | -7.18 (-14.1, -0.22) | -10.2 (-16.87, -3.34) | -7.13 (-15.8, 1.85) | -3.48 (-11.15, 3.76) | -0.15 (-6.65, 6.48) | -5.58 (-16.99, 6.55) | -7.3 (-15.33, 0.87) | -5.02 (-13.12, 2.98) | -7.71 (-15.49, 0.46) | -7.94 (-15.92, 0.2) | -7.83 (-14.8, -0.95) | -8 (-15.83, 0.33) | -6.98 (-15.23, 1.55) | -5.11 (-13.22, 2.99) | -4.83 (-12.83, 3.29) |
| Lig120mg | 14.87 (4.37, 24.97) | 8.02 (2.42, 13.35) | 5.68 (0.44, 11) | 4.8 (0.56, 9) | 4.21 (-2.36, 11.02) | 1.9 (-3.34, 7.17) | 10.23 (3.27, 16.89) | Lig120mg | 1.68 (-8.72, 12.51) | 2.94 (-4.06, 9.57) | 0.63 (-1.21, 2.46) | 2.97 (0.65, 5.42) | 0.01 (-1.77, 1.69) | 3 (-2.95, 9.17) | 6.73 (3.85, 9.65) | 10.01 (8.18, 11.89) | 4.84 (-5.27, 14.38) | 2.79 (-2.69, 8.35) | 5.2 (-0.1, 10.59) | 2.66 (-2.64, 7.8) | 2.31 (-3.52, 7.65) | 2.39 (-0.4, 4.99) | 2.33 (-2.83, 7.61) | 3.26 (-2.46, 9.03) | 5.04 (-0.04, 10.21) | 5.21 (0.24, 10.23) |
| Lig240mg | 12.87 (-0.79, 27.21) | 6.36 (-5.19, 17.58) | 4.08 (-7.26, 15.35) | 3.38 (-7.57, 14.2) | 2.62 (-9.26, 14.51) | 0.04 (-10.5, 11) | 8.38 (-3.06, 20.52) | -1.68 (-12.51, 8.72) | Lig240mg | 1.36 (-10.51, 12.51) | -1.03 (-11.85, 9.25) | 1.31 (-9.62, 11.64) | -1.68 (-12.58, 8.47) | 1.49 (-10.64, 13.25) | 5.14 (-6.34, 15.74) | 8.35 (-2.13, 18.46) | 2.86 (-10.62, 17) | 1.15 (-11.04, 12.26) | 3.46 (-8.1, 14.63) | 0.95 (-10.73, 12.13) | 0.69 (-10.97, 11.75) | 0.64 (-10.34, 10.97) | 0.83 (-10.93, 11.8) | 1.61 (-9.92, 13.03) | 3.28 (-7.81, 14.68) | 3.37 (-7.78, 14.88) |
| Lig24mg | 11.92 (-0.91, 24.46) | 5.25 (-3.48, 13.58) | 2.91 (-5.78, 11.16) | 1.91 (-5.69, 10.04) | 1.51 (-7.89, 11.17) | -0.94 (-9.05, 7.38) | 7.49 (-1.95, 16.67) | -2.94 (-9.57, 4.06) | -1.36 (-12.51, 10.51) | Lig24mg | -2.32 (-8.95, 4.81) | 0.05 (-7.04, 7.38) | -2.84 (-9.63, 4.1) | 0.16 (-8.41, 9.13) | 3.89 (-3.1, 11.21) | 7.22 (0.38, 14.06) | 2.04 (-10.78, 13.57) | -0.04 (-8.49, 9.12) | 2.51 (-6.19, 10.69) | -0.07 (-8.38, 7.78) | -0.48 (-8.89, 7.99) | -0.46 (-7.6, 6.82) | -0.47 (-8.74, 7.67) | 0.43 (-8.22, 9.03) | 2.31 (-5.92, 11.11) | 2.34 (-5.89, 11) |
| Lig72mg | 14.19 (3.55, 24.31) | 7.42 (1.88, 12.77) | 5.1 (-0.27, 10.36) | 4.15 (-0.06, 8.31) | 3.54 (-2.97, 10.4) | 1.24 (-4.06, 6.53) | 9.57 (2.59, 16.37) | -0.63 (-2.46, 1.21) | 1.03 (-9.25, 11.85) | 2.32 (-4.81, 8.95) | Lig72mg | 2.35 (-0.02, 4.79) | -0.62 (-2.41, 1.07) | 2.34 (-3.44, 8.52) | 6.08 (3.27, 9.04) | 9.37 (7.54, 11.25) | 4.2 (-5.88, 13.73) | 2.17 (-3.24, 7.74) | 4.59 (-0.66, 9.84) | 2.02 (-3.23, 7.22) | 1.68 (-3.68, 6.96) | 1.75 (-1.02, 4.36) | 1.71 (-3.44, 6.98) | 2.68 (-3.14, 8.36) | 4.38 (-0.66, 9.55) | 4.52 (-0.39, 9.7) |
| Oma150mg | 11.9 (1.12, 21.8) | 5.07 (-0.33, 10.32) | 2.73 (-2.57, 7.98) | 1.8 (-2.62, 5.93) | 1.23 (-5.35, 8.03) | -1.08 (-6.32, 4.07) | 7.18 (0.22, 14.1) | -2.97 (-5.42, -0.65) | -1.31 (-11.64, 9.62) | -0.05 (-7.38, 7.04) | -2.35 (-4.79, 0.02) | Oma150mg | -2.98 (-4.82, -1.25) | 0.03 (-5.95, 6.16) | 3.72 (1.09, 6.43) | 7.02 (5.18, 8.8) | 1.84 (-8.26, 11.35) | -0.14 (-5.65, 5.41) | 2.22 (-3.09, 7.44) | -0.31 (-5.54, 4.81) | -0.73 (-5.98, 4.56) | -0.6 (-3.43, 1.94) | -0.61 (-5.79, 4.55) | 0.33 (-5.5, 6) | 2.07 (-3.07, 7.17) | 2.2 (-2.73, 7.26) |
| Oma300mg | 14.84 (4.41, 24.85) | 8.04 (2.72, 13.15) | 5.71 (0.57, 10.8) | 4.81 (0.7, 8.78) | 4.22 (-2.18, 10.98) | 1.8 (-3.21, 7.03) | 10.2 (3.34, 16.87) | -0.01 (-1.69, 1.77) | 1.68 (-8.47, 12.58) | 2.84 (-4.1, 9.63) | 0.62 (-1.07, 2.41) | 2.98 (1.25, 4.82) | Oma300mg | 3.02 (-2.66, 8.96) | 6.73 (4.21, 9.3) | 9.99 (8.8, 11.31) | 4.84 (-5.11, 14.3) | 2.77 (-2.44, 8.2) | 5.19 (0.17, 10.36) | 2.61 (-2.38, 7.65) | 2.29 (-3.14, 7.44) | 2.4 (-0.01, 4.65) | 2.36 (-2.59, 7.43) | 3.33 (-2.32, 8.86) | 5.03 (0.23, 10.02) | 5.19 (0.45, 10.08) |
| Oma600mg | 11.66 (-0.42, 22.71) | 4.9 (-2.86, 12.27) | 2.57 (-5.14, 9.79) | 1.84 (-5.36, 8.43) | 1.23 (-7.45, 10.28) | -1.17 (-8.36, 6.05) | 7.13 (-1.85, 15.8) | -3 (-9.17, 2.95) | -1.49 (-13.25, 10.64) | -0.16 (-9.13, 8.41) | -2.34 (-8.52, 3.44) | -0.03 (-6.16, 5.95) | -3.02 (-8.96, 2.66) | Oma600mg | 3.66 (-2.38, 9.74) | 7 (1.1, 12.77) | 1.98 (-9.51, 12.05) | -0.21 (-8.12, 7.82) | 2.11 (-5.68, 9.68) | -0.5 (-8.03, 6.93) | -0.79 (-8.82, 6.75) | -0.66 (-6.99, 5.41) | -0.72 (-8.29, 6.62) | 0.2 (-7.93, 8.26) | 2.03 (-5.44, 9.71) | 2.23 (-5.52, 9.84) |
| Oma75mg | 8 (-2.54, 18.31) | 1.28 (-4.46, 6.86) | -1.04 (-6.59, 4.39) | -1.87 (-6.61, 2.58) | -2.44 (-9.48, 4.54) | -4.82 (-10.39, 0.57) | 3.48 (-3.76, 11.15) | -6.73 (-9.65, -3.85) | -5.14 (-15.74, 6.34) | -3.89 (-11.21, 3.1) | -6.08 (-9.04, -3.27) | -3.72 (-6.43, -1.09) | -6.73 (-9.3, -4.21) | -3.66 (-9.74, 2.38) | Oma75mg | 3.3 (0.76, 5.8) | -1.93 (-12, 7.65) | -3.91 (-9.7, 1.88) | -1.59 (-7.07, 3.95) | -4.09 (-9.61, 1.31) | -4.47 (-10.03, 1.07) | -4.34 (-7.64, -1.32) | -4.4 (-9.96, 1.07) | -3.42 (-9.49, 2.53) | -1.69 (-7.16, 3.72) | -1.47 (-6.8, 3.79) |
| Placebo | 4.85 (-5.57, 14.73) | -1.95 (-7.17, 3.04) | -4.29 (-9.32, 0.67) | -5.22 (-9.13, -1.22) | -5.8 (-12.1, 0.75) | -8.23 (-12.99, -3.15) | 0.15 (-6.48, 6.65) | -10.01 (-11.89, -8.18) | -8.35 (-18.46, 2.13) | -7.22 (-14.06, -0.38) | -9.37 (-11.25, -7.54) | -7.02 (-8.8, -5.18) | -9.99 (-11.31, -8.8) | -7 (-12.77, -1.1) | -3.3 (-5.8, -0.76) | Placebo | -5.17 (-15, 4.2) | -7.22 (-12.32, -2.04) | -4.82 (-9.74, 0.09) | -7.3 (-12.27, -2.57) | -7.71 (-12.83, -2.8) | -7.63 (-9.73, -5.72) | -7.68 (-12.49, -2.72) | -6.69 (-12.18, -1.33) | -4.98 (-9.79, -0.02) | -4.86 (-9.6, 0.01) |
| Qui | 9.98 (-4.57, 24.04) | 3.35 (-7.23, 14.25) | 0.92 (-9.56, 11.98) | 0.13 (-9.84, 10.69) | -0.34 (-12.16, 10.94) | -2.87 (-13.67, 8.17) | 5.58 (-6.55, 16.99) | -4.84 (-14.38, 5.27) | -2.86 (-17, 10.62) | -2.04 (-13.57, 10.78) | -4.2 (-13.73, 5.88) | -1.84 (-11.35, 8.26) | -4.84 (-14.3, 5.11) | -1.98 (-12.05, 9.51) | 1.93 (-7.65, 12) | 5.17 (-4.2, 15) | Qui | -2.01 (-12.19, 9.15) | 0.35 (-10.01, 11.15) | -2.31 (-12.83, 8.78) | -2.58 (-13.17, 8.53) | -2.49 (-11.99, 7.6) | -2.51 (-12.87, 8.48) | -1.47 (-12.63, 9.55) | 0.2 (-10.12, 11.36) | 0.41 (-10.15, 11.25) |
| Remi100mg bid | 11.9 (-0.15, 22.84) | 5.17 (-2.15, 12.46) | 2.91 (-4.28, 9.89) | 1.96 (-4.7, 8.1) | 1.42 (-6.84, 9.49) | -1.01 (-7.99, 6.13) | 7.3 (-0.87, 15.33) | -2.79 (-8.35, 2.69) | -1.15 (-12.26, 11.04) | 0.04 (-9.12, 8.49) | -2.17 (-7.74, 3.24) | 0.14 (-5.41, 5.65) | -2.77 (-8.2, 2.44) | 0.21 (-7.82, 8.12) | 3.91 (-1.88, 9.7) | 7.22 (2.04, 12.32) | 2.01 (-9.15, 12.19) | Remi100mg bid | 2.39 (-3.31, 7.92) | -0.14 (-6.26, 5.33) | -0.62 (-6.24, 5.11) | -0.43 (-5.66, 4.57) | -0.57 (-6.28, 5.04) | 0.52 (-7, 8.06) | 2.12 (-4.89, 9.16) | 2.34 (-4.47, 9.11) |
| Remi100mg qd | 9.55 (-1.69, 20.87) | 2.98 (-4.37, 9.88) | 0.47 (-6.56, 7.64) | -0.36 (-6.55, 5.74) | -0.96 (-8.87, 7.2) | -3.46 (-10.16, 3.59) | 5.02 (-2.98, 13.12) | -5.2 (-10.59, 0.1) | -3.46 (-14.63, 8.1) | -2.51 (-10.69, 6.19) | -4.59 (-9.84, 0.66) | -2.22 (-7.44, 3.09) | -5.19 (-10.36, -0.17) | -2.11 (-9.68, 5.68) | 1.59 (-3.95, 7.07) | 4.82 (-0.09, 9.74) | -0.35 (-11.15, 10.01) | -2.39 (-7.92, 3.31) | Remi100mg qd | -2.52 (-8.15, 2.9) | -3.03 (-8.32, 2.64) | -2.84 (-7.69, 2.11) | -2.93 (-8.18, 2.59) | -1.89 (-9.19, 5.51) | -0.27 (-7.08, 6.94) | -0.03 (-6.72, 6.88) |
| Remi10mg bid | 12.18 (1.04, 22.95) | 5.47 (-1.63, 12.37) | 3.12 (-3.92, 10.06) | 2.16 (-4.04, 8.29) | 1.67 (-6.37, 9.61) | -0.8 (-7.68, 5.93) | 7.71 (-0.46, 15.49) | -2.66 (-7.8, 2.64) | -0.95 (-12.13, 10.73) | 0.07 (-7.78, 8.38) | -2.02 (-7.22, 3.23) | 0.31 (-4.81, 5.54) | -2.61 (-7.65, 2.38) | 0.5 (-6.93, 8.03) | 4.09 (-1.31, 9.61) | 7.3 (2.57, 12.27) | 2.31 (-8.78, 12.83) | 0.14 (-5.33, 6.26) | 2.52 (-2.9, 8.15) | Remi10mg bid | -0.39 (-5.84, 5.11) | -0.31 (-5.14, 4.56) | -0.23 (-5.66, 5.1) | 0.68 (-6.5, 7.73) | 2.35 (-4.35, 9.36) | 2.51 (-4.13, 9.46) |
| Remi10mg qd | 12.78 (0.91, 23.49) | 5.77 (-1.27, 12.75) | 3.4 (-3.47, 10.58) | 2.55 (-3.67, 9.4) | 1.98 (-6.12, 10.11) | -0.49 (-7.42, 6.31) | 7.94 (-0.2, 15.92) | -2.31 (-7.65, 3.52) | -0.69 (-11.75, 10.97) | 0.48 (-7.99, 8.89) | -1.68 (-6.96, 3.68) | 0.73 (-4.56, 5.98) | -2.29 (-7.44, 3.14) | 0.79 (-6.75, 8.82) | 4.47 (-1.07, 10.03) | 7.71 (2.8, 12.83) | 2.58 (-8.53, 13.17) | 0.62 (-5.11, 6.24) | 3.03 (-2.64, 8.32) | 0.39 (-5.11, 5.84) | Remi10mg qd | 0.09 (-4.88, 5.13) | 0.1 (-5.44, 5.51) | 1.02 (-6.28, 8.27) | 2.78 (-4.07, 9.76) | 2.99 (-3.74, 9.73) |
| Remi25mg bid | 12.44 (1.67, 22.56) | 5.71 (0.34, 11.07) | 3.34 (-1.98, 8.72) | 2.42 (-2.01, 6.73) | 1.87 (-4.84, 8.72) | -0.56 (-5.7, 4.82) | 7.83 (0.95, 14.8) | -2.39 (-4.99, 0.4) | -0.64 (-10.97, 10.34) | 0.46 (-6.82, 7.6) | -1.75 (-4.36, 1.02) | 0.6 (-1.94, 3.43) | -2.4 (-4.65, 0.01) | 0.66 (-5.41, 6.99) | 4.34 (1.32, 7.64) | 7.63 (5.72, 9.73) | 2.49 (-7.6, 11.99) | 0.43 (-4.57, 5.66) | 2.84 (-2.11, 7.69) | 0.31 (-4.56, 5.14) | -0.09 (-5.13, 4.88) | Remi25mg bid | -0.02 (-4.83, 4.87) | 0.95 (-4.85, 6.76) | 2.61 (-2.44, 8.04) | 2.82 (-2.28, 8.17) |
| Remi35mg qd | 12.34 (1.1, 23.46) | 5.71 (-1.41, 12.52) | 3.31 (-3.64, 10.37) | 2.5 (-3.71, 8.99) | 1.87 (-5.89, 10.14) | -0.49 (-7.3, 6.47) | 8 (-0.33, 15.83) | -2.33 (-7.61, 2.83) | -0.83 (-11.8, 10.93) | 0.47 (-7.67, 8.74) | -1.71 (-6.98, 3.44) | 0.61 (-4.55, 5.79) | -2.36 (-7.43, 2.59) | 0.72 (-6.62, 8.29) | 4.4 (-1.07, 9.96) | 7.68 (2.72, 12.49) | 2.51 (-8.48, 12.87) | 0.57 (-5.04, 6.28) | 2.93 (-2.59, 8.18) | 0.23 (-5.1, 5.66) | -0.1 (-5.51, 5.44) | 0.02 (-4.87, 4.83) | Remi35mg qd | 1.04 (-6.45, 8.26) | 2.76 (-4.19, 9.7) | 2.91 (-3.78, 9.61) |
| Ril | 11.4 (-0.02, 23.06) | 4.71 (-2.94, 12.35) | 2.34 (-5.13, 10.01) | 1.49 (-5.2, 8.13) | 0.94 (-7.13, 9.32) | -1.43 (-8.53, 5.91) | 6.98 (-1.55, 15.23) | -3.26 (-9.03, 2.46) | -1.61 (-13.03, 9.92) | -0.43 (-9.03, 8.22) | -2.68 (-8.36, 3.14) | -0.33 (-6, 5.5) | -3.33 (-8.86, 2.32) | -0.2 (-8.26, 7.93) | 3.42 (-2.53, 9.49) | 6.69 (1.33, 12.18) | 1.47 (-9.55, 12.63) | -0.52 (-8.06, 7) | 1.89 (-5.51, 9.19) | -0.68 (-7.73, 6.5) | -1.02 (-8.27, 6.28) | -0.95 (-6.76, 4.85) | -1.04 (-8.26, 6.45) | Ril | 1.72 (-5.54, 9.09) | 1.95 (-5.25, 9.12) |
| Tez210mg | 9.8 (-1.82, 20.82) | 3.11 (-4.21, 9.91) | 0.76 (-6.34, 7.53) | -0.27 (-6.61, 6.49) | -0.76 (-9.03, 7.3) | -3.28 (-10.11, 3.59) | 5.11 (-2.99, 13.22) | -5.04 (-10.21, 0.04) | -3.28 (-14.68, 7.81) | -2.31 (-11.11, 5.92) | -4.38 (-9.55, 0.66) | -2.07 (-7.17, 3.07) | -5.03 (-10.02, -0.23) | -2.03 (-9.71, 5.44) | 1.69 (-3.72, 7.16) | 4.98 (0.02, 9.79) | -0.2 (-11.36, 10.12) | -2.12 (-9.16, 4.89) | 0.27 (-6.94, 7.08) | -2.35 (-9.36, 4.35) | -2.78 (-9.76, 4.07) | -2.61 (-8.04, 2.44) | -2.76 (-9.7, 4.19) | -1.72 (-9.09, 5.54) | Tez210mg | 0.18 (-5.46, 5.93) |
| Tez420mg | 9.57 (-2.38, 20.66) | 2.86 (-4.42, 9.85) | 0.42 (-6.52, 7.41) | -0.5 (-6.87, 6.1) | -1.01 (-9, 7.08) | -3.35 (-10.28, 3.76) | 4.83 (-3.29, 12.83) | -5.21 (-10.23, -0.24) | -3.37 (-14.88, 7.78) | -2.34 (-11, 5.89) | -4.52 (-9.7, 0.39) | -2.2 (-7.26, 2.73) | -5.19 (-10.08, -0.45) | -2.23 (-9.84, 5.52) | 1.47 (-3.79, 6.8) | 4.86 (-0.01, 9.6) | -0.41 (-11.25, 10.15) | -2.34 (-9.11, 4.47) | 0.03 (-6.88, 6.72) | -2.51 (-9.46, 4.13) | -2.99 (-9.73, 3.74) | -2.82 (-8.17, 2.28) | -2.91 (-9.61, 3.78) | -1.95 (-9.12, 5.25) | -0.18 (-5.93, 5.46) | Tez420mg |
| SUCRA | 0.0501 | 0.198 | 0.36 | 0.4184 | 0.4837 | 0.6885 | 0.118 | 0.8819 | 0.6512 | 0.593 | 0.8173 | 0.5843 | 0.8857 | 0.5782 | 0.252 | 0.0778 | 0.4566 | 0.5981 | 0.385 | 0.6196 | 0.6544 | 0.6497 | 0.6477 | 0.5573 | 0.4055 | 0.3879 |

For change from baseline in the weekly urticaria activity score (UAS7), Network meta-analysis results are expressed as MD (95% CI). MDs less than 0 indicate that the treatment specified in the row is more effective than that specified in the column. The bottom row contains the Surface Under the Cumulative Ranking (SUCRA) value for the column-defining treatment. Both in efficacy and safety, a higher score on the lower surface under the cumulative ranking curve (SUCRA) indicate better efficacy and higher safety.

**eTable 3.** GRADE Assessment by CINeMA: change from baseline in the weekly urticaria activity score (UAS7)

| **Treatment  comparison  (reference: placebo)** | **Number of studies** | **Within-study bias** | **Reporting bias** | **Indirectness** | **Imprecision** | **Heterogeneity** | **Incoherence** | **Confidence rating** |
| --- | --- | --- | --- | --- | --- | --- | --- | --- |
| AZD1981 | 1 | No concerns | Low risk | No concerns | Major concerns | No concerns | No concerns | Low |
| Benralizumab(30mg) | 1 | Some concerns | Low risk | No concerns | Major concerns | No concerns | No concerns | Low |
| Benralizumab(60mg) | 1 | Some concerns | Low risk | No concerns | Major concerns | No concerns | No concerns | Low |
| Dupilumab | 2 | No concerns | Low risk | No concerns | No concerns | No concerns | No concerns | High |
| Fenebrutinib(150mg) | 1 | Some concerns | Low risk | No concerns | Major concerns | No concerns | No concerns | Low |
| Fenebrutinib (400mg) | 2 | Some concerns | Low risk | No concerns | No concerns | No concerns | No concerns | Moderate |
| Fenebrutinib(50mg) | 1 | Some concerns | Low risk | No concerns | Major concerns | No concerns | No concerns | Low |
| Ligelizumab(120mg) | 2 | No concerns | Low risk | No concerns | No concerns | Some concerns | Some concerns | Low |
| Ligelizumab(240mg) | 1 | No concerns | Low risk | No concerns | Major concerns | No concerns | No concerns | Low |
| Ligelizumab(24mg) | 1 | No concerns | Low risk | No concerns | No concerns | No concerns | Some concerns | Moderate |
| Ligelizumab(72mg) | 3 | No concerns | Low risk | No concerns | No concerns | Some concerns | No concerns | Moderate |
| Omalizumab(150mg) | 4 | No concerns | Low risk | No concerns | No concerns | No concerns | No concerns | High |
| Omalizumab (300mg) | 12 | No concerns | Low risk | No concerns | No concerns | No concerns | No concerns | High |
| Omalizumab (600mg) | 1 | No concerns | Low risk | No concerns | No concerns | No concerns | No concerns | High |
| Omalizumab (75mg) | 3 | No concerns | Low risk | No concerns | No concerns | No concerns | No concerns | High |
| Quilizumab | 1 | Some concerns | Low risk | No concerns | Major concerns | No concerns | No concerns | Low |
| Remibrutinib(100mg bid) | 1 | No concerns | Low risk | No concerns | No concerns | No concerns | No concerns | High |
| Remibrutinib(100mg qd) | 1 | No concerns | Low risk | No concerns | No concerns | Some concerns | No concerns | Moderate |
| Remibrutinib(10mg bid) | 1 | No concerns | Low risk | No concerns | No concerns | No concerns | No concerns | High |
| Remibrutinib(10mg qd) | 1 | No concerns | Low risk | No concerns | No concerns | No concerns | No concerns | High |
| Remibrutinib(25mg bid) | 3 | No concerns | Low risk | No concerns | No concerns | No concerns | No concerns | High |
| Remibrutinib(35mg qd) | 1 | No concerns | Low risk | No concerns | No concerns | No concerns | No concerns | High |
| Rilzabrutinib | 1 | Some concerns | Low risk | No concerns | No concerns | No concerns | No concerns | Moderate |
| Tezepelumab(210mg) | 1 | Some concerns | Low risk | No concerns | Some concerns | No concerns | No concerns | Low |
| Tezepelumab(420mg) | 1 | Some concerns | Low risk | No concerns | Some concerns | No concerns | No concerns | Low |

Minimum clinically important difference set to 0.2

**eFigure 3**. Network graphs for proportion of patients achieved well-controlled disease (UAS7≤6).


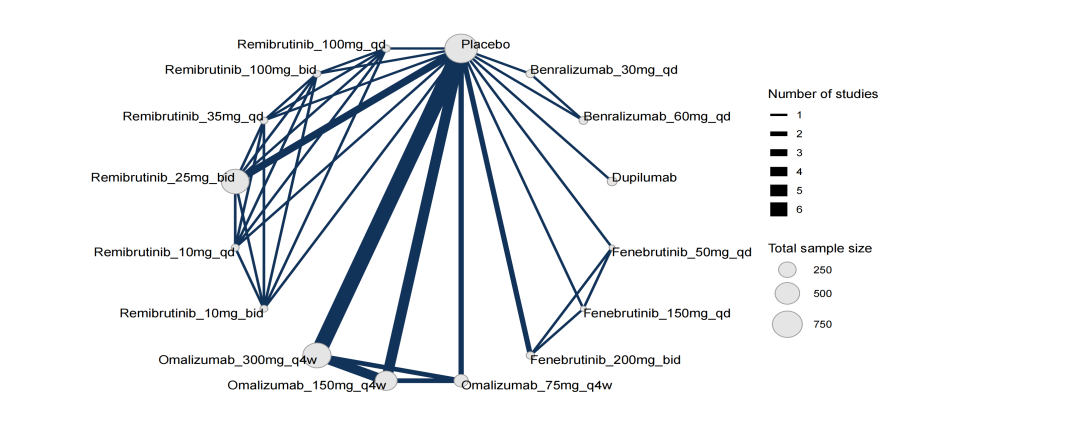


**eFigure 4**. Forest plot for proportion of patients achieved well-controlled disease (UAS7≤6)。


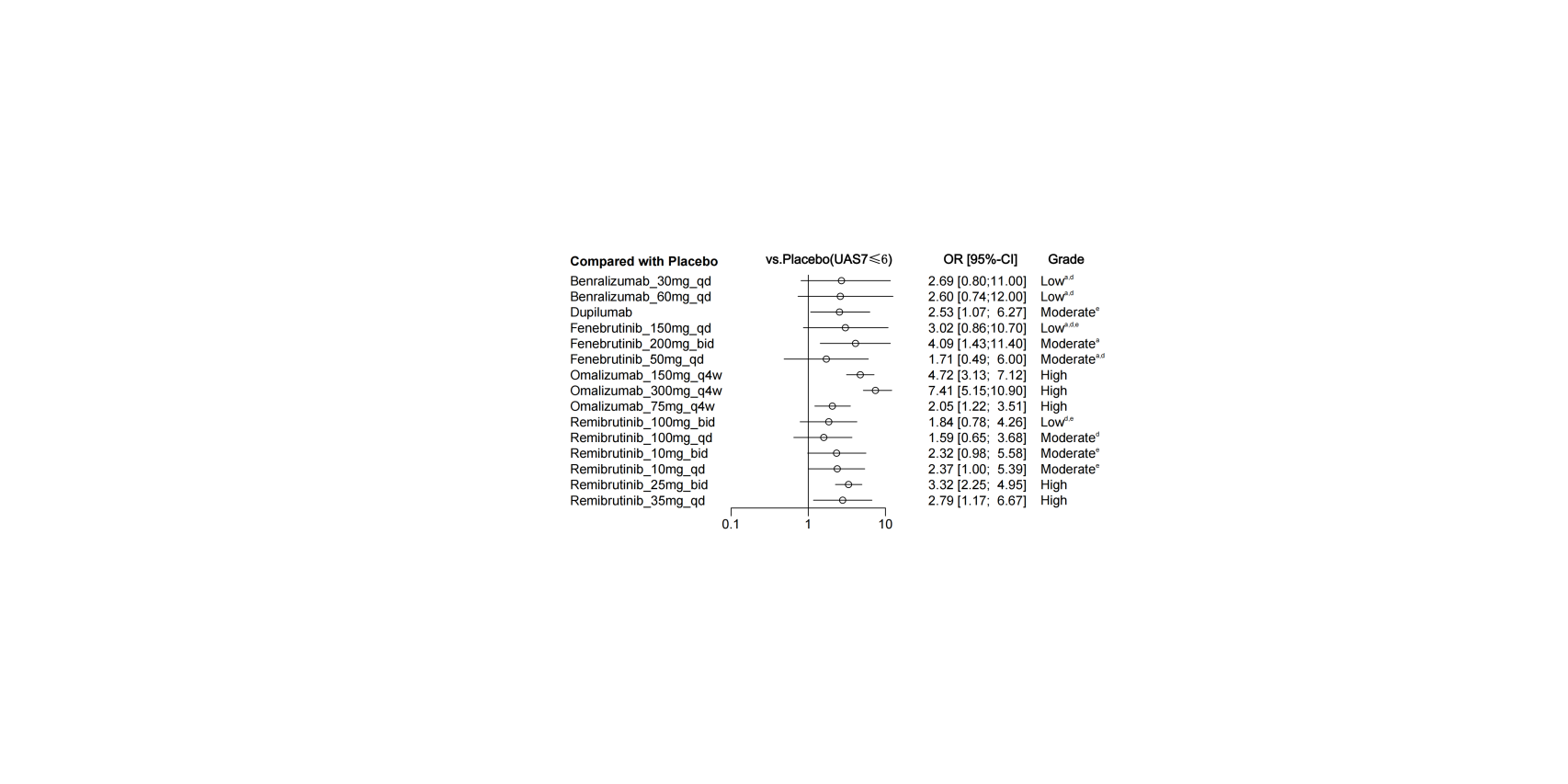


a:Within-study bias; b:Reporting bias; c:Indirectness; d:Imprecision; e:Heterogeneity; f:Incoherence

**eTable 4.** League table and the Surface Under the Cumulative Ranking (SUCRA): proportion of patients achieved well-controlled disease (UAS7≤6).

|  | Benralizumab 30mg qd | Benralizumab 60mg qd | Dupilumab | Fenebrutinib 150mg qd | Fenebrutinib 200mg bid | Fenebrutinib 50mg qd | Omalizumab 150mg q4w | Omalizumab 300mg q4w | Omalizumab 75mg q4w | Placebo | Remibrutinib 100mg bid | Remibrutinib 100mg qd | Remibrutinib 10mg bid | Remibrutinib 10mg qd | Remibrutinib 25mg bid | Remibrutinib 35mg qd |
| --- | --- | --- | --- | --- | --- | --- | --- | --- | --- | --- | --- | --- | --- | --- | --- | --- |
| Benralizumab 30mg qd | Benralizumab 30mg qd | 0.95 (0.36, 2.66) | 0.93 (0.19, 4.31) | 1.07 (0.17, 7.14) | 1.48 (0.27, 8.43) | 0.63 (0.09, 3.94) | 1.75 (0.41, 6.27) | 2.76 (0.66, 9.73) | 0.76 (0.17, 2.88) | 0.37 (0.09, 1.24) | 0.68 (0.13, 3.02) | 0.59 (0.11, 2.57) | 0.85 (0.17, 3.81) | 0.87 (0.16, 3.95) | 1.25 (0.28, 4.32) | 1.05 (0.2, 4.58) |
| Benralizumab 60mg qd | 1.05 (0.38, 2.8) | Benralizumab 60mg qd | 0.97 (0.19, 4.68) | 1.14 (0.16, 7.53) | 1.56 (0.26, 8.8) | 0.66 (0.09, 4.44) | 1.82 (0.38, 6.86) | 2.89 (0.62, 10.67) | 0.8 (0.17, 3.16) | 0.38 (0.08, 1.36) | 0.72 (0.12, 3.3) | 0.62 (0.11, 2.69) | 0.9 (0.18, 4.06) | 0.9 (0.17, 4.24) | 1.29 (0.28, 4.77) | 1.1 (0.19, 4.91) |
| Dupilumab | 1.07 (0.23, 5.33) | 1.03 (0.21, 5.38) | Dupilumab | 1.18 (0.26, 5.38) | 1.6 (0.43, 5.86) | 0.68 (0.15, 3.1) | 1.86 (0.68, 4.81) | 2.93 (1.07, 7.59) | 0.82 (0.27, 2.24) | 0.39 (0.16, 0.93) | 0.73 (0.22, 2.46) | 0.63 (0.18, 2.07) | 0.92 (0.26, 3.06) | 0.93 (0.26, 3.14) | 1.32 (0.47, 3.4) | 1.12 (0.31, 3.77) |
| Fenebrutinib 150mg qd | 0.93 (0.14, 5.77) | 0.88 (0.13, 6.12) | 0.85 (0.19, 3.79) | Fenebrutinib 150mg qd | 1.35 (0.43, 4.41) | 0.57 (0.16, 2.03) | 1.58 (0.41, 5.82) | 2.5 (0.66, 9) | 0.69 (0.18, 2.68) | 0.33 (0.09, 1.16) | 0.6 (0.14, 2.81) | 0.51 (0.12, 2.36) | 0.77 (0.16, 3.66) | 0.78 (0.17, 3.55) | 1.11 (0.29, 4.09) | 0.93 (0.2, 4.34) |
| Fenebrutinib 200mg bid | 0.68 (0.12, 3.64) | 0.64 (0.11, 3.9) | 0.62 (0.17, 2.32) | 0.74 (0.23, 2.35) | Fenebrutinib 200mg bid | 0.43 (0.12, 1.45) | 1.17 (0.38, 3.54) | 1.84 (0.58, 5.45) | 0.51 (0.16, 1.61) | 0.24 (0.09, 0.7) | 0.45 (0.12, 1.73) | 0.38 (0.1, 1.49) | 0.57 (0.15, 2.15) | 0.57 (0.15, 2.25) | 0.82 (0.26, 2.5) | 0.68 (0.18, 2.6) |
| Fenebrutinib 50mg qd | 1.58 (0.25, 11.3) | 1.52 (0.23, 11.41) | 1.47 (0.32, 6.84) | 1.75 (0.49, 6.31) | 2.34 (0.69, 8.11) | Fenebrutinib 50mg qd | 2.77 (0.74, 10.43) | 4.38 (1.16, 16.35) | 1.21 (0.31, 4.82) | 0.58 (0.17, 2.06) | 1.08 (0.23, 4.97) | 0.92 (0.2, 4.22) | 1.36 (0.28, 6.77) | 1.37 (0.29, 6.2) | 1.94 (0.52, 7.33) | 1.64 (0.35, 7.5) |
| Omalizumab 150mg q4w | 0.57 (0.16, 2.44) | 0.55 (0.15, 2.64) | 0.54 (0.21, 1.48) | 0.63 (0.17, 2.42) | 0.85 (0.28, 2.63) | 0.36 (0.1, 1.35) | Omalizumab 150mg q4w | 1.58 (1.13, 2.25) | 0.44 (0.26, 0.72) | 0.21 (0.14, 0.32) | 0.39 (0.15, 0.99) | 0.34 (0.13, 0.86) | 0.49 (0.19, 1.29) | 0.5 (0.19, 1.27) | 0.71 (0.4, 1.24) | 0.59 (0.22, 1.56) |
| Omalizumab 300mg q4w | 0.36 (0.1, 1.51) | 0.35 (0.09, 1.63) | 0.34 (0.13, 0.93) | 0.4 (0.11, 1.52) | 0.54 (0.18, 1.71) | 0.23 (0.06, 0.87) | 0.63 (0.44, 0.88) | Omalizumab 300mg q4w | 0.28 (0.17, 0.46) | 0.13 (0.09, 0.19) | 0.25 (0.1, 0.62) | 0.21 (0.08, 0.53) | 0.31 (0.12, 0.8) | 0.32 (0.12, 0.8) | 0.45 (0.26, 0.75) | 0.38 (0.14, 0.97) |
| Omalizumab 75mg q4w | 1.31 (0.35, 5.83) | 1.24 (0.32, 5.88) | 1.22 (0.45, 3.68) | 1.45 (0.37, 5.6) | 1.96 (0.62, 6.4) | 0.83 (0.21, 3.21) | 2.29 (1.38, 3.78) | 3.59 (2.2, 6.01) | Omalizumab 75mg q4w | 0.49 (0.28, 0.82) | 0.9 (0.33, 2.39) | 0.78 (0.27, 2.06) | 1.13 (0.4, 3.1) | 1.15 (0.4, 3.08) | 1.63 (0.83, 3.1) | 1.36 (0.48, 3.76) |
| Placebo | 2.69 (0.8, 10.99) | 2.6 (0.74, 12.02) | 2.53 (1.07, 6.27) | 3.02 (0.86, 10.69) | 4.09 (1.43, 11.39) | 1.71 (0.49, 6) | 4.72 (3.13, 7.12) | 7.41 (5.15, 10.91) | 2.05 (1.22, 3.51) | Placebo | 1.84 (0.78, 4.26) | 1.59 (0.65, 3.68) | 2.32 (0.98, 5.58) | 2.37 (1, 5.39) | 3.32 (2.25, 4.95) | 2.79 (1.17, 6.67) |
| Remibrutinib 100mg bid | 1.46 (0.33, 7.53) | 1.4 (0.3, 8.04) | 1.38 (0.41, 4.58) | 1.66 (0.36, 7.15) | 2.21 (0.58, 8.27) | 0.93 (0.2, 4.39) | 2.56 (1.01, 6.62) | 4.03 (1.61, 10.38) | 1.11 (0.42, 3.07) | 0.54 (0.23, 1.28) | Remibrutinib 100mg bid | 0.85 (0.34, 2.15) | 1.26 (0.49, 3.19) | 1.28 (0.5, 3.26) | 1.8 (0.79, 4.21) | 1.51 (0.6, 3.89) |
| Remibrutinib 100mg qd | 1.7 (0.39, 9.07) | 1.62 (0.37, 9.48) | 1.59 (0.48, 5.63) | 1.95 (0.42, 8.65) | 2.6 (0.67, 9.72) | 1.09 (0.24, 5.02) | 2.95 (1.16, 7.86) | 4.66 (1.89, 12.54) | 1.28 (0.49, 3.66) | 0.63 (0.27, 1.54) | 1.17 (0.47, 2.96) | Remibrutinib 100mg qd | 1.46 (0.58, 3.92) | 1.47 (0.58, 3.77) | 2.12 (0.91, 5.03) | 1.76 (0.7, 4.63) |
| Remibrutinib 10mg bid | 1.17 (0.26, 5.76) | 1.11 (0.25, 5.61) | 1.09 (0.33, 3.9) | 1.3 (0.27, 6.1) | 1.76 (0.47, 6.88) | 0.73 (0.15, 3.56) | 2.04 (0.78, 5.28) | 3.19 (1.25, 8.29) | 0.89 (0.32, 2.49) | 0.43 (0.18, 1.02) | 0.8 (0.31, 2.05) | 0.69 (0.26, 1.71) | Remibrutinib 10mg bid | 1.01 (0.38, 2.56) | 1.43 (0.6, 3.41) | 1.2 (0.48, 3.03) |
| Remibrutinib 10mg qd | 1.15 (0.25, 6.18) | 1.11 (0.24, 6.01) | 1.07 (0.32, 3.81) | 1.28 (0.28, 5.74) | 1.75 (0.44, 6.67) | 0.73 (0.16, 3.5) | 1.99 (0.79, 5.27) | 3.15 (1.26, 8.29) | 0.87 (0.32, 2.48) | 0.42 (0.19, 1) | 0.78 (0.31, 1.99) | 0.68 (0.26, 1.71) | 0.99 (0.39, 2.65) | Remibrutinib 10mg qd | 1.42 (0.62, 3.3) | 1.19 (0.46, 3.18) |
| Remibrutinib 25mg bid | 0.8 (0.23, 3.51) | 0.77 (0.21, 3.52) | 0.76 (0.29, 2.11) | 0.9 (0.24, 3.48) | 1.22 (0.4, 3.79) | 0.52 (0.14, 1.93) | 1.41 (0.81, 2.52) | 2.22 (1.33, 3.9) | 0.61 (0.32, 1.21) | 0.3 (0.2, 0.44) | 0.56 (0.24, 1.26) | 0.47 (0.2, 1.09) | 0.7 (0.29, 1.68) | 0.71 (0.3, 1.62) | Remibrutinib 25mg bid | 0.84 (0.35, 1.99) |
| Remibrutinib 35mg qd | 0.95 (0.22, 4.92) | 0.91 (0.2, 5.22) | 0.89 (0.27, 3.27) | 1.08 (0.23, 4.95) | 1.47 (0.38, 5.6) | 0.61 (0.13, 2.82) | 1.69 (0.64, 4.53) | 2.66 (1.03, 7.02) | 0.73 (0.27, 2.1) | 0.36 (0.15, 0.86) | 0.66 (0.26, 1.67) | 0.57 (0.22, 1.42) | 0.83 (0.33, 2.1) | 0.84 (0.31, 2.18) | 1.2 (0.5, 2.83) | Remibrutinib 35mg qd |
| SUCRA | 0.5388 | 0.5212 | 0.4808 | 0.547 | 0.7325 | 0.3036 | 0.822 | 0.9699 | 0.3697 | 0.0428 | 0.3195 | 0.2412 | 0.4451 | 0.4454 | 0.6604 | 0.56 |

For proportion of patients achieved well-controlled disease (UAS7≤6), Network meta-analysis results are expressed as OR (95% CI). OR more than 1 indicate that the treatment specified in the row is more effective than that specified in the column. The bottom row contains the Surface Under the Cumulative Ranking (SUCRA) value for the column-defining treatment. Both in efficacy and safety, a higher score on the lower surface under the cumulative ranking curve (SUCRA) indicate better efficacy and higher safety.

**eTable 5.** GRADE assessment by CINeMA: proportion of patients achieved well-controlled disease (UAS7≤6).

| **Treatment  comparison  (reference: placebo)** | **Number of studies** | **Within-study bias** | **Reporting bias** | **Indirectness** | **Imprecision** | **Heterogeneity** | **Incoherence** | **Confidence rating** |
| --- | --- | --- | --- | --- | --- | --- | --- | --- |
| **Benralizumab 30mg** | 1 | Some concerns | Low risk | No concerns | Major concerns | No concerns | No concerns | Low |
| **Benralizumab 60mg** | 1 | Some concerns | Low risk | No concerns | Major concerns | No concerns | No concerns | Low |
| **Dupilumab** | 1 | No concerns | Low risk | No concerns | No concerns | Some concerns | No concerns | Moderate |
| **Fenebrutinib 150mg qd** | 1 | Some concerns | Low risk | No concerns | Some concerns | Some concerns | No concerns | Low |
| **Fenebrutinib 200mg bid** | 2 | Some concerns | Low risk | No concerns | No concerns | No concerns | No concerns | Moderate |
| **Fenebrutinib 50mg qd** | 1 | Some concerns | Low risk | No concerns | Major concerns | No concerns | No concerns | Moderate |
| **Omalizumab 150mg** | 4 | No concerns | Low risk | No concerns | No concerns | No concerns | No concerns | High |
| **Omalizumab 300mg** | 6 | No concerns | Low risk | No concerns | No concerns | No concerns | No concerns | High |
| **Omalizumab 75mg** | 2 | No concerns | Low risk | No concerns | No concerns | No concerns | No concerns | High |
| **Remibrutinib 100mg bid** | 1 | No concerns | Low risk | No concerns | Some concerns | Some concerns | No concerns | Low |
| **Remibrutinib 100mg qd** | 1 | No concerns | Low risk | No concerns | Major concerns | No concerns | No concerns | Moderate |
| **Remibrutinib 10mg bid** | 1 | No concerns | Low risk | No concerns | No concerns | Some concerns | No concerns | Moderate |
| **Remibrutinib 10mg qd** | 1 | No concerns | Low risk | No concerns | No concerns | Some concerns | No concerns | Moderate |
| **Remibrutinib 25mg bid** | 3 | No concerns | Low risk | No concerns | No concerns | No concerns | No concerns | High |
| **Remibrutinib 35mg qd** | 1 | No concerns | Low risk | No concerns | No concerns | No concerns | No concerns | High |

Minimum clinically important difference set to 1.2

**eFigure 5.** Network graphs for proportion of patients achieved complete response (UAS7=0).


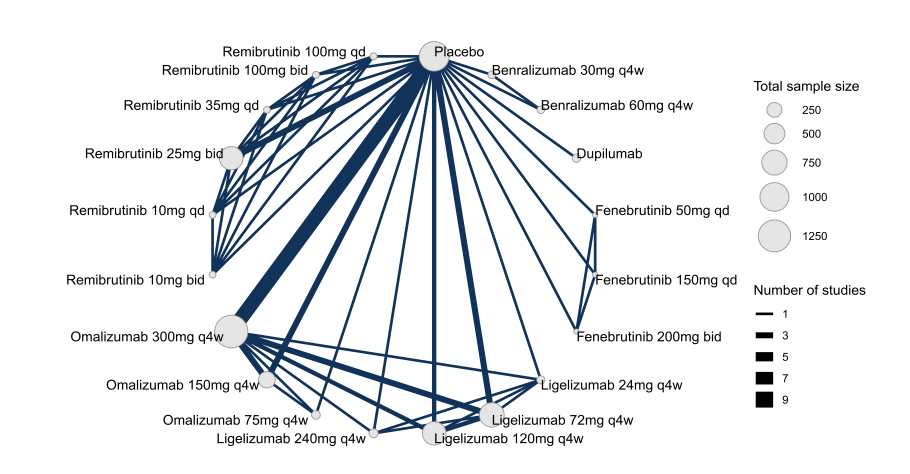


**eFigure 6**. Forest plot for proportion of patients achieved complete response (UAS7=0).


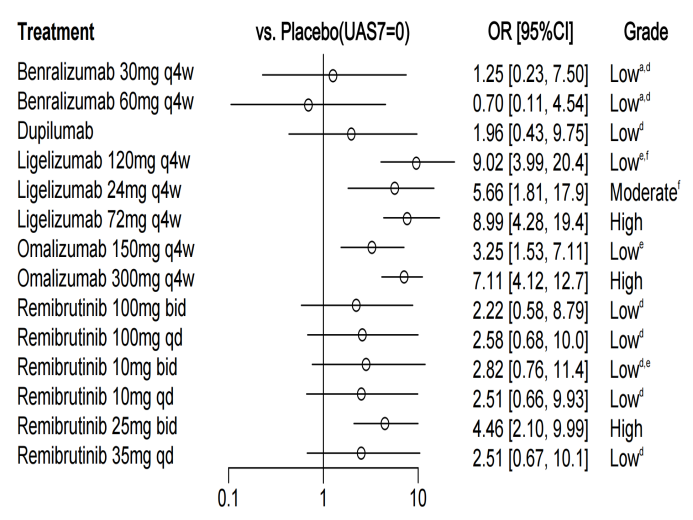


a:Within-study bias; b:Reporting bias; c:Indirectness; d:Imprecision; e:Heterogeneity; f:Incoherence

**eTable 6.** League table and the Surface Under the Cumulative Ranking (SUCRA): proportion of patients achieved complete response (UAS7=0).

|  | Ben30mg | Ben60mg | Dup | Fen150mg | Fen200mg | Fen50mg | Lig120mg | Lig240mg | Lig24mg | Lig72mg | Oma150mg | Oma300mg | Oma75mg | Placebo | Remi100mg bid | Remi100mg qd | Remi10mg bid | Remi10mg qd | Remi25mg bid | Remi35mg qd |
| --- | --- | --- | --- | --- | --- | --- | --- | --- | --- | --- | --- | --- | --- | --- | --- | --- | --- | --- | --- | --- |
| Ben30mg | Ben30mg | 0.55 (0.09, 3) | 1.63 (0.15, 17.07) | 9.57 (0.45, 475.22) | 18.81 (0.91, 958.57) | 3.96 (0.16, 206.29) | 7.32 (0.99, 49.89) | 8.54 (1.01, 72.06) | 4.51 (0.53, 35.62) | 7.29 (1.03, 48.54) | 2.65 (0.37, 17.37) | 5.78 (0.87, 36.14) | 38.23 (4.36, 308.36) | 0.81 (0.13, 4.47) | 1.83 (0.19, 16.13) | 2.12 (0.23, 19.07) | 2.37 (0.26, 21.07) | 2.16 (0.22, 18.6) | 3.69 (0.51, 24.12) | 2.14 (0.23, 18.43) |
| Ben60mg | 1.81 (0.33, 10.81) | Ben60mg | 2.97 (0.26, 34.7) | 17.16 (0.77, 909.1) | 34.37 (1.58, 1917.8) | 7.28 (0.28, 412.69) | 13.31 (1.71, 101.9) | 15.37 (1.74, 146.63) | 8.29 (0.93, 72.26) | 13.26 (1.76, 99.9) | 4.8 (0.64, 36.43) | 10.53 (1.48, 74.53) | 69.34 (7.5, 643.47) | 1.49 (0.23, 9.52) | 3.33 (0.34, 33.45) | 3.82 (0.4, 39.28) | 4.25 (0.45, 43.08) | 3.87 (0.39, 39.72) | 6.69 (0.89, 50.33) | 3.85 (0.39, 38.88) |
| Dup | 0.61 (0.06, 6.54) | 0.34 (0.03, 3.85) | Dup | 5.68 (0.31, 285.62) | 11.38 (0.64, 582.61) | 2.39 (0.11, 126.28) | 4.49 (0.75, 26.03) | 5.23 (0.75, 37.46) | 2.77 (0.4, 19.21) | 4.48 (0.77, 25.24) | 1.62 (0.28, 9.24) | 3.57 (0.66, 18.36) | 23.49 (3.28, 166.4) | 0.5 (0.1, 2.34) | 1.14 (0.14, 8.89) | 1.3 (0.17, 10.5) | 1.45 (0.19, 11.6) | 1.32 (0.17, 10.34) | 2.26 (0.39, 12.85) | 1.3 (0.17, 10.23) |
| Fen150mg | 0.1 (0, 2.24) | 0.06 (0, 1.3) | 0.18 (0, 3.26) | Fen150mg | 1.99 (0.38, 10.9) | 0.43 (0.06, 2.77) | 0.79 (0.02, 10.3) | 0.92 (0.02, 14.11) | 0.49 (0.01, 7.3) | 0.8 (0.02, 10.03) | 0.29 (0.01, 3.58) | 0.63 (0.02, 7.57) | 4.09 (0.09, 60.98) | 0.09 (0, 0.98) | 0.2 (0, 3.24) | 0.23 (0.01, 3.8) | 0.26 (0.01, 4.19) | 0.23 (0, 3.82) | 0.4 (0.01, 5.1) | 0.23 (0, 3.7) |
| Fen200mg | 0.05 (0, 1.09) | 0.03 (0, 0.63) | 0.09 (0, 1.56) | 0.5 (0.09, 2.62) | Fen200mg | 0.21 (0.03, 1.31) | 0.4 (0.01, 4.91) | 0.47 (0.01, 6.76) | 0.25 (0.01, 3.49) | 0.41 (0.01, 4.82) | 0.15 (0, 1.75) | 0.32 (0.01, 3.66) | 2.1 (0.05, 29.89) | 0.05 (0, 0.47) | 0.1 (0, 1.54) | 0.12 (0, 1.8) | 0.13 (0, 1.96) | 0.12 (0, 1.84) | 0.2 (0, 2.45) | 0.12 (0, 1.77) |
| Fen50mg | 0.25 (0, 6.21) | 0.14 (0, 3.59) | 0.42 (0.01, 8.92) | 2.35 (0.36, 17.98) | 4.73 (0.76, 35.91) | Fen50mg | 1.88 (0.04, 28.87) | 2.19 (0.05, 39.94) | 1.17 (0.03, 20.38) | 1.89 (0.04, 28.32) | 0.68 (0.02, 10.39) | 1.5 (0.04, 21.54) | 9.77 (0.21, 174.69) | 0.21 (0.01, 2.82) | 0.47 (0.01, 9.11) | 0.55 (0.01, 10.63) | 0.6 (0.01, 11.64) | 0.55 (0.01, 10.81) | 0.96 (0.02, 14.8) | 0.54 (0.01, 10.26) |
| Lig120mg | 0.14 (0.02, 1.01) | 0.08 (0.01, 0.59) | 0.22 (0.04, 1.33) | 1.26 (0.1, 52.53) | 2.48 (0.2, 106.84) | 0.53 (0.03, 22.87) | Lig120mg | 1.16 (0.35, 4.25) | 0.62 (0.2, 1.92) | 1 (0.47, 2.23) | 0.36 (0.14, 1) | 0.79 (0.38, 1.71) | 5.21 (1.39, 20.45) | 0.11 (0.05, 0.25) | 0.25 (0.05, 1.22) | 0.29 (0.06, 1.44) | 0.33 (0.07, 1.58) | 0.29 (0.06, 1.42) | 0.5 (0.17, 1.58) | 0.29 (0.06, 1.42) |
| Lig240mg | 0.12 (0.01, 0.99) | 0.07 (0.01, 0.58) | 0.19 (0.03, 1.32) | 1.09 (0.07, 49.02) | 2.11 (0.15, 97.02) | 0.46 (0.03, 21.15) | 0.86 (0.24, 2.86) | Lig240mg | 0.53 (0.14, 1.89) | 0.86 (0.27, 2.61) | 0.31 (0.08, 1.11) | 0.68 (0.21, 2.03) | 4.45 (0.9, 21.42) | 0.1 (0.03, 0.3) | 0.22 (0.03, 1.24) | 0.25 (0.04, 1.46) | 0.28 (0.05, 1.64) | 0.25 (0.04, 1.43) | 0.43 (0.1, 1.71) | 0.25 (0.04, 1.43) |
| Lig24mg | 0.22 (0.03, 1.88) | 0.12 (0.01, 1.08) | 0.36 (0.05, 2.48) | 2.05 (0.14, 89.04) | 4.02 (0.29, 178.58) | 0.86 (0.05, 38.95) | 1.61 (0.52, 4.94) | 1.88 (0.53, 7.24) | Lig24mg | 1.61 (0.54, 4.9) | 0.58 (0.17, 2.09) | 1.28 (0.44, 3.82) | 8.44 (1.81, 39.96) | 0.18 (0.06, 0.56) | 0.41 (0.07, 2.37) | 0.47 (0.08, 2.78) | 0.53 (0.09, 3.05) | 0.47 (0.08, 2.77) | 0.81 (0.21, 3.21) | 0.47 (0.08, 2.71) |
| Lig72mg | 0.14 (0.02, 0.97) | 0.08 (0.01, 0.57) | 0.22 (0.04, 1.29) | 1.25 (0.1, 52.9) | 2.45 (0.21, 104.29) | 0.53 (0.04, 22.71) | 1 (0.45, 2.14) | 1.17 (0.38, 3.75) | 0.62 (0.2, 1.85) | Lig72mg | 0.36 (0.14, 0.94) | 0.79 (0.41, 1.56) | 5.22 (1.42, 19.52) | 0.11 (0.05, 0.23) | 0.26 (0.05, 1.17) | 0.29 (0.06, 1.37) | 0.33 (0.07, 1.52) | 0.3 (0.06, 1.36) | 0.51 (0.17, 1.48) | 0.3 (0.06, 1.34) |
| Oma150mg | 0.38 (0.06, 2.72) | 0.21 (0.03, 1.57) | 0.62 (0.11, 3.55) | 3.49 (0.28, 146.31) | 6.85 (0.57, 287.26) | 1.47 (0.1, 62.19) | 2.77 (1, 7.29) | 3.23 (0.9, 12.3) | 1.72 (0.48, 6.02) | 2.75 (1.07, 7.06) | Oma150mg | 2.19 (1.07, 4.5) | 14.48 (4.31, 48.65) | 0.31 (0.14, 0.66) | 0.71 (0.14, 3.27) | 0.81 (0.17, 3.84) | 0.91 (0.19, 4.21) | 0.82 (0.17, 3.83) | 1.4 (0.46, 4.13) | 0.81 (0.17, 3.75) |
| Oma300mg | 0.17 (0.03, 1.15) | 0.09 (0.01, 0.68) | 0.28 (0.05, 1.51) | 1.58 (0.13, 63.85) | 3.1 (0.27, 126.63) | 0.67 (0.05, 27.55) | 1.27 (0.58, 2.61) | 1.47 (0.49, 4.67) | 0.78 (0.26, 2.29) | 1.26 (0.64, 2.46) | 0.46 (0.22, 0.93) | Oma300mg | 6.57 (2.07, 21.06) | 0.14 (0.08, 0.24) | 0.32 (0.07, 1.36) | 0.37 (0.09, 1.6) | 0.41 (0.09, 1.77) | 0.37 (0.09, 1.58) | 0.64 (0.24, 1.65) | 0.37 (0.08, 1.58) |
| Oma75mg | 0.03 (0, 0.23) | 0.01 (0, 0.13) | 0.04 (0.01, 0.3) | 0.24 (0.02, 10.98) | 0.48 (0.03, 21.52) | 0.1 (0.01, 4.68) | 0.19 (0.05, 0.72) | 0.22 (0.05, 1.11) | 0.12 (0.03, 0.55) | 0.19 (0.05, 0.71) | 0.07 (0.02, 0.23) | 0.15 (0.05, 0.48) | Oma75mg | 0.02 (0.01, 0.07) | 0.05 (0.01, 0.29) | 0.06 (0.01, 0.34) | 0.06 (0.01, 0.37) | 0.06 (0.01, 0.34) | 0.1 (0.02, 0.4) | 0.06 (0.01, 0.33) |
| Placebo | 1.25 (0.23, 7.50) | 0.70 (0.11, 4.54) | 1.96 (0.43, 9.75) | 11.13 (1.02, 439.69) | 22.01 (2.11, 874.11) | 4.7 (0.35, 187.19) | 9.02 (3.99, 20.40) | 10.45 (3.35, 35.72) | 5.66 (1.81, 17.90) | 8.99 (4.28, 19.40) | 3.25 (1.53, 7.11) | 7.11 (4.12, 12.70) | 46.75 (14.48, 156.25) | Placebo | 2.22 (0.58, 8.79) | 2.58 (0.68, 10.00) | 2.82 (0.76, 11.40) | 2.51 (0.66, 9.93) | 4.46 (2.10, 9.99) | 2.51 (0.67, 10.10) |
| Remi100mg bid | 0.55 (0.06, 5.18) | 0.3 (0.03, 2.95) | 0.88 (0.11, 7.24) | 5.06 (0.31, 234.74) | 9.87 (0.65, 464.05) | 2.12 (0.11, 103.08) | 3.93 (0.82, 19.26) | 4.57 (0.81, 28.79) | 2.43 (0.42, 14.51) | 3.91 (0.86, 18.91) | 1.42 (0.31, 6.93) | 3.1 (0.74, 13.84) | 20.72 (3.47, 126.51) | 0.44 (0.11, 1.76) | Remi100mg bid | 1.16 (0.28, 4.91) | 1.3 (0.3, 5.47) | 1.17 (0.27, 5.02) | 1.99 (0.54, 7.55) | 1.16 (0.27, 5.14) |
| Remi100mg qd | 0.47 (0.05, 4.26) | 0.26 (0.03, 2.51) | 0.77 (0.1, 5.9) | 4.37 (0.26, 193.25) | 8.62 (0.56, 400.92) | 1.83 (0.09, 86.08) | 3.43 (0.69, 16.13) | 4 (0.68, 24.03) | 2.12 (0.36, 11.99) | 3.41 (0.73, 15.92) | 1.24 (0.26, 5.74) | 2.71 (0.63, 11.59) | 17.91 (2.97, 106.58) | 0.38 (0.1, 1.42) | 0.86 (0.2, 3.61) | Remi100mg qd | 1.11 (0.26, 4.63) | 1.01 (0.24, 4.18) | 1.72 (0.46, 6.37) | 1 (0.23, 4.1) |
| Remi10mg bid | 0.42 (0.05, 3.92) | 0.24 (0.02, 2.23) | 0.69 (0.09, 5.31) | 3.88 (0.24, 180.63) | 7.66 (0.51, 364.71) | 1.66 (0.09, 79.61) | 3.07 (0.63, 14.83) | 3.58 (0.61, 21.91) | 1.9 (0.33, 10.85) | 3.06 (0.66, 14.35) | 1.1 (0.24, 5.35) | 2.44 (0.56, 10.73) | 16.1 (2.68, 99.58) | 0.34 (0.09, 1.3) | 0.77 (0.18, 3.3) | 0.9 (0.22, 3.87) | Remi10mg bid | 0.91 (0.21, 3.87) | 1.55 (0.41, 5.79) | 0.9 (0.21, 3.82) |
| Remi10mg qd | 0.46 (0.05, 4.46) | 0.26 (0.03, 2.57) | 0.76 (0.1, 6) | 4.38 (0.26, 200.26) | 8.61 (0.54, 405.28) | 1.83 (0.09, 88.26) | 3.4 (0.7, 16.38) | 3.94 (0.7, 24.32) | 2.11 (0.36, 12.23) | 3.39 (0.74, 16.21) | 1.23 (0.26, 5.84) | 2.7 (0.63, 11.71) | 17.87 (2.98, 108.35) | 0.38 (0.1, 1.43) | 0.86 (0.2, 3.7) | 0.99 (0.24, 4.23) | 1.1 (0.26, 4.71) | Remi10mg qd | 1.72 (0.46, 6.41) | 1 (0.23, 4.22) |
| Remi25mg bid | 0.27 (0.04, 1.94) | 0.15 (0.02, 1.13) | 0.44 (0.08, 2.57) | 2.51 (0.2, 103.75) | 4.88 (0.41, 202.65) | 1.04 (0.07, 45.09) | 1.98 (0.63, 6.03) | 2.3 (0.59, 9.85) | 1.23 (0.31, 4.81) | 1.98 (0.67, 5.87) | 0.71 (0.24, 2.15) | 1.57 (0.61, 4.16) | 10.34 (2.52, 43.03) | 0.22 (0.1, 0.48) | 0.5 (0.13, 1.87) | 0.58 (0.16, 2.17) | 0.65 (0.17, 2.41) | 0.58 (0.16, 2.19) | Remi25mg bid | 0.58 (0.15, 2.14) |
| Remi35mg qd | 0.47 (0.05, 4.44) | 0.26 (0.03, 2.58) | 0.77 (0.1, 6.05) | 4.36 (0.27, 203.26) | 8.66 (0.56, 395.28) | 1.84 (0.1, 90.37) | 3.4 (0.71, 16.51) | 3.97 (0.7, 24.93) | 2.13 (0.37, 12.43) | 3.39 (0.74, 16.38) | 1.23 (0.27, 5.95) | 2.69 (0.63, 11.97) | 17.79 (2.99, 109.48) | 0.38 (0.1, 1.47) | 0.86 (0.19, 3.76) | 1 (0.24, 4.36) | 1.11 (0.26, 4.82) | 1 (0.24, 4.33) | 1.72 (0.47, 6.61) | Remi35mg qd |
| **SUCRA** | 0.1957 | 0.0885 | 0.2954 | 0.7106 | 0.8559 | 0.5047 | 0.7589 | 0.7876 | 0.5902 | 0.7608 | 0.4118 | 0.6792 | 0.9654 | 0.0928 | 0.3056 | 0.3615 | 0.3888 | 0.352 | 0.5375 | 0.3569 |

For proportion of patients achieved complete response (UAS7=0), Network meta-analysis results are expressed as OR (95% CI). OR more than 1 indicate that the treatment specified in the row is more effective than that specified in the column. The bottom row contains the Surface Under the Cumulative Ranking (SUCRA) value for the column-defining treatment. Both in efficacy and safety, a higher score on the lower surface under the cumulative ranking curve (SUCRA) indicate better efficacy and higher safety.

**eTable 7.** GRADE assessment by CINeMA: proportion of patients achieved complete response (UAS7=0).

| Comparison | Number of studies | Within-study bias | Reporting bias | Indirectness | Imprecision | Heterogeneity | Incoherence | Confidence rating |
| --- | --- | --- | --- | --- | --- | --- | --- | --- |
| Benralizumab_30mg_qd | 1 | Some concerns | Low risk | No concerns | Major concerns | No concerns | No concerns | Low |
| Benralizumab_60mg_qd | 1 | Some concerns | Low risk | No concerns | Major concerns | No concerns | No concerns | Low |
| Dupilumab | 1 | No concerns | Low risk | No concerns | Major concerns | No concerns | No concerns | Low |
| Fenebrutinib_150mg_qd | 1 | Some concerns | Low risk | No concerns | Major concerns | No concerns | No concerns | Low |
| Fenebrutinib_200mg_bid | 1 | Some concerns | Low risk | No concerns | No concerns | Some concerns | No concerns | Low |
| Fenebrutinib_50mg_qd | 1 | Some concerns | Low risk | No concerns | Major concerns | No concerns | No concerns | Low |
| Ligelizumab_120mg_q4w | 2 | No concerns | Low risk | No concerns | No concerns | Some concerns | Some concerns | Low |
| Ligelizumab_240mg_q4w | 1 | No concerns | Low risk | No concerns | Major concerns | No concerns | No concerns | Low |
| Ligelizumab_24mg_q4w | 1 | No concerns | Low risk | No concerns | No concerns | No concerns | Some concerns | Moderate |
| Ligelizumab_72mg_q4w | 3 | No concerns | Low risk | No concerns | No concerns | No concerns | No concerns | High |
| Omalizumab_150mg_q4w | 3 | No concerns | Low risk | No concerns | No concerns | Major concerns | No concerns | Low |
| Omalizumab_300mg_q4w | 8 | No concerns | Low risk | No concerns | No concerns | No concerns | No concerns | High |
| Omalizumab_75mg_q4w | 1 | No concerns | Low risk | No concerns | No concerns | No concerns | No concerns | High |
| Remibrutinib_100mg_bid | 1 | No concerns | Low risk | No concerns | Major concerns | No concerns | No concerns | Low |
| Remibrutinib_100mg_qd | 1 | No concerns | Low risk | No concerns | Major concerns | No concerns | No concerns | Low |
| Remibrutinib_10mg_bid | 1 | No concerns | Low risk | No concerns | Some concerns | Some concerns | No concerns | Low |
| Remibrutinib_10mg_qd | 1 | No concerns | Low risk | No concerns | Major concerns | No concerns | No concerns | Low |
| Remibrutinib_25mg_bid | 3 | No concerns | Low risk | No concerns | No concerns | No concerns | No concerns | High |
| Remibrutinib_35mg_qd | 1 | No concerns | Low risk | No concerns | Major concerns | No concerns | No concerns | Low |

Minimum clinically important difference set to 1.2

**eTable 8.** League table and the Surface Under the Cumulative Ranking (SUCRA): adverse events (AEs).

|  | AZD | Ben30mg | Ben60mg | Dup | Fen150mg | Fen200mg | Fen50mg | Lig120mg | Lig240mg | Lig24mg | Lig72mg | Oma150mg | Oma300mg | Oma600mg | Oma75mg | Placebo | Qui | Remi100mg bid | Remi100mg qd | Remi10mg bid | Remi10mg qd | Remi25mg | Remi35mg |
| --- | --- | --- | --- | --- | --- | --- | --- | --- | --- | --- | --- | --- | --- | --- | --- | --- | --- | --- | --- | --- | --- | --- | --- |
| AZD | AZD | 1.12 (0.14, 8.9) | 0.94 (0.12, 7.6) | 1.14 (0.17, 8.15) | 2.17 (0.25, 19.74) | 1.65 (0.21, 13.4) | 1.64 (0.19, 14.58) | 1.9 (0.3, 12.41) | 1.42 (0.21, 10.26) | 2.16 (0.3, 16.66) | 1.74 (0.28, 11.28) | 1.59 (0.26, 10.13) | 1.65 (0.27, 10.38) | 1.55 (0.19, 13.26) | 1.17 (0.19, 7.71) | 1.11 (0.19, 6.86) | 1.89 (0.18, 20.73) | 2.01 (0.27, 16.09) | 1.47 (0.2, 11.67) | 0.99 (0.13, 7.7) | 2.13 (0.3, 17.64) | 1.23 (0.2, 8.14) | 1.19 (0.16, 9.31) |
| Ben30mg | 0.89 (0.11, 7.02) | Ben30mg | 0.84 (0.33, 2.15) | 1.03 (0.29, 3.64) | 1.94 (0.38, 9.69) | 1.5 (0.36, 6.14) | 1.48 (0.29, 7.49) | 1.72 (0.57, 5.14) | 1.27 (0.35, 4.46) | 1.93 (0.51, 7.49) | 1.56 (0.53, 4.63) | 1.43 (0.49, 4.15) | 1.48 (0.52, 4.2) | 1.39 (0.31, 6.26) | 1.06 (0.35, 3.22) | 1 (0.37, 2.71) | 1.68 (0.26, 10.73) | 1.81 (0.47, 7.26) | 1.33 (0.35, 5.2) | 0.89 (0.23, 3.48) | 1.93 (0.5, 7.61) | 1.11 (0.37, 3.35) | 1.08 (0.28, 4.24) |
| Ben60mg | 1.07 (0.13, 8.32) | 1.19 (0.46, 3.02) | Ben60mg | 1.23 (0.34, 4.33) | 2.3 (0.45, 11.9) | 1.78 (0.41, 7.43) | 1.77 (0.35, 8.8) | 2.04 (0.68, 6.24) | 1.51 (0.41, 5.55) | 2.29 (0.59, 9.07) | 1.86 (0.62, 5.67) | 1.7 (0.57, 5.14) | 1.76 (0.61, 5.15) | 1.65 (0.36, 7.66) | 1.26 (0.4, 4.02) | 1.19 (0.43, 3.37) | 2.02 (0.31, 12.98) | 2.16 (0.54, 8.62) | 1.58 (0.41, 6.21) | 1.06 (0.27, 4.16) | 2.31 (0.58, 9.09) | 1.31 (0.44, 4.13) | 1.28 (0.33, 4.95) |
| Dup | 0.88 (0.12, 5.91) | 0.97 (0.27, 3.43) | 0.82 (0.23, 2.9) | Dup | 1.89 (0.43, 8.37) | 1.45 (0.41, 5.18) | 1.44 (0.33, 6.27) | 1.66 (0.7, 4.07) | 1.23 (0.41, 3.61) | 1.88 (0.58, 6.04) | 1.53 (0.63, 3.62) | 1.39 (0.59, 3.25) | 1.45 (0.63, 3.24) | 1.34 (0.35, 5.25) | 1.04 (0.41, 2.56) | 0.97 (0.45, 2.1) | 1.64 (0.29, 9.42) | 1.76 (0.53, 5.98) | 1.28 (0.4, 4.31) | 0.87 (0.27, 2.87) | 1.88 (0.57, 6.35) | 1.07 (0.45, 2.64) | 1.04 (0.32, 3.5) |
| Fen150mg | 0.46 (0.05, 4.07) | 0.52 (0.1, 2.62) | 0.43 (0.08, 2.24) | 0.53 (0.12, 2.33) | Fen150mg | 0.76 (0.22, 2.72) | 0.76 (0.2, 2.91) | 0.88 (0.23, 3.41) | 0.65 (0.15, 2.91) | 1 (0.22, 4.66) | 0.81 (0.21, 3.03) | 0.73 (0.2, 2.78) | 0.76 (0.21, 2.81) | 0.71 (0.13, 3.91) | 0.54 (0.14, 2.12) | 0.51 (0.14, 1.84) | 0.87 (0.12, 6.39) | 0.94 (0.19, 4.57) | 0.68 (0.14, 3.27) | 0.46 (0.09, 2.21) | 1 (0.2, 4.9) | 0.57 (0.15, 2.19) | 0.56 (0.11, 2.64) |
| Fen200mg | 0.61 (0.07, 4.71) | 0.67 (0.16, 2.78) | 0.56 (0.13, 2.41) | 0.69 (0.19, 2.44) | 1.31 (0.37, 4.61) | Fen200mg | 0.99 (0.28, 3.55) | 1.15 (0.38, 3.51) | 0.85 (0.24, 3.05) | 1.3 (0.34, 5.07) | 1.06 (0.35, 3.13) | 0.96 (0.33, 2.84) | 1 (0.35, 2.83) | 0.93 (0.21, 4.25) | 0.71 (0.23, 2.18) | 0.67 (0.24, 1.84) | 1.14 (0.18, 7.02) | 1.23 (0.31, 4.82) | 0.9 (0.23, 3.44) | 0.6 (0.15, 2.35) | 1.31 (0.34, 5.12) | 0.75 (0.25, 2.24) | 0.72 (0.19, 2.8) |
| Fen50mg | 0.61 (0.07, 5.4) | 0.68 (0.13, 3.4) | 0.57 (0.11, 2.89) | 0.7 (0.16, 3.03) | 1.31 (0.34, 5.1) | 1.01 (0.28, 3.53) | Fen50mg | 1.17 (0.31, 4.36) | 0.86 (0.2, 3.69) | 1.31 (0.29, 5.96) | 1.06 (0.28, 3.93) | 0.97 (0.26, 3.57) | 1.01 (0.28, 3.59) | 0.94 (0.18, 5.08) | 0.72 (0.19, 2.72) | 0.68 (0.19, 2.37) | 1.14 (0.16, 8.42) | 1.23 (0.26, 5.9) | 0.9 (0.19, 4.2) | 0.61 (0.13, 2.81) | 1.31 (0.28, 6.29) | 0.75 (0.2, 2.82) | 0.73 (0.16, 3.45) |
| Lig120mg | 0.53 (0.08, 3.28) | 0.58 (0.19, 1.75) | 0.49 (0.16, 1.48) | 0.6 (0.25, 1.44) | 1.13 (0.29, 4.35) | 0.87 (0.28, 2.61) | 0.86 (0.23, 3.25) | Lig120mg | 0.74 (0.32, 1.67) | 1.12 (0.46, 2.77) | 0.92 (0.57, 1.4) | 0.84 (0.49, 1.38) | 0.87 (0.55, 1.29) | 0.81 (0.24, 2.61) | 0.62 (0.32, 1.13) | 0.58 (0.37, 0.89) | 0.98 (0.19, 4.96) | 1.06 (0.38, 2.97) | 0.77 (0.28, 2.1) | 0.52 (0.19, 1.41) | 1.13 (0.4, 3.17) | 0.65 (0.35, 1.22) | 0.63 (0.23, 1.72) |
| Lig240mg | 0.71 (0.1, 4.82) | 0.79 (0.22, 2.82) | 0.66 (0.18, 2.42) | 0.81 (0.28, 2.43) | 1.53 (0.34, 6.73) | 1.17 (0.33, 4.23) | 1.16 (0.27, 5.11) | 1.35 (0.6, 3.13) | Lig240mg | 1.52 (0.56, 4.27) | 1.23 (0.57, 2.68) | 1.13 (0.49, 2.58) | 1.17 (0.54, 2.49) | 1.1 (0.28, 4.2) | 0.84 (0.34, 2.03) | 0.79 (0.37, 1.73) | 1.33 (0.24, 7.76) | 1.43 (0.42, 4.9) | 1.04 (0.33, 3.47) | 0.7 (0.22, 2.33) | 1.52 (0.45, 5.27) | 0.87 (0.36, 2.18) | 0.84 (0.26, 2.85) |
| Lig24mg | 0.46 (0.06, 3.34) | 0.52 (0.13, 1.97) | 0.44 (0.11, 1.69) | 0.53 (0.17, 1.73) | 1 (0.21, 4.65) | 0.77 (0.2, 2.91) | 0.76 (0.17, 3.48) | 0.89 (0.36, 2.2) | 0.66 (0.23, 1.8) | Lig24mg | 0.81 (0.33, 1.94) | 0.74 (0.29, 1.85) | 0.77 (0.31, 1.82) | 0.72 (0.17, 2.95) | 0.55 (0.2, 1.46) | 0.52 (0.21, 1.25) | 0.87 (0.14, 5.26) | 0.94 (0.25, 3.46) | 0.68 (0.2, 2.47) | 0.46 (0.13, 1.65) | 1 (0.27, 3.65) | 0.57 (0.22, 1.56) | 0.55 (0.16, 2) |
| Lig72mg | 0.58 (0.09, 3.59) | 0.64 (0.22, 1.87) | 0.54 (0.18, 1.61) | 0.66 (0.28, 1.58) | 1.24 (0.33, 4.74) | 0.95 (0.32, 2.82) | 0.94 (0.25, 3.55) | 1.09 (0.72, 1.74) | 0.81 (0.37, 1.76) | 1.23 (0.51, 3.04) | Lig72mg | 0.91 (0.56, 1.5) | 0.95 (0.64, 1.38) | 0.89 (0.27, 2.85) | 0.68 (0.37, 1.22) | 0.64 (0.43, 0.96) | 1.08 (0.22, 5.42) | 1.16 (0.41, 3.23) | 0.85 (0.32, 2.29) | 0.57 (0.21, 1.54) | 1.24 (0.45, 3.44) | 0.7 (0.39, 1.32) | 0.69 (0.25, 1.88) |
| Oma150mg | 0.63 (0.1, 3.87) | 0.7 (0.24, 2.05) | 0.59 (0.19, 1.74) | 0.72 (0.31, 1.69) | 1.36 (0.36, 5.1) | 1.04 (0.35, 3.06) | 1.03 (0.28, 3.85) | 1.2 (0.72, 2.06) | 0.89 (0.39, 2.03) | 1.35 (0.54, 3.47) | 1.09 (0.67, 1.8) | Oma150mg | 1.04 (0.73, 1.46) | 0.97 (0.3, 3.08) | 0.74 (0.44, 1.25) | 0.7 (0.49, 1.02) | 1.18 (0.24, 5.9) | 1.26 (0.46, 3.52) | 0.93 (0.35, 2.49) | 0.62 (0.24, 1.67) | 1.35 (0.5, 3.74) | 0.77 (0.44, 1.42) | 0.75 (0.28, 2.02) |
| Oma300mg | 0.61 (0.1, 3.7) | 0.67 (0.24, 1.92) | 0.57 (0.19, 1.65) | 0.69 (0.31, 1.58) | 1.31 (0.36, 4.85) | 1 (0.35, 2.87) | 0.99 (0.28, 3.62) | 1.15 (0.77, 1.81) | 0.85 (0.4, 1.85) | 1.3 (0.55, 3.18) | 1.05 (0.72, 1.57) | 0.96 (0.68, 1.37) | Oma300mg | 0.93 (0.3, 2.87) | 0.72 (0.44, 1.17) | 0.67 (0.52, 0.89) | 1.14 (0.23, 5.61) | 1.22 (0.46, 3.29) | 0.89 (0.35, 2.33) | 0.6 (0.24, 1.57) | 1.3 (0.49, 3.52) | 0.74 (0.45, 1.31) | 0.72 (0.28, 1.91) |
| Oma600mg | 0.65 (0.08, 5.3) | 0.72 (0.16, 3.26) | 0.61 (0.13, 2.8) | 0.75 (0.19, 2.89) | 1.4 (0.26, 7.63) | 1.07 (0.24, 4.83) | 1.06 (0.2, 5.67) | 1.24 (0.38, 4.09) | 0.91 (0.24, 3.51) | 1.39 (0.34, 5.77) | 1.13 (0.35, 3.69) | 1.03 (0.32, 3.31) | 1.07 (0.35, 3.32) | Oma600mg | 0.76 (0.25, 2.41) | 0.72 (0.24, 2.25) | 1.22 (0.18, 8.13) | 1.31 (0.3, 5.63) | 0.96 (0.23, 4.12) | 0.64 (0.15, 2.77) | 1.39 (0.33, 6.1) | 0.8 (0.24, 2.7) | 0.77 (0.18, 3.32) |
| Oma75mg | 0.85 (0.13, 5.35) | 0.95 (0.31, 2.9) | 0.79 (0.25, 2.49) | 0.97 (0.39, 2.45) | 1.84 (0.47, 7.19) | 1.41 (0.46, 4.33) | 1.39 (0.37, 5.38) | 1.61 (0.89, 3.08) | 1.19 (0.49, 2.93) | 1.83 (0.69, 4.9) | 1.47 (0.82, 2.7) | 1.35 (0.8, 2.29) | 1.4 (0.86, 2.28) | 1.31 (0.41, 4.07) | Oma75mg | 0.94 (0.58, 1.56) | 1.59 (0.32, 8.15) | 1.71 (0.59, 5.03) | 1.25 (0.45, 3.55) | 0.84 (0.3, 2.39) | 1.82 (0.64, 5.32) | 1.04 (0.55, 2.1) | 1.01 (0.36, 2.9) |
| Placebo | 0.89 (0.14, 5.29) | 1.01 (0.38, 2.66) | 0.86 (0.31, 2.26) | 1.03 (0.48, 2.18) | 2.00 (0.54, 7.08) | 1.50 (0.57, 3.94) | 1.50 (0.44, 5.18) | 1.73 (1.13, 2.67) | 1.28 (0.59, 2.67) | 1.95 (0.81, 4.72) | 1.58 (1.04, 2.33) | 1.44 (0.99, 2.05) | 1.50 (1.12, 1.93) | 1.39 (0.46, 4.21) | 1.07 (0.64, 1.71) | Placebo | 1.71 (0.36, 7.96) | 1.84 (0.75, 4.62) | 1.32 (0.55, 3.31) | 0.91 (0.37, 2.23) | 1.92 (0.78, 4.97) | 1.1 (0.72, 1.75) | 1.07 (0.44, 2.70) |
| Qui | 0.53 (0.05, 5.58) | 0.59 (0.09, 3.81) | 0.49 (0.08, 3.26) | 0.61 (0.11, 3.45) | 1.15 (0.16, 8.49) | 0.88 (0.14, 5.56) | 0.88 (0.12, 6.3) | 1.02 (0.2, 5.16) | 0.75 (0.13, 4.23) | 1.14 (0.19, 6.94) | 0.93 (0.18, 4.64) | 0.85 (0.17, 4.15) | 0.88 (0.18, 4.27) | 0.82 (0.12, 5.5) | 0.63 (0.12, 3.11) | 0.59 (0.12, 2.8) | Qui | 1.08 (0.18, 6.54) | 0.79 (0.13, 4.8) | 0.53 (0.09, 3.18) | 1.15 (0.19, 7.04) | 0.66 (0.13, 3.29) | 0.64 (0.1, 3.98) |
| Remi100mg bid | 0.5 (0.06, 3.73) | 0.55 (0.14, 2.15) | 0.46 (0.12, 1.85) | 0.57 (0.17, 1.88) | 1.06 (0.22, 5.24) | 0.82 (0.21, 3.23) | 0.82 (0.17, 3.81) | 0.95 (0.34, 2.66) | 0.7 (0.2, 2.38) | 1.06 (0.29, 3.96) | 0.86 (0.31, 2.42) | 0.79 (0.28, 2.15) | 0.82 (0.3, 2.19) | 0.76 (0.18, 3.38) | 0.59 (0.2, 1.7) | 0.55 (0.22, 1.41) | 0.93 (0.15, 5.69) | Remi100mg bid | 0.73 (0.26, 2.02) | 0.49 (0.17, 1.4) | 1.06 (0.38, 3.08) | 0.61 (0.24, 1.59) | 0.6 (0.21, 1.67) |
| Remi100mg qd | 0.68 (0.09, 4.92) | 0.75 (0.19, 2.86) | 0.63 (0.16, 2.44) | 0.78 (0.23, 2.51) | 1.46 (0.31, 6.96) | 1.12 (0.29, 4.32) | 1.11 (0.24, 5.15) | 1.29 (0.48, 3.53) | 0.96 (0.29, 3.04) | 1.47 (0.4, 5.04) | 1.18 (0.44, 3.15) | 1.08 (0.4, 2.85) | 1.12 (0.43, 2.85) | 1.04 (0.24, 4.32) | 0.8 (0.28, 2.22) | 0.75 (0.3, 1.86) | 1.27 (0.21, 7.71) | 1.36 (0.49, 3.8) | Remi100mg qd | 0.67 (0.24, 1.83) | 1.45 (0.53, 4.11) | 0.83 (0.34, 2.06) | 0.81 (0.29, 2.23) |
| Remi10mg bid | 1.01 (0.13, 7.45) | 1.12 (0.29, 4.32) | 0.94 (0.24, 3.7) | 1.15 (0.35, 3.75) | 2.18 (0.45, 10.59) | 1.66 (0.43, 6.5) | 1.64 (0.36, 7.74) | 1.92 (0.71, 5.28) | 1.42 (0.43, 4.64) | 2.17 (0.61, 7.62) | 1.75 (0.65, 4.7) | 1.6 (0.6, 4.22) | 1.66 (0.64, 4.25) | 1.55 (0.36, 6.54) | 1.19 (0.42, 3.32) | 1.12 (0.45, 2.76) | 1.89 (0.31, 11.44) | 2.03 (0.71, 5.78) | 1.49 (0.55, 4.1) | Remi10mg bid | 2.16 (0.78, 6.19) | 1.24 (0.5, 3.09) | 1.2 (0.43, 3.35) |
| Remi10mg qd | 0.47 (0.06, 3.36) | 0.52 (0.13, 1.99) | 0.43 (0.11, 1.73) | 0.53 (0.16, 1.75) | 1 (0.2, 4.88) | 0.77 (0.2, 2.98) | 0.76 (0.16, 3.62) | 0.89 (0.32, 2.47) | 0.66 (0.19, 2.24) | 1 (0.27, 3.65) | 0.81 (0.29, 2.22) | 0.74 (0.27, 2) | 0.77 (0.28, 2.02) | 0.72 (0.16, 3.08) | 0.55 (0.19, 1.55) | 0.52 (0.2, 1.31) | 0.87 (0.14, 5.32) | 0.94 (0.32, 2.66) | 0.69 (0.24, 1.89) | 0.46 (0.16, 1.29) | Remi10mg qd | 0.57 (0.23, 1.47) | 0.56 (0.19, 1.57) |
| Remi25mg | 0.82 (0.12, 5.03) | 0.9 (0.3, 2.7) | 0.76 (0.24, 2.28) | 0.93 (0.38, 2.21) | 1.75 (0.46, 6.71) | 1.34 (0.45, 4.02) | 1.33 (0.35, 4.97) | 1.55 (0.82, 2.87) | 1.15 (0.46, 2.76) | 1.75 (0.64, 4.64) | 1.42 (0.76, 2.54) | 1.3 (0.7, 2.27) | 1.35 (0.77, 2.23) | 1.26 (0.37, 4.16) | 0.96 (0.48, 1.83) | 0.91 (0.57, 1.39) | 1.52 (0.3, 7.65) | 1.64 (0.63, 4.18) | 1.2 (0.49, 2.97) | 0.81 (0.32, 1.98) | 1.75 (0.68, 4.43) | Remi25mg | 0.97 (0.38, 2.44) |
| Remi35mg | 0.84 (0.11, 6.09) | 0.93 (0.24, 3.61) | 0.78 (0.2, 3.06) | 0.96 (0.29, 3.16) | 1.79 (0.38, 8.8) | 1.38 (0.36, 5.38) | 1.37 (0.29, 6.39) | 1.6 (0.58, 4.42) | 1.18 (0.35, 3.83) | 1.8 (0.5, 6.41) | 1.46 (0.53, 3.94) | 1.33 (0.49, 3.53) | 1.38 (0.52, 3.55) | 1.29 (0.3, 5.47) | 0.99 (0.34, 2.77) | 0.93 (0.37, 2.33) | 1.57 (0.25, 9.73) | 1.68 (0.6, 4.8) | 1.24 (0.45, 3.42) | 0.83 (0.3, 2.3) | 1.79 (0.64, 5.18) | 1.03 (0.41, 2.61) | Remi35mg |
| SUCRA | 0.6465 | 0.6539 | 0.7504 | 0.6633 | 0.3056 | 0.4211 | 0.4438 | 0.285 | 0.5291 | 0.268 | 0.3562 | 0.4283 | 0.3954 | 0.4667 | 0.6698 | 0.7292 | 0.3867 | 0.3093 | 0.4975 | 0.7402 | 0.2774 | 0.6433 | 0.6335 |

For adverse events (AEs), Network meta-analysis results are expressed as OR (95% CI). OR less than 1 indicate that the treatment specified in the row is safer than that specified in the column. The bottom row contains the Surface Under the Cumulative Ranking (SUCRA) value for the column-defining treatment. Both in efficacy and safety, a higher score on the lower surface under the cumulative ranking curve (SUCRA) indicate better efficacy and higher safety.

**eTable 9.** GRADE assessment by CINeMA: adverse events (AEs).

| Comparison | Number of studies | Within-study bias | Reporting bias | Indirectness | Imprecision | Heterogeneity | Incoherence | Confidence rating |
| --- | --- | --- | --- | --- | --- | --- | --- | --- |
| AZD1981 40mg tid | 1 | Some concerns | Low risk | No concerns | Major concerns | No concerns | No concerns | Low |
| Benralizumab 30mg qd | 1 | Some concerns | Low risk | No concerns | No concerns | No concerns | No concerns | Moderate |
| Benralizumab 60mg qd | 1 | Some concerns | Low risk | No concerns | No concerns | No concerns | No concerns | Moderate |
| Dupilumab | 1 | No concerns | Low risk | No concerns | No concerns | No concerns | No concerns | High |
| Fenebrutinib 150mg qd | 1 | Some concerns | Low risk | No concerns | Some concerns | No concerns | No concerns | Low |
| Fenebrutinib 200mg bid | 2 | Some concerns | Low risk | No concerns | No concerns | No concerns | No concerns | Moderate |
| Fenebrutinib 50mg qd | 1 | Some concerns | Low risk | No concerns | Some concerns | No concerns | No concerns | Low |
| Ligelizumab 120mg q4w | 2 | No concerns | Low risk | No concerns | No concerns | Some concerns | Some concerns | Low |
| Ligelizumab 240mg q4w | 1 | No concerns | Low risk | No concerns | No concerns | No concerns | No concerns | High |
| Ligelizumab 24mg q4w | 1 | No concerns | Low risk | No concerns | No concerns | No concerns | No concerns | High |
| Ligelizumab 72mg q4w | 3 | No concerns | Low risk | No concerns | No concerns | Some concerns | No concerns | Moderate |
| Omalizumab 150mg q4w | 4 | No concerns | Low risk | No concerns | No concerns | Some concerns | No concerns | Moderate |
| Omalizumab 300mg q4w | 11 | No concerns | Low risk | No concerns | No concerns | No concerns | No concerns | High |
| Omalizumab 600mg q4w | 1 | No concerns | Low risk | No concerns | No concerns | No concerns | No concerns | High |
| Omalizumab 75mg q4w | 3 | No concerns | Low risk | No concerns | No concerns | No concerns | No concerns | High |
| Quilizumab 450mg q4w | 1 | Some concerns | Low risk | No concerns | Major concerns | No concerns | No concerns | Low |
| Remibrutinib 100mg bid | 1 | No concerns | Low risk | No concerns | No concerns | No concerns | No concerns | High |
| Remibrutinib 100mg qd | 1 | No concerns | Low risk | No concerns | No concerns | No concerns | No concerns | High |
| Remibrutinib 10mg bid | 1 | No concerns | Low risk | No concerns | No concerns | No concerns | No concerns | High |
| Remibrutinib 10mg qd | 1 | No concerns | Low risk | No concerns | No concerns | No concerns | No concerns | High |
| Remibrutinib 25mg bid | 3 | No concerns | Low risk | No concerns | No concerns | No concerns | No concerns | High |
| Remibrutinib 35mg qd | 1 | No concerns | Low risk | No concerns | No concerns | No concerns | No concerns | High |

Minimum clinically important difference set to 1.2

**eFigure 7**. Network graphs for the incidence of serious adverse events (SAEs).


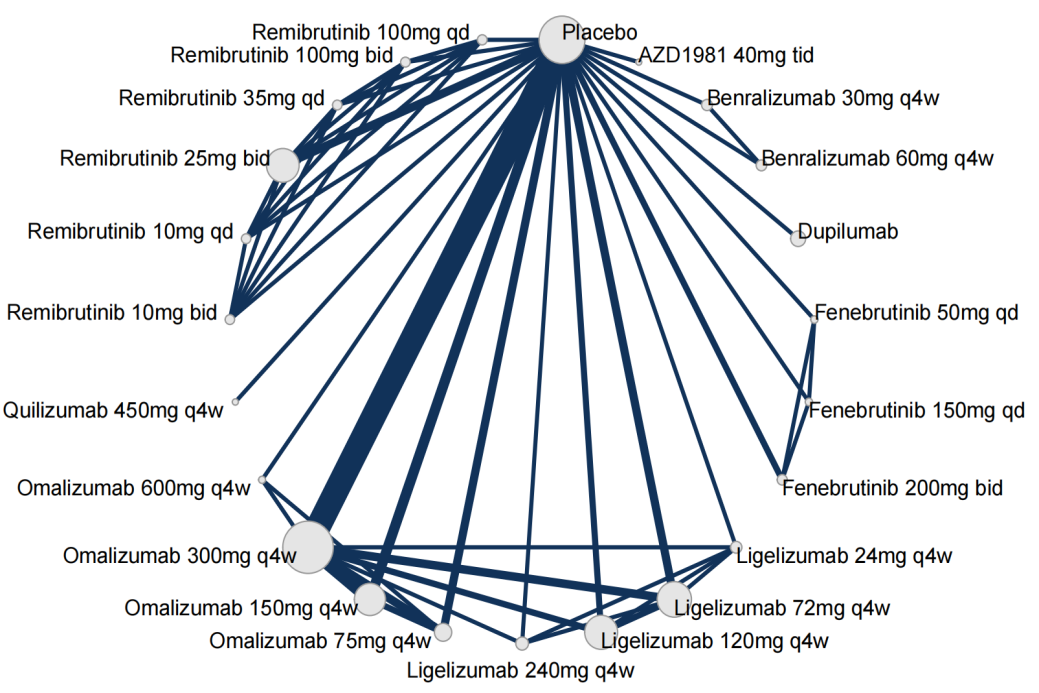


**eFigure 8.** Forest plot for the incidence of serious adverse events (SAEs).


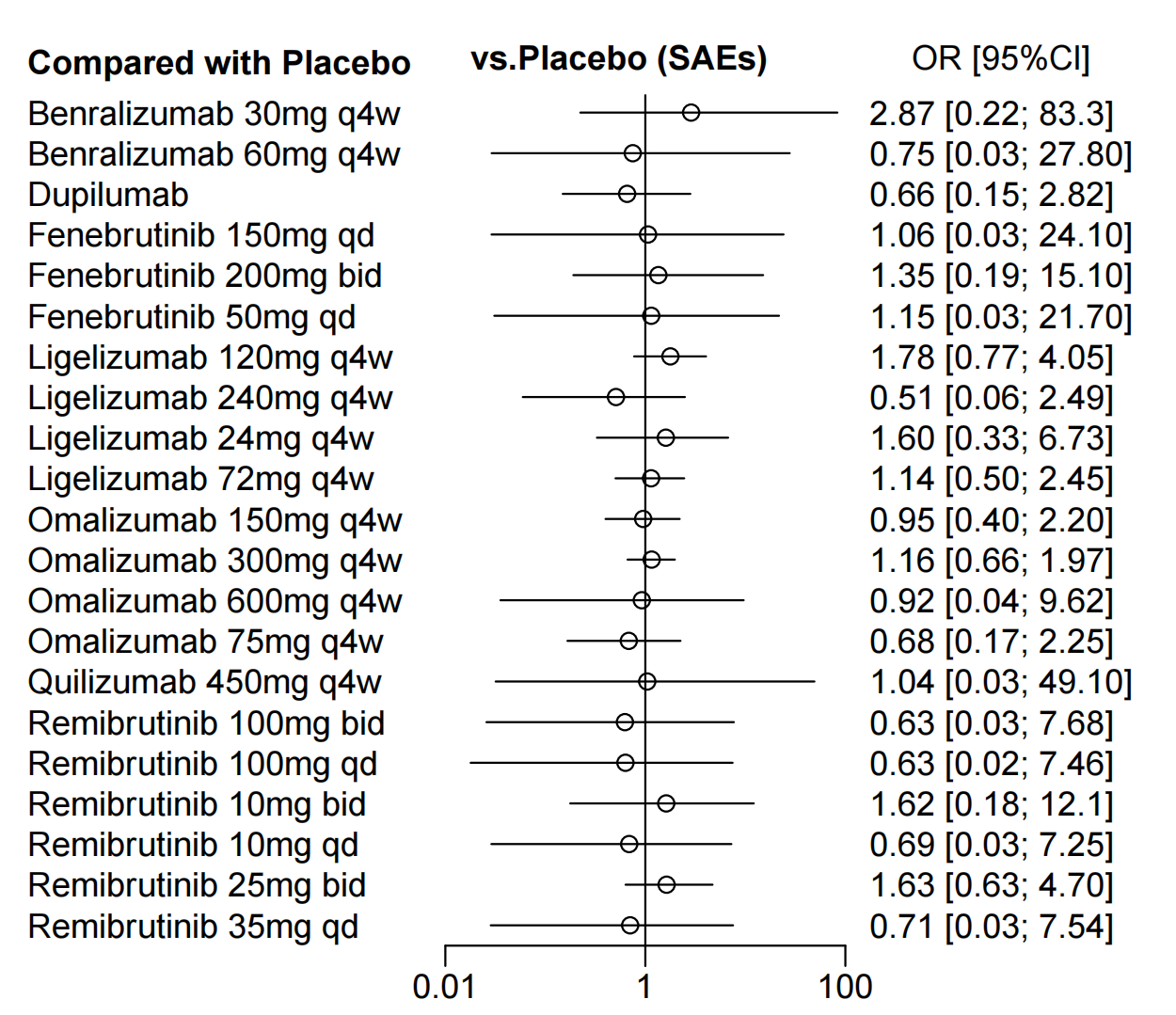


**eTable 10.** League table and the Surface Under the Cumulative Ranking (SUCRA): serious adverse events (SAEs).

|  | Benralizumab 30mg q4w | Benralizumab 60mg q4w | Dupilumab | Fenebrutinib 150mg qd | Fenebrutinib 200mg bid | Fenebrutinib 50mg qd | Ligelizumab 120mg q4w | Ligelizumab 240mg q4w | Ligelizumab 24mg q4w | Ligelizumab 72mg q4w | Omalizumab 150mg q4w | Omalizumab 300mg q4w | Omalizumab 600mg q4w | Omalizumab 75mg q4w | Placebo | Quilizumab 450mg q4w | Remibrutinib 100mg bid | Remibrutinib 100mg qd | Remibrutinib 10mg bid | Remibrutinib 10mg qd | Remibrutinib 25mg bid | Remibrutinib 35mg qd |
| --- | --- | --- | --- | --- | --- | --- | --- | --- | --- | --- | --- | --- | --- | --- | --- | --- | --- | --- | --- | --- | --- | --- |
| Benralizumab 30mg q4w | Benralizumab 30mg q4w | 0.27 (0.01, 2.67) | 0.22 (0.01, 4.51) | 0.36 (0, 18.98) | 0.44 (0.01, 12.88) | 0.37 (0, 13.83) | 0.62 (0.02, 9.05) | 0.18 (0, 3.32) | 0.55 (0.01, 9.63) | 0.4 (0.01, 5.56) | 0.35 (0.01, 4.8) | 0.4 (0.01, 5.36) | 0.28 (0, 10.59) | 0.24 (0.01, 3.71) | 0.35 (0.01, 4.47) | 0.37 (0, 31.23) | 0.21 (0, 8.43) | 0.21 (0, 7.17) | 0.53 (0.01, 16.35) | 0.22 (0, 8.59) | 0.58 (0.02, 9.26) | 0.22 (0, 8.59) |
| Benralizumab 60mg q4w | 3.77 (0.37, 67.15) | Benralizumab 60mg q4w | 0.91 (0.02, 32.01) | 1.39 (0.01, 111.48) | 1.84 (0.04, 79.41) | 1.42 (0.01, 109.63) | 2.4 (0.06, 67.78) | 0.64 (0.01, 25.11) | 2.18 (0.04, 73.42) | 1.54 (0.04, 41.5) | 1.27 (0.03, 37.68) | 1.55 (0.04, 39.47) | 1.17 (0.01, 69.47) | 0.91 (0.02, 30.64) | 1.34 (0.04, 34.44) | 1.44 (0.01, 233.67) | 0.82 (0.01, 52.4) | 0.78 (0.01, 40.29) | 2.17 (0.03, 104.45) | 0.81 (0.01, 47.54) | 2.22 (0.06, 67.83) | 0.89 (0.01, 51.31) |
| Dupilumab | 4.57 (0.22, 162.95) | 1.1 (0.03, 56.4) | Dupilumab | 1.58 (0.04, 46.71) | 2.04 (0.18, 33.3) | 1.68 (0.04, 42.02) | 2.71 (0.5, 15.14) | 0.77 (0.06, 6.99) | 2.46 (0.27, 18.87) | 1.73 (0.33, 8.96) | 1.46 (0.27, 8) | 1.75 (0.37, 8.3) | 1.32 (0.04, 23.17) | 1 (0.13, 7.21) | 1.52 (0.35, 6.7) | 1.56 (0.04, 116.2) | 1.03 (0.03, 17.55) | 0.94 (0.02, 17.17) | 2.4 (0.17, 31.88) | 1.01 (0.03, 17.14) | 2.5 (0.44, 15.18) | 1.06 (0.03, 17.06) |
| Fenebrutinib 150mg qd | 2.8 (0.05, 339.43) | 0.72 (0.01, 109.21) | 0.63 (0.02, 26.92) | Fenebrutinib 150mg qd | 1.31 (0.07, 44.15) | 1.05 (0.03, 39.51) | 1.64 (0.06, 64.89) | 0.46 (0.01, 27.81) | 1.44 (0.05, 73.1) | 1.07 (0.04, 39.86) | 0.94 (0.03, 35.04) | 1.09 (0.04, 39.58) | 0.78 (0.01, 61.06) | 0.66 (0.02, 30.2) | 0.94 (0.04, 34.53) | 1.06 (0.01, 225.51) | 0.58 (0.01, 41.42) | 0.57 (0, 42.31) | 1.48 (0.04, 81.84) | 0.64 (0.01, 41.23) | 1.58 (0.07, 65.03) | 0.62 (0.01, 48.91) |
| Fenebrutinib 200mg bid | 2.27 (0.08, 80.39) | 0.54 (0.01, 26.13) | 0.49 (0.03, 5.59) | 0.76 (0.02, 14.29) | Fenebrutinib 200mg bid | 0.8 (0.02, 13.95) | 1.31 (0.1, 11.25) | 0.35 (0.02, 4.57) | 1.11 (0.08, 14.38) | 0.83 (0.07, 6.92) | 0.72 (0.05, 5.88) | 0.84 (0.07, 6.59) | 0.62 (0.01, 15.05) | 0.5 (0.04, 5.25) | 0.74 (0.07, 5.25) | 0.81 (0.02, 49.11) | 0.44 (0.01, 12.97) | 0.46 (0.01, 10.47) | 1.14 (0.06, 23.04) | 0.47 (0.01, 12.42) | 1.2 (0.11, 11.91) | 0.47 (0.01, 11.6) |
| Fenebrutinib 50mg qd | 2.7 (0.07, 289.78) | 0.71 (0.01, 79.96) | 0.59 (0.02, 28.44) | 0.95 (0.03, 34.16) | 1.25 (0.07, 41.94) | Fenebrutinib 50mg qd | 1.62 (0.07, 62.45) | 0.43 (0.01, 24.49) | 1.42 (0.04, 71.83) | 1 (0.05, 39.63) | 0.86 (0.04, 33.73) | 1 (0.05, 37.64) | 0.73 (0.01, 59.02) | 0.61 (0.02, 22.74) | 0.87 (0.05, 32.4) | 1.02 (0.01, 164.88) | 0.54 (0.01, 56.21) | 0.55 (0, 46.68) | 1.46 (0.04, 103.89) | 0.57 (0.01, 57.63) | 1.48 (0.07, 67.62) | 0.59 (0.01, 44.87) |
| Ligelizumab 120mg q4w | 1.6 (0.11, 54.47) | 0.42 (0.01, 18.16) | 0.37 (0.07, 1.99) | 0.61 (0.02, 16.44) | 0.77 (0.09, 9.61) | 0.62 (0.02, 14.71) | Ligelizumab 120mg q4w | 0.28 (0.03, 1.57) | 0.88 (0.18, 4.19) | 0.64 (0.27, 1.4) | 0.55 (0.18, 1.53) | 0.65 (0.3, 1.38) | 0.49 (0.02, 6.08) | 0.38 (0.08, 1.5) | 0.56 (0.25, 1.3) | 0.6 (0.02, 29.27) | 0.34 (0.01, 5.12) | 0.35 (0.01, 4.63) | 0.92 (0.08, 8.2) | 0.4 (0.01, 4.76) | 0.91 (0.27, 3.68) | 0.4 (0.01, 5.87) |
| Ligelizumab 240mg q4w | 5.65 (0.3, 360.51) | 1.57 (0.04, 90.27) | 1.3 (0.14, 16.78) | 2.18 (0.04, 117.87) | 2.85 (0.22, 59.69) | 2.35 (0.04, 85.36) | 3.62 (0.64, 29.86) | Ligelizumab 240mg q4w | 3.26 (0.47, 26.06) | 2.31 (0.44, 17.83) | 1.95 (0.32, 16.75) | 2.34 (0.46, 18.01) | 1.81 (0.05, 39.61) | 1.39 (0.16, 14.7) | 1.97 (0.4, 16.82) | 2.25 (0.04, 157.92) | 1.24 (0.03, 38.78) | 1.22 (0.03, 32.84) | 3.19 (0.2, 61.02) | 1.38 (0.03, 35.95) | 3.25 (0.53, 32.73) | 1.45 (0.04, 32.63) |
| Ligelizumab 24mg q4w | 1.83 (0.1, 85.95) | 0.46 (0.01, 24.6) | 0.41 (0.05, 3.65) | 0.69 (0.01, 21.19) | 0.9 (0.07, 12.15) | 0.7 (0.01, 23.39) | 1.13 (0.24, 5.71) | 0.31 (0.04, 2.14) | Ligelizumab 24mg q4w | 0.72 (0.17, 3.4) | 0.6 (0.12, 3.21) | 0.73 (0.18, 3.34) | 0.56 (0.02, 10.46) | 0.42 (0.07, 2.77) | 0.62 (0.15, 3.05) | 0.65 (0.02, 40.01) | 0.39 (0.01, 8.11) | 0.38 (0.01, 7.67) | 1.02 (0.07, 13.83) | 0.43 (0.01, 7.52) | 1.01 (0.2, 7.31) | 0.43 (0.01, 7.71) |
| Ligelizumab 72mg q4w | 2.52 (0.18, 84.73) | 0.65 (0.02, 27.39) | 0.58 (0.11, 3.07) | 0.94 (0.03, 25.17) | 1.21 (0.14, 14.64) | 1 (0.03, 22.04) | 1.56 (0.72, 3.64) | 0.43 (0.06, 2.29) | 1.39 (0.29, 6.04) | Ligelizumab 72mg q4w | 0.85 (0.29, 2.43) | 1.01 (0.49, 2.13) | 0.78 (0.03, 9.59) | 0.6 (0.13, 2.31) | 0.88 (0.41, 1.99) | 0.92 (0.03, 45.59) | 0.54 (0.02, 8.18) | 0.54 (0.02, 7.35) | 1.41 (0.14, 13.13) | 0.62 (0.02, 7.61) | 1.43 (0.45, 5.73) | 0.61 (0.02, 8.13) |
| Omalizumab 150mg q4w | 2.88 (0.21, 103) | 0.79 (0.03, 32) | 0.68 (0.12, 3.77) | 1.06 (0.03, 29.72) | 1.38 (0.17, 18.55) | 1.16 (0.03, 26.03) | 1.83 (0.65, 5.57) | 0.51 (0.06, 3.15) | 1.66 (0.31, 8.02) | 1.18 (0.41, 3.45) | Omalizumab 150mg q4w | 1.2 (0.54, 2.78) | 0.9 (0.04, 11.44) | 0.72 (0.17, 2.46) | 1.05 (0.45, 2.5) | 1.08 (0.03, 53) | 0.64 (0.02, 9.57) | 0.64 (0.02, 8.48) | 1.72 (0.16, 15.06) | 0.72 (0.03, 8.83) | 1.74 (0.47, 6.71) | 0.72 (0.03, 9.96) |
| Omalizumab 300mg q4w | 2.48 (0.19, 78.25) | 0.65 (0.03, 25.93) | 0.57 (0.12, 2.71) | 0.92 (0.03, 23.39) | 1.18 (0.15, 13.51) | 1 (0.03, 20.58) | 1.55 (0.72, 3.38) | 0.43 (0.06, 2.16) | 1.38 (0.3, 5.67) | 0.99 (0.47, 2.03) | 0.83 (0.36, 1.87) | Omalizumab 300mg q4w | 0.8 (0.03, 8.47) | 0.6 (0.15, 1.9) | 0.87 (0.51, 1.51) | 0.91 (0.03, 43.78) | 0.53 (0.02, 7.15) | 0.54 (0.02, 6.7) | 1.4 (0.14, 11.58) | 0.61 (0.02, 6.96) | 1.42 (0.5, 4.81) | 0.6 (0.02, 7.33) |
| Omalizumab 600mg q4w | 3.56 (0.09, 343.77) | 0.86 (0.01, 117.5) | 0.76 (0.04, 25.22) | 1.28 (0.02, 87.24) | 1.6 (0.07, 71.42) | 1.37 (0.02, 84.11) | 2.04 (0.16, 56.13) | 0.55 (0.03, 20.26) | 1.8 (0.1, 57.37) | 1.28 (0.1, 36.09) | 1.11 (0.09, 28) | 1.26 (0.12, 33.06) | Omalizumab 600mg q4w | 0.77 (0.05, 19.71) | 1.08 (0.1, 28.12) | 1.3 (0.02, 174.89) | 0.72 (0.01, 45.39) | 0.77 (0.01, 44.28) | 1.83 (0.07, 87.72) | 0.8 (0.01, 44.3) | 1.84 (0.15, 55.29) | 0.77 (0.01, 44.29) |
| Omalizumab 75mg q4w | 4.2 (0.27, 155.29) | 1.1 (0.03, 53.54) | 1 (0.14, 7.56) | 1.51 (0.03, 43.66) | 1.99 (0.19, 24.69) | 1.63 (0.04, 44.89) | 2.61 (0.67, 12.66) | 0.72 (0.07, 6.11) | 2.39 (0.36, 15.12) | 1.66 (0.43, 7.74) | 1.4 (0.41, 5.96) | 1.66 (0.53, 6.75) | 1.3 (0.05, 18.51) | Omalizumab 75mg q4w | 1.46 (0.45, 6.02) | 1.65 (0.04, 95.89) | 0.93 (0.03, 17.65) | 0.91 (0.03, 15.46) | 2.46 (0.19, 30.38) | 0.95 (0.04, 16.92) | 2.42 (0.49, 14.36) | 0.98 (0.03, 17.17) |
| Placebo | 2.87 (0.22, 83.27) | 0.75 (0.03, 27.79) | 0.66 (0.15, 2.82) | 1.06 (0.03, 24.1) | 1.35 (0.19, 15.06) | 1.15 (0.03, 21.72) | 1.78 (0.77, 4.05) | 0.51 (0.06, 2.49) | 1.6 (0.33, 6.73) | 1.14 (0.5, 2.45) | 0.95 (0.4, 2.2) | 1.16 (0.66, 1.97) | 0.92 (0.04, 9.62) | 0.68 (0.17, 2.25) | Placebo | 1.04 (0.03, 49.09) | 0.62 (0.03, 7.68) | 0.63 (0.02, 7.46) | 1.62 (0.18, 12.15) | 0.69 (0.03, 7.25) | 1.63 (0.63, 4.7) | 0.71 (0.03, 7.54) |
| Quilizumab 450mg q4w | 2.69 (0.03, 378.93) | 0.7 (0, 114.94) | 0.64 (0.01, 25.07) | 0.95 (0, 85.27) | 1.24 (0.02, 63.24) | 0.98 (0.01, 83.49) | 1.68 (0.03, 61.46) | 0.45 (0.01, 22.49) | 1.53 (0.02, 64.64) | 1.09 (0.02, 37.77) | 0.92 (0.02, 32.61) | 1.1 (0.02, 36.21) | 0.77 (0.01, 52.39) | 0.61 (0.01, 27.59) | 0.96 (0.02, 31.42) | Quilizumab 450mg q4w | 0.62 (0, 38.6) | 0.55 (0, 35.72) | 1.6 (0.02, 70.86) | 0.61 (0, 38.94) | 1.56 (0.03, 58.1) | 0.63 (0.01, 33.11) |
| Remibrutinib 100mg bid | 4.84 (0.12, 417.22) | 1.22 (0.02, 131.6) | 0.97 (0.06, 36.31) | 1.72 (0.02, 125.01) | 2.26 (0.08, 84.13) | 1.85 (0.02, 134.31) | 2.92 (0.2, 81.45) | 0.81 (0.03, 31.2) | 2.54 (0.12, 79.42) | 1.84 (0.12, 51.32) | 1.55 (0.1, 45.37) | 1.87 (0.14, 49.19) | 1.4 (0.02, 74.09) | 1.08 (0.06, 32.74) | 1.6 (0.13, 38.83) | 1.61 (0.03, 236.16) | Remibrutinib 100mg bid | 0.98 (0.02, 32.86) | 2.48 (0.15, 81.4) | 1.08 (0.03, 37.61) | 2.54 (0.23, 66.84) | 1.1 (0.03, 33.74) |
| Remibrutinib 100mg qd | 4.8 (0.14, 793.79) | 1.28 (0.02, 185.25) | 1.06 (0.06, 45.56) | 1.75 (0.02, 212.59) | 2.17 (0.1, 124.15) | 1.83 (0.02, 207.92) | 2.87 (0.22, 97.8) | 0.82 (0.03, 32.85) | 2.61 (0.13, 92.82) | 1.85 (0.14, 60.44) | 1.57 (0.12, 58.46) | 1.84 (0.15, 57.18) | 1.3 (0.02, 126.18) | 1.1 (0.06, 39.23) | 1.58 (0.13, 55.84) | 1.81 (0.03, 306.49) | 1.02 (0.03, 48.39) | Remibrutinib 100mg qd | 2.6 (0.19, 86.88) | 1.11 (0.03, 54.77) | 2.6 (0.26, 94.68) | 1.13 (0.03, 51.62) |
| Remibrutinib 10mg bid | 1.89 (0.06, 98.6) | 0.46 (0.01, 31.28) | 0.42 (0.03, 5.97) | 0.67 (0.01, 22.65) | 0.87 (0.04, 17.9) | 0.69 (0.01, 25.88) | 1.09 (0.12, 12.05) | 0.31 (0.02, 5.02) | 0.98 (0.07, 15.19) | 0.71 (0.08, 7.32) | 0.58 (0.07, 6.35) | 0.71 (0.09, 7.37) | 0.55 (0.01, 13.83) | 0.41 (0.03, 5.24) | 0.62 (0.08, 5.64) | 0.63 (0.01, 57.44) | 0.4 (0.01, 6.6) | 0.38 (0.01, 5.29) | Remibrutinib 10mg bid | 0.42 (0.01, 5.82) | 1.04 (0.14, 9.5) | 0.42 (0.02, 6.64) |
| Remibrutinib 10mg qd | 4.52 (0.12, 341.59) | 1.24 (0.02, 140.94) | 0.99 (0.06, 34.29) | 1.55 (0.02, 111.63) | 2.14 (0.08, 91) | 1.77 (0.02, 113.83) | 2.53 (0.21, 75.85) | 0.73 (0.03, 30.58) | 2.34 (0.13, 88.64) | 1.62 (0.13, 46.62) | 1.39 (0.11, 39.59) | 1.63 (0.14, 43.15) | 1.25 (0.02, 75.87) | 1.06 (0.06, 27.55) | 1.45 (0.14, 34.67) | 1.63 (0.03, 247.62) | 0.93 (0.03, 31.97) | 0.9 (0.02, 31.58) | 2.37 (0.17, 73.13) | Remibrutinib 10mg qd | 2.44 (0.24, 60.87) | 1.01 (0.03, 33.1) |
| Remibrutinib 25mg bid | 1.73 (0.11, 58.43) | 0.45 (0.01, 18.08) | 0.4 (0.07, 2.27) | 0.63 (0.02, 14.24) | 0.84 (0.08, 8.9) | 0.68 (0.01, 14.39) | 1.1 (0.27, 3.75) | 0.31 (0.03, 1.87) | 0.99 (0.14, 5.06) | 0.7 (0.17, 2.23) | 0.57 (0.15, 2.12) | 0.71 (0.21, 2) | 0.54 (0.02, 6.82) | 0.41 (0.07, 2.04) | 0.61 (0.21, 1.58) | 0.64 (0.02, 32.87) | 0.39 (0.01, 4.38) | 0.38 (0.01, 3.9) | 0.96 (0.11, 7.28) | 0.41 (0.02, 4.1) | Remibrutinib 25mg bid | 0.42 (0.02, 4.34) |
| Remibrutinib 35mg qd | 4.57 (0.12, 392.13) | 1.13 (0.02, 132.36) | 0.95 (0.06, 32.07) | 1.62 (0.02, 78.98) | 2.13 (0.09, 77.07) | 1.69 (0.02, 100.29) | 2.52 (0.17, 71.07) | 0.69 (0.03, 28.22) | 2.34 (0.13, 92.5) | 1.64 (0.12, 41.58) | 1.39 (0.1, 38.17) | 1.66 (0.14, 43.92) | 1.3 (0.02, 75.65) | 1.02 (0.06, 30.46) | 1.42 (0.13, 35.18) | 1.58 (0.03, 199.94) | 0.91 (0.03, 33.04) | 0.88 (0.02, 31.17) | 2.39 (0.15, 63.09) | 0.99 (0.03, 30.24) | 2.38 (0.23, 61.95) | Remibrutinib 35mg qd |
| SUCRA | 0.2825 | 0.5771 | 0.6272 | 0.5011 | 0.4177 | 0.4863 | 0.2721 | 0.7023 | 0.3622 | 0.4639 | 0.5541 | 0.4527 | 0.5373 | 0.652 | 0.5246 | 0.4586 | 0.6212 | 0.6389 | 0.3521 | 0.5912 | 0.3084 | 0.6164 |

For adverse events (AEs), Network meta-analysis results are expressed as OR (95% CI). OR less than 1 indicate that the treatment specified in the row is safer than that specified in the column. The bottom row contains the Surface Under the Cumulative Ranking (SUCRA) value for the column-defining treatment. Both in efficacy and safety, a higher score on the lower surface under the cumulative ranking curve (SUCRA) indicate better efficacy and higher safety.

**eFigure 9**. Network graphs for the change from baseline in the weekly itch severity score (ISS7).


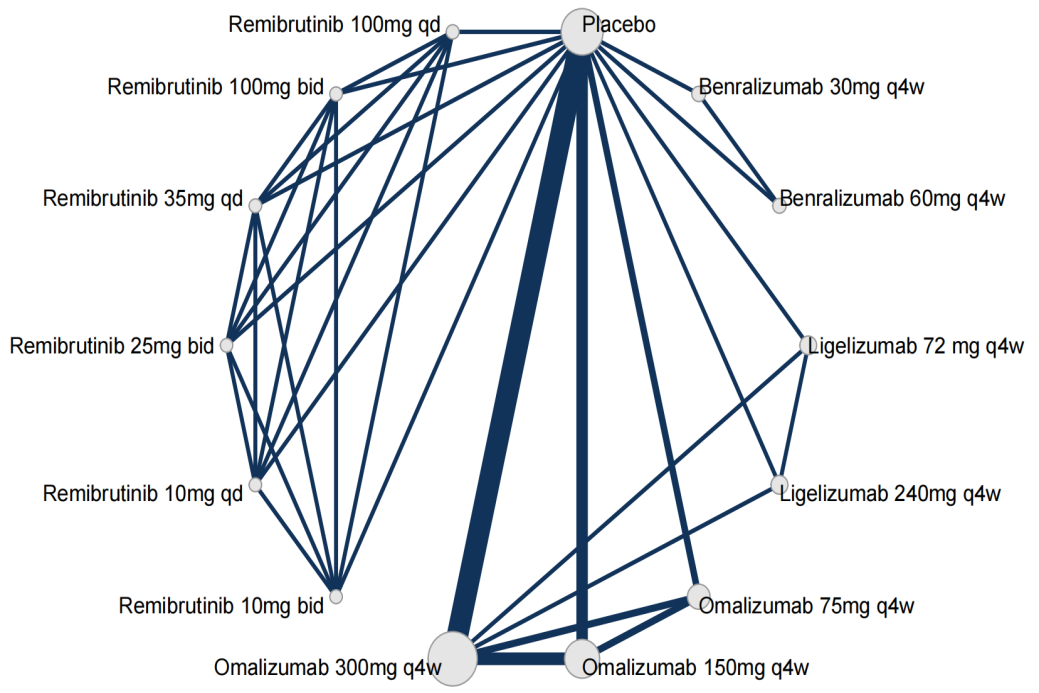


**eFigure 10**. Forest plot for the change from baseline in the weekly itch severity score (ISS7).


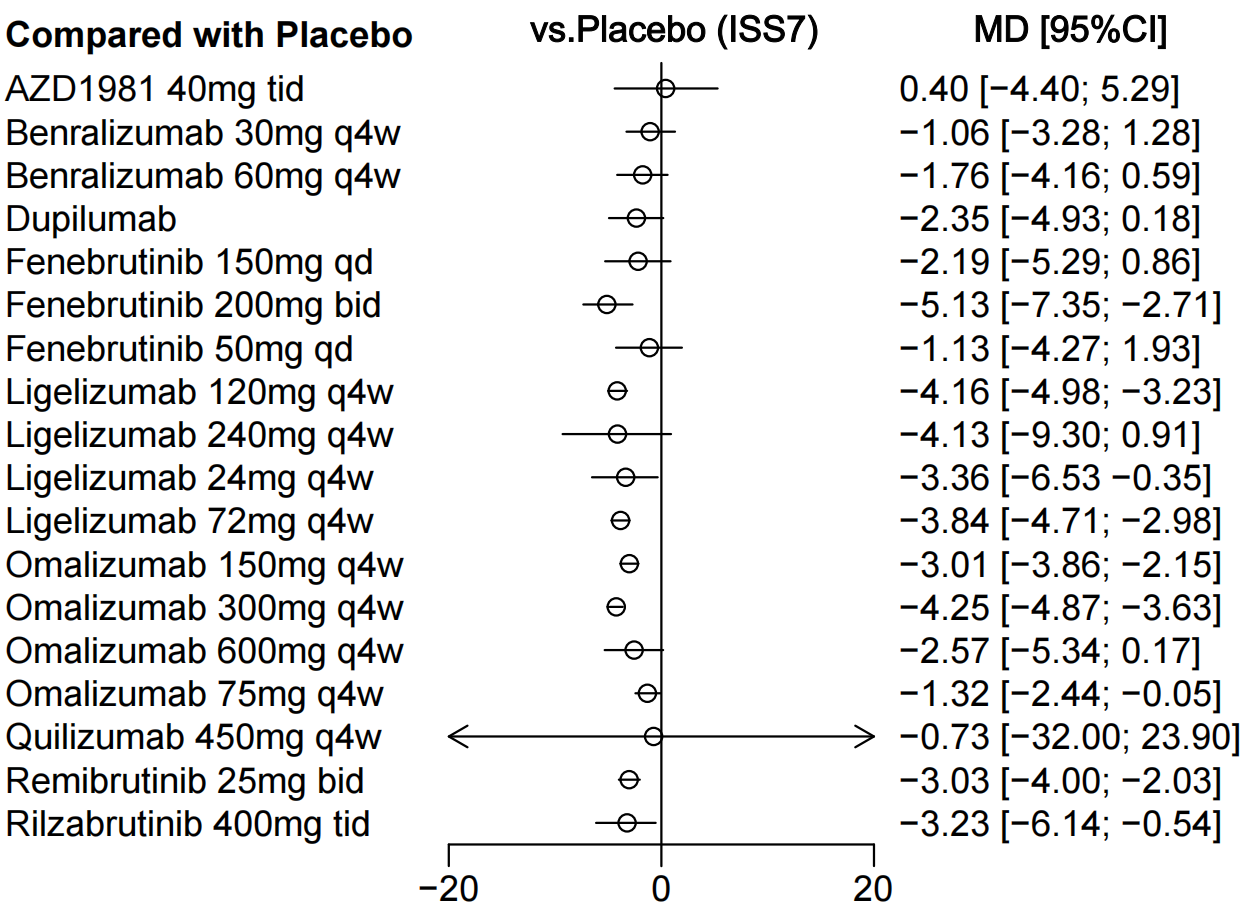


**eTable 11.** League table and the Surface Under the Cumulative Ranking (SUCRA): change from baseline in the weekly itch severity score (ISS7).

|  | AZD | Ben30mg | Ben60mg | Dup | Fen150mg | Fen200mg | Fen50mg | Lig120mg | Lig240mg | Lig24mg | Lig72mg | Oma150mg | Oma300mg | Oma600mg | Oma75mg | Placebo | Qui | Remi25mg bid | Ril |
| --- | --- | --- | --- | --- | --- | --- | --- | --- | --- | --- | --- | --- | --- | --- | --- | --- | --- | --- | --- |
| AZD | AZD | -1.56 (-6.68, 3.91) | -2.42 (-7.42, 3.09) | -2.88 (-8.09, 2.9) | -2.8 (-8.1, 3.26) | -5.58 (-10.67, -0.21) | -1.7 (-7.05, 4.13) | -4.7 (-9.26, 0.36) | -4.49 (-11.48, 2.95) | -3.88 (-9.66, 1.95) | -4.4 (-9, 0.64) | -3.51 (-8.15, 1.47) | -4.8 (-9.35, 0.19) | -3.24 (-8.52, 2.44) | -1.8 (-6.45, 3.2) | -0.54 (-5.07, 4.41) | -2.78 (-32.81, 22.46) | -3.5 (-8.21, 1.5) | -3.62 (-9.19, 1.91) |
| Ben30mg | 1.56 (-3.91, 6.68) | Ben30mg | -0.81 (-2.96, 1.41) | -1.27 (-4.87, 2.08) | -1.2 (-5.11, 2.53) | -3.99 (-7.3, -0.87) | -0.09 (-3.9, 3.67) | -3.09 (-5.59, -0.61) | -3.03 (-8.49, 2.4) | -2.3 (-6.5, 1.58) | -2.77 (-5.3, -0.3) | -1.93 (-4.49, 0.54) | -3.16 (-5.64, -0.78) | -1.61 (-5.3, 2.2) | -0.23 (-2.85, 2.4) | 1.07 (-1.31, 3.39) | -0.73 (-31.58, 23.55) | -1.94 (-4.52, 0.6) | -2.11 (-5.82, 1.49) |
| Ben60mg | 2.42 (-3.09, 7.42) | 0.81 (-1.41, 2.96) | Ben60mg | -0.52 (-4.04, 2.95) | -0.46 (-4.28, 3.44) | -3.21 (-6.46, 0.04) | 0.65 (-3.17, 4.6) | -2.29 (-4.78, 0.24) | -2.27 (-7.63, 3.68) | -1.55 (-5.64, 2.39) | -2 (-4.48, 0.52) | -1.15 (-3.67, 1.39) | -2.39 (-4.8, 0.09) | -0.85 (-4.42, 2.89) | 0.54 (-2.06, 3.2) | 1.85 (-0.46, 4.22) | -0.01 (-30.82, 24.63) | -1.16 (-3.66, 1.41) | -1.33 (-5.02, 2.19) |
| Dup | 2.88 (-2.9, 8.09) | 1.27 (-2.08, 4.87) | 0.52 (-2.95, 4.04) | Dup | 0.13 (-3.82, 3.96) | -2.73 (-6.01, 0.71) | 1.11 (-2.71, 5.33) | -1.82 (-4.4, 0.87) | -1.66 (-7.26, 3.83) | -1.07 (-5, 3) | -1.52 (-4.11, 1.23) | -0.68 (-3.29, 2.05) | -1.89 (-4.46, 0.76) | -0.27 (-4.08, 3.47) | 1.02 (-1.73, 3.94) | 2.33 (-0.12, 4.94) | 0.3 (-30.1, 25.23) | -0.67 (-3.34, 2.16) | -0.78 (-4.52, 2.81) |
| Fen150mg | 2.8 (-3.26, 8.1) | 1.2 (-2.53, 5.11) | 0.46 (-3.44, 4.28) | -0.13 (-3.96, 3.82) | Fen150mg | -2.79 (-5.92, 0.29) | 1.16 (-2.21, 4.32) | -1.9 (-5.12, 1.25) | -1.76 (-7.63, 4.74) | -1.06 (-5.89, 3.08) | -1.56 (-4.82, 1.55) | -0.69 (-3.93, 2.4) | -1.98 (-5.17, 1.21) | -0.41 (-4.5, 3.62) | 0.99 (-2.31, 4.21) | 2.28 (-0.87, 5.32) | 0.29 (-30.2, 24.32) | -0.71 (-3.99, 2.46) | -0.9 (-5, 3.59) |
| Fen200mg | 5.58 (0.21, 10.67) | 3.99 (0.87, 7.3) | 3.21 (-0.04, 6.46) | 2.73 (-0.71, 6.01) | 2.79 (-0.29, 5.92) | Fen200mg | 3.89 (0.69, 7.02) | 0.9 (-1.47, 3.37) | 1.02 (-4.54, 6.53) | 1.69 (-2.51, 5.32) | 1.2 (-1.18, 3.7) | 2.05 (-0.26, 4.49) | 0.82 (-1.49, 3.21) | 2.4 (-1.23, 5.85) | 3.74 (1.31, 6.33) | 5.06 (2.84, 7.38) | 3.21 (-27.68, 27.98) | 2.06 (-0.42, 4.54) | 1.89 (-1.71, 5.36) |
| Fen50mg | 1.7 (-4.13, 7.05) | 0.09 (-3.67, 3.9) | -0.65 (-4.6, 3.17) | -1.11 (-5.33, 2.71) | -1.16 (-4.32, 2.21) | -3.89 (-7.02, -0.69) | Fen50mg | -2.99 (-6.31, 0.27) | -2.92 (-8.81, 3.58) | -2.21 (-6.8, 2.05) | -2.69 (-6.04, 0.57) | -1.81 (-5.16, 1.46) | -3.04 (-6.4, 0.11) | -1.54 (-5.69, 2.58) | -0.12 (-3.57, 3.18) | 1.17 (-2.07, 4.27) | -0.71 (-31.73, 23.93) | -1.81 (-5.2, 1.42) | -2.01 (-6.22, 2.06) |
| Lig120mg | 4.7 (-0.36, 9.26) | 3.09 (0.61, 5.59) | 2.29 (-0.24, 4.78) | 1.82 (-0.87, 4.4) | 1.9 (-1.25, 5.12) | -0.9 (-3.37, 1.47) | 2.99 (-0.27, 6.31) | Lig120mg | 0.03 (-4.89, 5.05) | 0.74 (-2.48, 3.83) | 0.31 (-0.55, 1.12) | 1.16 (0.07, 2.23) | -0.07 (-0.92, 0.66) | 1.49 (-1.42, 4.42) | 2.84 (1.52, 4.23) | 4.16 (3.31, 5) | 2.2 (-28.49, 26.68) | 1.14 (-0.15, 2.49) | 0.97 (-1.92, 3.8) |
| Lig240mg | 4.49 (-2.95, 11.48) | 3.03 (-2.4, 8.49) | 2.27 (-3.68, 7.63) | 1.66 (-3.83, 7.26) | 1.76 (-4.74, 7.63) | -1.02 (-6.53, 4.54) | 2.92 (-3.58, 8.81) | -0.03 (-5.05, 4.89) | Lig240mg | 0.72 (-5.02, 6.27) | 0.25 (-4.78, 5.17) | 1.09 (-3.93, 6.09) | -0.14 (-5.13, 4.76) | 1.35 (-4.11, 7.14) | 2.79 (-2.35, 7.82) | 4.11 (-0.91, 8.99) | 1.51 (-28.72, 27.77) | 1.09 (-4.04, 6.08) | 0.85 (-4.9, 6.58) |
| Lig24mg | 3.88 (-1.95, 9.66) | 2.3 (-1.58, 6.5) | 1.55 (-2.39, 5.64) | 1.07 (-3, 5) | 1.06 (-3.08, 5.89) | -1.69 (-5.32, 2.51) | 2.21 (-2.05, 6.8) | -0.74 (-3.83, 2.48) | -0.72 (-6.27, 5.02) | Lig24mg | -0.45 (-3.54, 2.87) | 0.38 (-2.75, 3.81) | -0.83 (-3.93, 2.44) | 0.75 (-3.45, 4.95) | 2.08 (-1.16, 5.52) | 3.42 (0.31, 6.69) | 1.57 (-28.73, 27.01) | 0.38 (-2.87, 3.96) | 0.19 (-3.96, 4.64) |
| Lig72mg | 4.4 (-0.64, 9) | 2.77 (0.3, 5.3) | 2 (-0.52, 4.48) | 1.52 (-1.23, 4.11) | 1.56 (-1.55, 4.82) | -1.2 (-3.7, 1.18) | 2.69 (-0.57, 6.04) | -0.31 (-1.12, 0.55) | -0.25 (-5.17, 4.78) | 0.45 (-2.87, 3.54) | Lig72mg | 0.86 (-0.24, 1.96) | -0.37 (-1.19, 0.38) | 1.18 (-1.72, 4.13) | 2.53 (1.21, 3.93) | 3.84 (3.03, 4.73) | 1.94 (-28.81, 26.47) | 0.83 (-0.45, 2.19) | 0.67 (-2.2, 3.49) |
| Oma150mg | 3.51 (-1.47, 8.15) | 1.93 (-0.54, 4.49) | 1.15 (-1.39, 3.67) | 0.68 (-2.05, 3.29) | 0.69 (-2.4, 3.93) | -2.05 (-4.49, 0.26) | 1.81 (-1.46, 5.16) | -1.16 (-2.23, -0.07) | -1.09 (-6.09, 3.93) | -0.38 (-3.81, 2.75) | -0.86 (-1.96, 0.24) | Oma150mg | -1.23 (-2.1, -0.42) | 0.3 (-2.56, 3.25) | 1.68 (0.47, 2.98) | 2.99 (2.17, 3.85) | 1.07 (-29.67, 25.55) | -0.02 (-1.28, 1.29) | -0.19 (-3.07, 2.65) |
| Oma300mg | 4.8 (-0.19, 9.35) | 3.16 (0.78, 5.64) | 2.39 (-0.09, 4.8) | 1.89 (-0.76, 4.46) | 1.98 (-1.21, 5.17) | -0.82 (-3.21, 1.49) | 3.04 (-0.11, 6.4) | 0.07 (-0.66, 0.92) | 0.14 (-4.76, 5.13) | 0.83 (-2.44, 3.93) | 0.37 (-0.38, 1.19) | 1.23 (0.42, 2.1) | Oma300mg | 1.58 (-1.21, 4.47) | 2.92 (1.8, 4.18) | 4.23 (3.65, 4.88) | 2.23 (-28.48, 26.83) | 1.21 (0.08, 2.46) | 1.07 (-1.74, 3.81) |
| Oma600mg | 3.24 (-2.44, 8.52) | 1.61 (-2.2, 5.3) | 0.85 (-2.89, 4.42) | 0.27 (-3.47, 4.08) | 0.41 (-3.62, 4.5) | -2.4 (-5.85, 1.23) | 1.54 (-2.58, 5.69) | -1.49 (-4.42, 1.42) | -1.35 (-7.14, 4.11) | -0.75 (-4.95, 3.45) | -1.18 (-4.13, 1.72) | -0.3 (-3.25, 2.56) | -1.58 (-4.47, 1.21) | Oma600mg | 1.38 (-1.48, 4.27) | 2.68 (-0.2, 5.47) | 0.64 (-29.76, 25.59) | -0.31 (-3.39, 2.66) | -0.49 (-4.56, 3.38) |
| Oma75mg | 1.8 (-3.2, 6.45) | 0.23 (-2.4, 2.85) | -0.54 (-3.2, 2.06) | -1.02 (-3.94, 1.73) | -0.99 (-4.21, 2.31) | -3.74 (-6.33, -1.31) | 0.12 (-3.18, 3.57) | -2.84 (-4.23, -1.52) | -2.79 (-7.82, 2.35) | -2.08 (-5.52, 1.16) | -2.53 (-3.93, -1.21) | -1.68 (-2.98, -0.47) | -2.92 (-4.18, -1.8) | -1.38 (-4.27, 1.48) | Oma75mg | 1.3 (0.09, 2.47) | -0.57 (-31.33, 24.01) | -1.7 (-3.26, -0.18) | -1.89 (-4.91, 1.06) |
| Placebo | 0.54 (-4.41, 5.07) | -1.07 (-3.39, 1.31) | -1.85 (-4.22, 0.46) | -2.33 (-4.94, 0.12) | -2.28 (-5.32, 0.87) | -5.06 (-7.38, -2.84) | -1.17 (-4.27, 2.07) | -4.16 (-5, -3.31) | -4.11 (-8.99, 0.91) | -3.42 (-6.69, -0.31) | -3.84 (-4.73, -3.03) | -2.99 (-3.85, -2.17) | -4.23 (-4.88, -3.65) | -2.68 (-5.47, 0.2) | -1.3 (-2.47, -0.09) | Placebo | -1.86 (-32.67, 22.59) | -3.01 (-4.01, -1.98) | -3.16 (-5.95, -0.5) |
| Qui | 2.78 (-22.46, 32.81) | 0.73 (-23.55, 31.58) | 0.01 (-24.63, 30.82) | -0.3 (-25.23, 30.1) | -0.29 (-24.32, 30.2) | -3.21 (-27.98, 27.68) | 0.71 (-23.93, 31.73) | -2.2 (-26.68, 28.49) | -1.51 (-27.77, 28.72) | -1.57 (-27.01, 28.73) | -1.94 (-26.47, 28.81) | -1.07 (-25.55, 29.67) | -2.23 (-26.83, 28.48) | -0.64 (-25.59, 29.76) | 0.57 (-24.01, 31.33) | 1.86 (-22.59, 32.67) | Qui | -1.19 (-25.5, 29.58) | -1.08 (-25.55, 29.63) |
| Remi25mg bid | 3.5 (-1.5, 8.21) | 1.94 (-0.6, 4.52) | 1.16 (-1.41, 3.66) | 0.67 (-2.16, 3.34) | 0.71 (-2.46, 3.99) | -2.06 (-4.54, 0.42) | 1.81 (-1.42, 5.2) | -1.14 (-2.49, 0.15) | -1.09 (-6.08, 4.04) | -0.38 (-3.96, 2.87) | -0.83 (-2.19, 0.45) | 0.02 (-1.29, 1.28) | -1.21 (-2.46, -0.08) | 0.31 (-2.66, 3.39) | 1.7 (0.18, 3.26) | 3.01 (1.98, 4.01) | 1.19 (-29.58, 25.5) | Remi25mg bid | -0.18 (-3.14, 2.67) |
| Ril | 3.62 (-1.91, 9.19) | 2.11 (-1.49, 5.82) | 1.33 (-2.19, 5.02) | 0.78 (-2.81, 4.52) | 0.9 (-3.59, 5) | -1.89 (-5.36, 1.71) | 2.01 (-2.06, 6.22) | -0.97 (-3.8, 1.92) | -0.85 (-6.58, 4.9) | -0.19 (-4.64, 3.96) | -0.67 (-3.49, 2.2) | 0.19 (-2.65, 3.07) | -1.07 (-3.81, 1.74) | 0.49 (-3.38, 4.56) | 1.89 (-1.06, 4.91) | 3.16 (0.5, 5.95) | 1.08 (-29.63, 25.55) | 0.18 (-2.67, 3.14) | Ril |
| SUCRA | 0.1585 | 0.2435 | 0.368 | 0.4497 | 0.4469 | 0.8824 | 0.2801 | 0.7991 | 0.6762 | 0.6197 | 0.7217 | 0.5451 | 0.8205 | 0.5048 | 0.2634 | 0.0955 | 0.4833 | 0.5497 | 0.5918 |

For change from baseline in ISS7, Network meta-analysis results are expressed as MD (95% CI). The value of MD less than 0 indicate that the treatment specified in the row is more effective than that specified in the column. The bottom row contains the Surface Under the Cumulative Ranking (SUCRA) value for the column-defining treatment. Both in efficacy and safety, a higher score on the lower surface under the cumulative ranking curve (SUCRA) indicate better efficacy and higher safety.

**eFigure 11**. Network graphs for the change from baseline in the weekly hive severity score (HSS7).


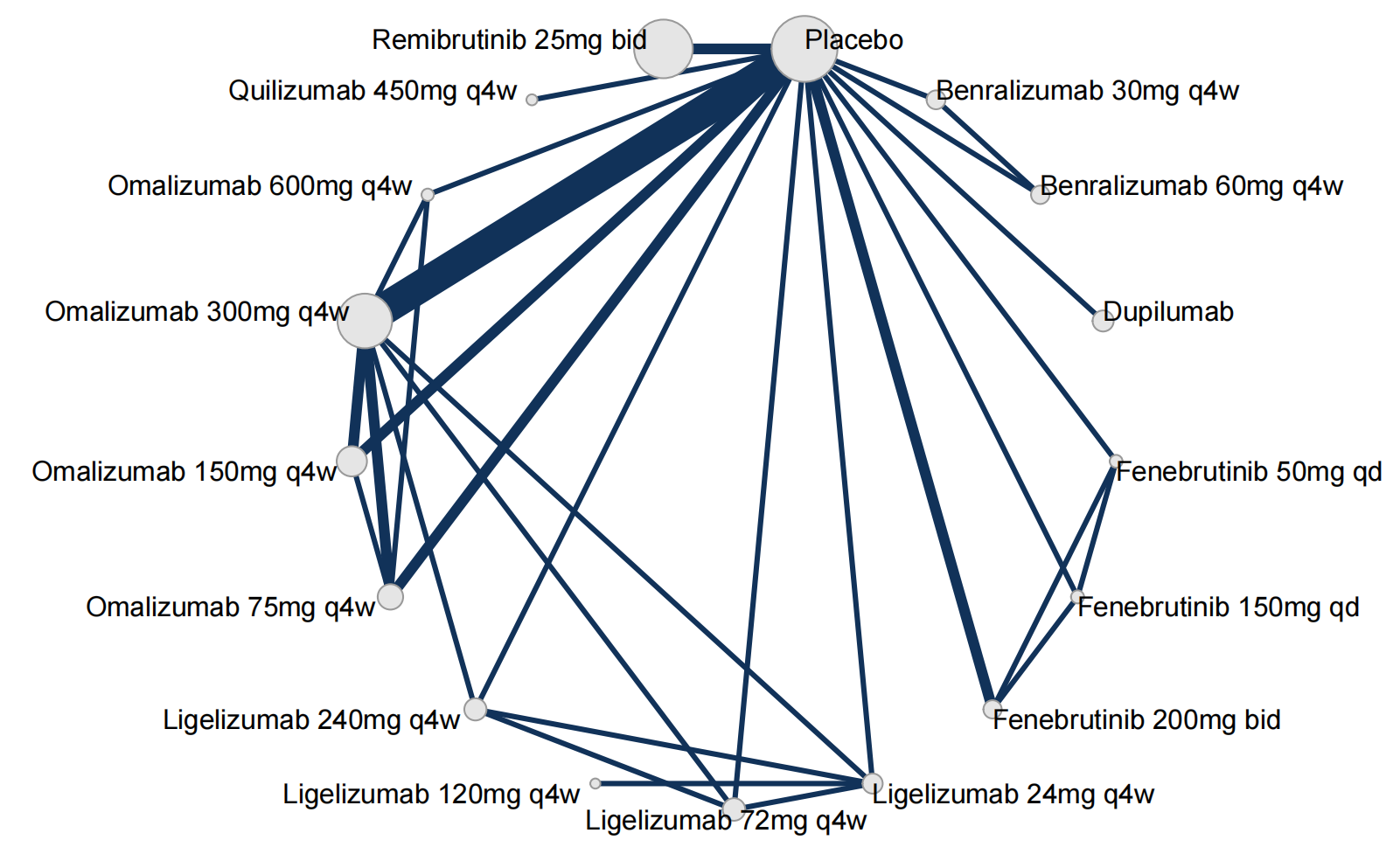


**eFigure 12**. Forest plot for the change from baseline in the weekly hive severity score (HSS7).


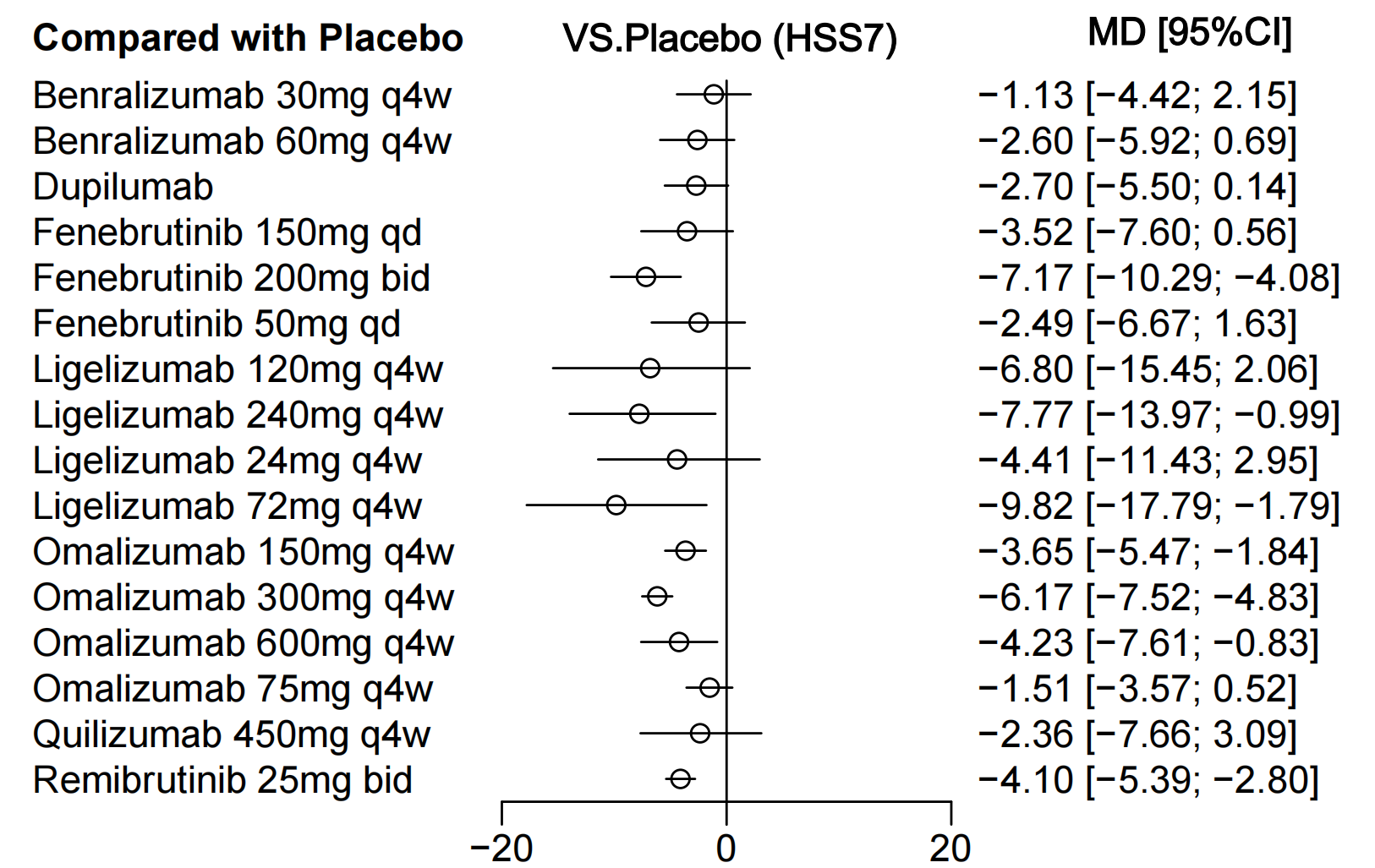


**eTable 12.** League table and the Surface Under the Cumulative Ranking (SUCRA): change from baseline in the weekly hive severity score (HSS7).

|  | Ben30mg | Ben60mg | Dup | Fen150mg | Fen200mg | Fen50mg | Lig120mg | Lig240mg | Lig24mg | Lig72mg | Oma150mg | Oma300mg | Oma600mg | Oma75mg | Placebo | Qui | Remi25mg bid |
| --- | --- | --- | --- | --- | --- | --- | --- | --- | --- | --- | --- | --- | --- | --- | --- | --- | --- |
| Ben30mg | Ben30mg | -1.45 (-4.5, 1.67) | -1.5 (-5.9, 2.88) | -2.3 (-7.53, 2.85) | -5.97 (-10.48, -1.55) | -1.33 (-6.61, 3.93) | -4.93 (-14.5, 4.75) | -6.36 (-13.52, 0.97) | -2.3 (-10.52, 5.88) | -8.41 (-17.37, 0.18) | -2.45 (-6.23, 1.29) | -4.98 (-8.62, -1.43) | -3.01 (-7.73, 1.64) | -0.27 (-4.19, 3.6) | 1.2 (-2.15, 4.51) | -1.15 (-7.77, 5.33) | -2.91 (-6.49, 0.61) |
| Ben60mg | 1.45 (-1.67, 4.5) | Ben60mg | -0.05 (-4.43, 4.29) | -0.88 (-6.07, 4.39) | -4.55 (-9.03, -0.02) | 0.09 (-5.16, 5.39) | -3.48 (-13.08, 6.1) | -4.86 (-12.13, 2.48) | -0.86 (-9.15, 7.25) | -6.95 (-15.86, 1.54) | -1 (-4.83, 2.73) | -3.54 (-7.16, 0.01) | -1.57 (-6.24, 3.12) | 1.17 (-2.78, 5.03) | 2.63 (-0.71, 5.95) | 0.29 (-6.25, 6.81) | -1.47 (-5.02, 2.06) |
| Dup | 1.5 (-2.88, 5.9) | 0.05 (-4.29, 4.43) | Dup | -0.82 (-5.74, 4.14) | -4.48 (-8.7, -0.27) | 0.15 (-4.86, 5.19) | -3.4 (-12.86, 6.06) | -4.84 (-11.9, 2.28) | -0.8 (-8.89, 7.13) | -6.84 (-15.69, 1.33) | -0.94 (-4.31, 2.37) | -3.49 (-6.63, -0.4) | -1.51 (-5.91, 2.86) | 1.23 (-2.3, 4.69) | 2.69 (-0.14, 5.54) | 0.34 (-5.97, 6.55) | -1.41 (-4.53, 1.69) |
| Fen150mg | 2.3 (-2.85, 7.53) | 0.88 (-4.39, 6.07) | 0.82 (-4.14, 5.74) | Fen150mg | -3.67 (-7.73, 0.4) | 0.98 (-3.41, 5.33) | -2.56 (-12.56, 7.38) | -3.96 (-11.69, 3.6) | 0.01 (-8.67, 8.7) | -6.06 (-15.47, 2.73) | -0.12 (-4.63, 4.25) | -2.66 (-7, 1.53) | -0.7 (-5.97, 4.53) | 2.04 (-2.5, 6.53) | 3.5 (-0.58, 7.49) | 1.13 (-5.8, 8.12) | -0.62 (-4.87, 3.57) |
| Fen200mg | 5.97 (1.55, 10.48) | 4.55 (0.02, 9.03) | 4.48 (0.27, 8.7) | 3.67 (-0.4, 7.73) | Fen200mg | 4.63 (0.52, 8.74) | 1.05 (-8.58, 10.74) | -0.38 (-7.53, 6.86) | 3.71 (-4.43, 11.83) | -2.41 (-11.36, 6.03) | 3.54 (-0.08, 7.04) | 1 (-2.41, 4.31) | 2.98 (-1.55, 7.49) | 5.71 (2.01, 9.39) | 7.18 (4.04, 10.22) | 4.83 (-1.59, 11.19) | 3.08 (-0.31, 6.35) |
| Fen50mg | 1.33 (-3.93, 6.61) | -0.09 (-5.39, 5.16) | -0.15 (-5.19, 4.86) | -0.98 (-5.33, 3.41) | -4.63 (-8.74, -0.52) | Fen50mg | -3.55 (-13.76, 6.36) | -4.96 (-12.85, 2.66) | -0.92 (-9.82, 7.48) | -7.01 (-16.44, 1.81) | -1.11 (-5.69, 3.4) | -3.64 (-8.03, 0.66) | -1.66 (-7.01, 3.59) | 1.06 (-3.56, 5.67) | 2.53 (-1.62, 6.67) | 0.22 (-6.91, 7.13) | -1.58 (-5.94, 2.76) |
| Lig120mg | 4.93 (-4.75, 14.5) | 3.48 (-6.1, 13.08) | 3.4 (-6.06, 12.86) | 2.56 (-7.38, 12.56) | -1.05 (-10.74, 8.58) | 3.55 (-6.36, 13.76) | Lig120mg | -1.38 (-10.88, 8.13) | 2.63 (-2.44, 7.61) | -3.51 (-14.5, 7.24) | 2.47 (-6.85, 11.67) | -0.06 (-9.31, 8.98) | 1.9 (-7.8, 11.63) | 4.65 (-4.75, 13.92) | 6.12 (-3.05, 15.2) | 3.77 (-7.1, 14.34) | 2 (-7.26, 11.19) |
| Lig240mg | 6.36 (-0.97, 13.52) | 4.86 (-2.48, 12.13) | 4.84 (-2.28, 11.9) | 3.96 (-3.6, 11.69) | 0.38 (-6.86, 7.53) | 4.96 (-2.66, 12.85) | 1.38 (-8.13, 10.88) | Lig240mg | 4.03 (-3.88, 11.82) | -2.19 (-10.66, 6.65) | 3.93 (-2.86, 10.55) | 1.39 (-5.3, 7.84) | 3.35 (-4.03, 10.55) | 6.05 (-0.75, 12.86) | 7.54 (0.92, 14) | 5.16 (-3.5, 13.55) | 3.44 (-3.28, 10.03) |
| Lig24mg | 2.3 (-5.88, 10.52) | 0.86 (-7.25, 9.15) | 0.8 (-7.13, 8.89) | -0.01 (-8.7, 8.67) | -3.71 (-11.83, 4.43) | 0.92 (-7.48, 9.82) | -2.63 (-7.61, 2.44) | -4.03 (-11.82, 3.88) | Lig24mg | -6.11 (-15.79, 3.36) | -0.13 (-7.84, 7.55) | -2.64 (-10.18, 4.93) | -0.68 (-8.95, 7.56) | 2.08 (-5.72, 9.86) | 3.52 (-3.88, 11.18) | 1.18 (-8.46, 10.37) | -0.58 (-8.13, 7.19) |
| Lig72mg | 8.41 (-0.18, 17.37) | 6.95 (-1.54, 15.86) | 6.84 (-1.33, 15.69) | 6.06 (-2.73, 15.47) | 2.41 (-6.03, 11.36) | 7.01 (-1.81, 16.44) | 3.51 (-7.24, 14.5) | 2.19 (-6.65, 10.66) | 6.11 (-3.36, 15.79) | Lig72mg | 5.86 (-1.96, 14.46) | 3.32 (-4.33, 11.72) | 5.31 (-2.93, 14.35) | 8.03 (0.23, 16.62) | 9.51 (1.85, 17.87) | 7.18 (-2.46, 17.18) | 5.42 (-2.34, 13.85) |
| Oma150mg | 2.45 (-1.29, 6.23) | 1 (-2.73, 4.83) | 0.94 (-2.37, 4.31) | 0.12 (-4.25, 4.63) | -3.54 (-7.04, 0.08) | 1.11 (-3.4, 5.69) | -2.47 (-11.67, 6.85) | -3.93 (-10.55, 2.86) | 0.13 (-7.55, 7.84) | -5.86 (-14.46, 1.96) | Oma150mg | -2.53 (-4.36, -0.74) | -0.56 (-4.22, 3.13) | 2.18 (-0.11, 4.47) | 3.65 (1.84, 5.43) | 1.3 (-4.66, 7.15) | -0.46 (-2.64, 1.72) |
| Oma300mg | 4.98 (1.43, 8.62) | 3.54 (-0.01, 7.16) | 3.49 (0.4, 6.63) | 2.66 (-1.53, 7) | -1 (-4.31, 2.41) | 3.64 (-0.66, 8.03) | 0.06 (-8.98, 9.31) | -1.39 (-7.84, 5.3) | 2.64 (-4.93, 10.18) | -3.32 (-11.72, 4.33) | 2.53 (0.74, 4.36) | Oma300mg | 1.98 (-1.42, 5.39) | 4.71 (2.62, 6.78) | 6.17 (4.83, 7.56) | 3.82 (-1.95, 9.51) | 2.06 (0.25, 3.94) |
| Oma600mg | 3.01 (-1.64, 7.73) | 1.57 (-3.12, 6.24) | 1.51 (-2.86, 5.91) | 0.7 (-4.53, 5.97) | -2.98 (-7.49, 1.55) | 1.66 (-3.59, 7.01) | -1.9 (-11.63, 7.8) | -3.35 (-10.55, 4.03) | 0.68 (-7.56, 8.95) | -5.31 (-14.35, 2.93) | 0.56 (-3.13, 4.22) | -1.98 (-5.39, 1.42) | Oma600mg | 2.73 (-0.82, 6.3) | 4.2 (0.86, 7.57) | 1.86 (-4.73, 8.32) | 0.09 (-3.46, 3.68) |
| Oma75mg | 0.27 (-3.6, 4.19) | -1.17 (-5.03, 2.78) | -1.23 (-4.69, 2.3) | -2.04 (-6.53, 2.5) | -5.71 (-9.39, -2.01) | -1.06 (-5.67, 3.56) | -4.65 (-13.92, 4.75) | -6.05 (-12.86, 0.75) | -2.08 (-9.86, 5.72) | -8.03 (-16.62, -0.23) | -2.18 (-4.47, 0.11) | -4.71 (-6.78, -2.62) | -2.73 (-6.3, 0.82) | Oma75mg | 1.47 (-0.55, 3.52) | -0.87 (-6.97, 5.11) | -2.63 (-5.01, -0.24) |
| Placebo | -1.2 (-4.51, 2.15) | -2.63 (-5.95, 0.71) | -2.69 (-5.54, 0.14) | -3.5 (-7.49, 0.58) | -7.18 (-10.22, -4.04) | -2.53 (-6.67, 1.62) | -6.12 (-15.2, 3.05) | -7.54 (-14, -0.92) | -3.52 (-11.18, 3.88) | -9.51 (-17.87, -1.85) | -3.65 (-5.43, -1.84) | -6.17 (-7.56, -4.83) | -4.2 (-7.57, -0.86) | -1.47 (-3.52, 0.55) | Placebo | -2.35 (-8.01, 3.2) | -4.11 (-5.39, -2.82) |
| Qui | 1.15 (-5.33, 7.77) | -0.29 (-6.81, 6.25) | -0.34 (-6.55, 5.97) | -1.13 (-8.12, 5.8) | -4.83 (-11.19, 1.59) | -0.22 (-7.13, 6.91) | -3.77 (-14.34, 7.1) | -5.16 (-13.55, 3.5) | -1.18 (-10.37, 8.46) | -7.18 (-17.18, 2.46) | -1.3 (-7.15, 4.66) | -3.82 (-9.51, 1.95) | -1.86 (-8.32, 4.73) | 0.87 (-5.11, 6.97) | 2.35 (-3.2, 8.01) | Qui | -1.77 (-7.45, 4.04) |
| Remi25mg bid | 2.91 (-0.61, 6.49) | 1.47 (-2.06, 5.02) | 1.41 (-1.69, 4.53) | 0.62 (-3.57, 4.87) | -3.08 (-6.35, 0.31) | 1.58 (-2.76, 5.94) | -2 (-11.19, 7.26) | -3.44 (-10.03, 3.28) | 0.58 (-7.19, 8.13) | -5.42 (-13.85, 2.34) | 0.46 (-1.72, 2.64) | -2.06 (-3.94, -0.25) | -0.09 (-3.68, 3.46) | 2.63 (0.24, 5.01) | 4.11 (2.82, 5.39) | 1.77 (-4.04, 7.45) | Remi25mg bid |
| SUCRA | 0.203 | 0.3755 | 0.373 | 0.475 | 0.8462 | 0.3612 | 0.6721 | 0.802 | 0.4539 | 0.8853 | 0.4951 | 0.79 | 0.5587 | 0.2208 | 0.0665 | 0.3582 | 0.5634 |

For change from baseline in HSS7, Network meta-analysis results are expressed as MD (95% CI). The value of MD less than 0 indicate that the treatment specified in the row is more effective than that specified in the column. The bottom row contains the Surface Under the Cumulative Ranking (SUCRA) value for the column-defining treatment. Both in efficacy and safety, a higher score on the lower surface under the cumulative ranking curve (SUCRA) indicate better efficacy and higher safety.

**eFigure 13**. Network graphs for the change from baseline in the dermatology life quality index (DLQI).


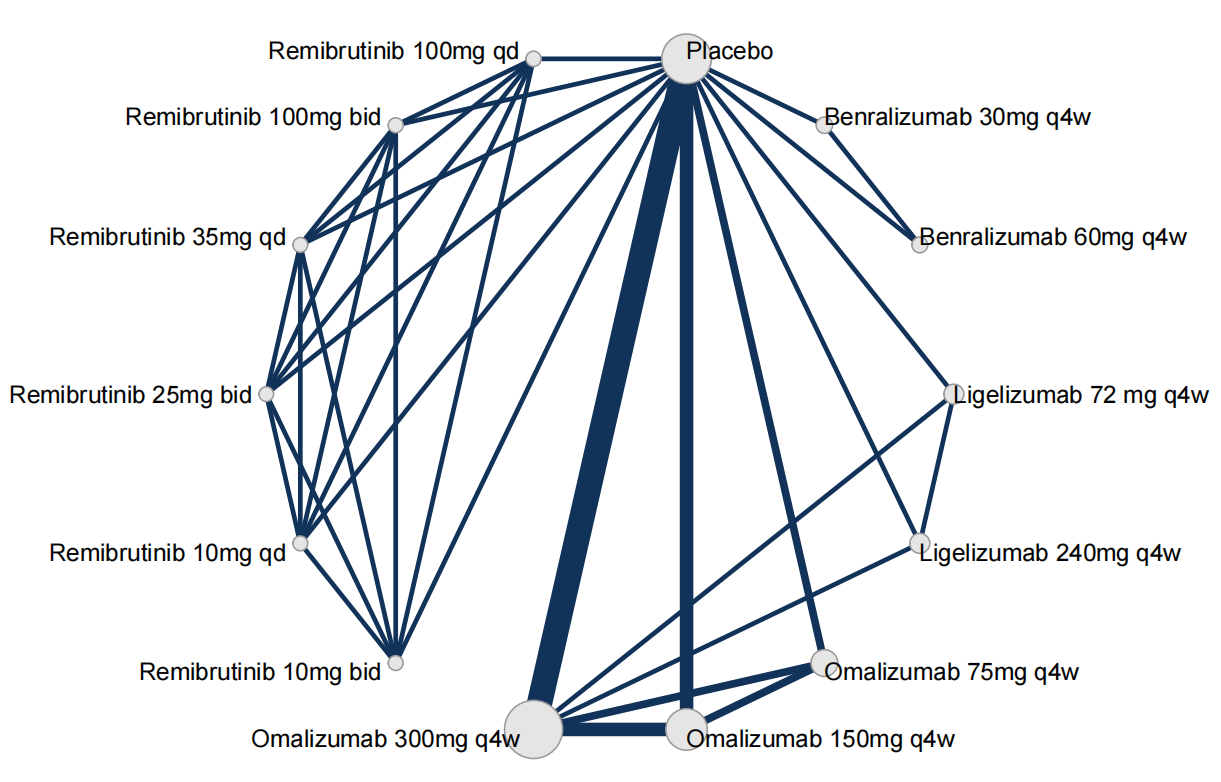


**eFigure 14.** Forest Plot for the change from baseline in the dermatology life quality index (DLQI).


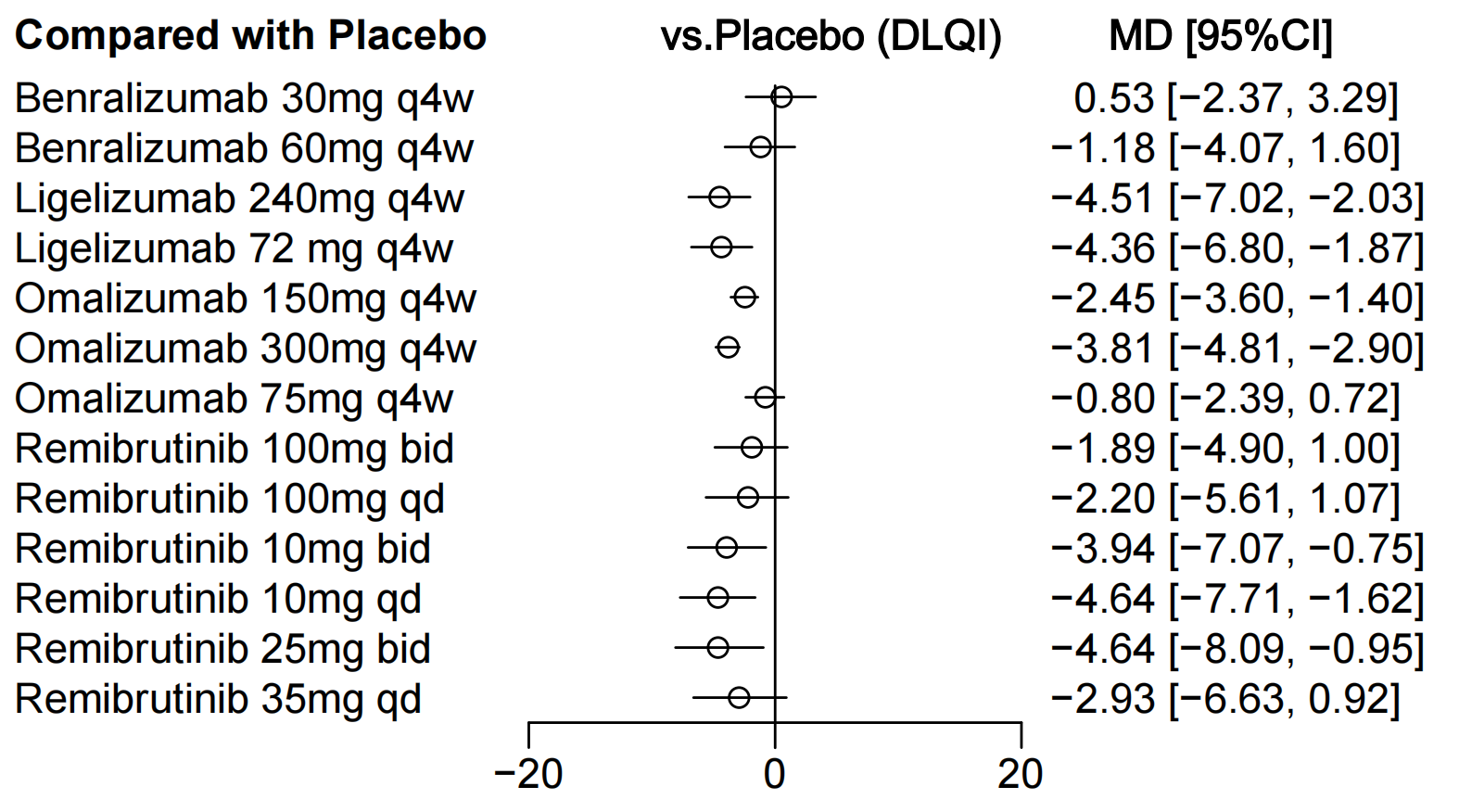


**eTable 13.** League table and the Surface Under the Cumulative Ranking (SUCRA): change from baseline in the dermatology life quality index (DLQI).

|  | Ben30mg | Ben60mg | Lig240mg | Lig72mg | Oma150mg | Oma300mg | Oma75mg | Placebo | Remi100mg bid | Remi100mg qd | Remi10mg bid | Remi10mg qd | Remi25mg bid | Remi35mg qd |
| --- | --- | --- | --- | --- | --- | --- | --- | --- | --- | --- | --- | --- | --- | --- |
| Ben30mg | Ben30mg | -1.79 (-4.44, 0.87) | -5.07 (-8.76, -1.27) | -4.92 (-8.54, -1.14) | -3.02 (-5.99, 0.01) | -4.3 (-7.3, -1.39) | -1.32 (-4.54, 1.83) | -0.52 (-3.31, 2.3) | -2.35 (-6.51, 1.62) | -2.69 (-7.05, 1.58) | -4.39 (-8.48, -0.19) | -5.13 (-9.36, -1.14) | -5.17 (-9.75, -0.62) | -3.49 (-8.12, 1.26) |
| Ben60mg | 1.79 (-0.87, 4.44) | Ben60mg | -3.25 (-7.01, 0.52) | -3.11 (-6.81, 0.64) | -1.21 (-4.19, 1.88) | -2.54 (-5.51, 0.45) | 0.47 (-2.72, 3.7) | 1.25 (-1.52, 4.15) | -0.58 (-4.65, 3.49) | -0.9 (-5.21, 3.47) | -2.56 (-6.74, 1.59) | -3.36 (-7.59, 0.74) | -3.35 (-8.01, 1.22) | -1.69 (-6.39, 3.09) |
| Lig240mg | 5.07 (1.27, 8.76) | 3.25 (-0.52, 7.01) | Lig240mg | 0.2 (-2.52, 2.79) | 2.09 (-0.57, 4.63) | 0.75 (-1.78, 3.08) | 3.76 (0.87, 6.49) | 4.56 (2.02, 7.03) | 2.74 (-1.28, 6.43) | 2.41 (-1.92, 6.43) | 0.78 (-3.36, 4.61) | -0.08 (-4.09, 3.73) | -0.04 (-4.63, 4.31) | 1.58 (-2.9, 5.93) |
| Lig72mg | 4.92 (1.14, 8.54) | 3.11 (-0.64, 6.81) | -0.2 (-2.79, 2.52) | Lig72mg | 1.89 (-0.62, 4.42) | 0.56 (-1.83, 2.91) | 3.58 (0.76, 6.28) | 4.38 (1.97, 6.77) | 2.55 (-1.4, 6.26) | 2.17 (-1.94, 6.34) | 0.51 (-3.42, 4.43) | -0.28 (-4.28, 3.56) | -0.23 (-4.73, 4.12) | 1.39 (-3.06, 5.83) |
| Oma150mg | 3.02 (-0.01, 5.99) | 1.21 (-1.88, 4.19) | -2.09 (-4.63, 0.57) | -1.89 (-4.42, 0.62) | Oma150mg | -1.33 (-2.48, -0.3) | 1.68 (0.08, 3.21) | 2.48 (1.38, 3.56) | 0.64 (-2.69, 3.83) | 0.29 (-3.25, 3.79) | -1.37 (-4.73, 1.96) | -2.15 (-5.5, 0.97) | -2.15 (-6.02, 1.77) | -0.46 (-4.34, 3.36) |
| Oma300mg | 4.3 (1.39, 7.3) | 2.54 (-0.45, 5.51) | -0.75 (-3.08, 1.78) | -0.56 (-2.91, 1.83) | 1.33 (0.3, 2.48) | Oma300mg | 2.99 (1.46, 4.58) | 3.8 (2.88, 4.8) | 1.98 (-1.24, 5.06) | 1.61 (-1.8, 5.08) | -0.04 (-3.26, 3.23) | -0.82 (-4.06, 2.29) | -0.82 (-4.62, 3.05) | 0.86 (-2.94, 4.69) |
| Oma75mg | 1.32 (-1.83, 4.54) | -0.47 (-3.7, 2.72) | -3.76 (-6.49, -0.87) | -3.58 (-6.28, -0.76) | -1.68 (-3.21, -0.08) | -2.99 (-4.58, -1.46) | Oma75mg | 0.81 (-0.72, 2.38) | -1.02 (-4.52, 2.27) | -1.4 (-5.08, 2.33) | -3.05 (-6.52, 0.46) | -3.81 (-7.34, -0.46) | -3.82 (-7.87, 0.25) | -2.13 (-6.19, 1.9) |
| Placebo | 0.52 (-2.3, 3.31) | -1.25 (-4.15, 1.52) | -4.56 (-7.03, -2.02) | -4.38 (-6.77, -1.97) | -2.48 (-3.56, -1.38) | -3.8 (-4.8, -2.88) | -0.81 (-2.38, 0.72) | Placebo | -1.84 (-4.92, 1.14) | -2.18 (-5.53, 1.16) | -3.85 (-6.96, -0.63) | -4.62 (-7.79, -1.68) | -4.64 (-8.33, -0.91) | -2.93 (-6.7, 0.74) |
| Remi100mg bid | 2.35 (-1.62, 6.51) | 0.58 (-3.49, 4.65) | -2.74 (-6.43, 1.28) | -2.55 (-6.26, 1.4) | -0.64 (-3.83, 2.69) | -1.98 (-5.06, 1.24) | 1.02 (-2.27, 4.52) | 1.84 (-1.14, 4.92) | Remi100mg bid | -0.37 (-3.44, 2.81) | -2 (-4.85, 1.02) | -2.79 (-5.63, 0.02) | -2.75 (-6.4, 0.65) | -1.15 (-4.62, 2.43) |
| Remi100mg qd | 2.69 (-1.58, 7.05) | 0.9 (-3.47, 5.21) | -2.41 (-6.43, 1.92) | -2.17 (-6.34, 1.94) | -0.29 (-3.79, 3.25) | -1.61 (-5.08, 1.8) | 1.4 (-2.33, 5.08) | 2.18 (-1.16, 5.53) | 0.37 (-2.81, 3.44) | Remi100mg qd | -1.64 (-4.91, 1.67) | -2.46 (-5.62, 0.69) | -2.45 (-6.37, 1.38) | -0.81 (-4.48, 3.2) |
| Remi10mg bid | 4.39 (0.19, 8.48) | 2.56 (-1.59, 6.74) | -0.78 (-4.61, 3.36) | -0.51 (-4.43, 3.42) | 1.37 (-1.96, 4.73) | 0.04 (-3.23, 3.26) | 3.05 (-0.46, 6.52) | 3.85 (0.63, 6.96) | 2 (-1.02, 4.85) | 1.64 (-1.67, 4.91) | Remi10mg bid | -0.81 (-3.83, 2.17) | -0.78 (-4.43, 2.77) | 0.85 (-2.81, 4.53) |
| Remi10mg qd | 5.13 (1.14, 9.36) | 3.36 (-0.74, 7.59) | 0.08 (-3.73, 4.09) | 0.28 (-3.56, 4.28) | 2.15 (-0.97, 5.5) | 0.82 (-2.29, 4.06) | 3.81 (0.46, 7.34) | 4.62 (1.68, 7.79) | 2.79 (-0.02, 5.63) | 2.46 (-0.69, 5.62) | 0.81 (-2.17, 3.83) | Remi10mg qd | 0.04 (-3.72, 3.72) | 1.63 (-1.87, 5.38) |
| Remi25mg bid | 5.17 (0.62, 9.75) | 3.35 (-1.22, 8.01) | 0.04 (-4.31, 4.63) | 0.23 (-4.12, 4.73) | 2.15 (-1.77, 6.02) | 0.82 (-3.05, 4.62) | 3.82 (-0.25, 7.87) | 4.64 (0.91, 8.33) | 2.75 (-0.65, 6.4) | 2.45 (-1.38, 6.37) | 0.78 (-2.77, 4.43) | -0.04 (-3.72, 3.72) | Remi25mg bid | 1.63 (-2.48, 6.04) |
| Remi35mg qd | 3.49 (-1.26, 8.12) | 1.69 (-3.09, 6.39) | -1.58 (-5.93, 2.9) | -1.39 (-5.83, 3.06) | 0.46 (-3.36, 4.34) | -0.86 (-4.69, 2.94) | 2.13 (-1.9, 6.19) | 2.93 (-0.74, 6.7) | 1.15 (-2.43, 4.62) | 0.81 (-3.2, 4.48) | -0.85 (-4.53, 2.81) | -1.63 (-5.38, 1.87) | -1.63 (-6.04, 2.48) | Remi35mg qd |
| SUCRA | 0.0773 | 0.3006 | 0.7962 | 0.7683 | 0.4563 | 0.6958 | 0.2161 | 0.0967 | 0.3523 | 0.4144 | 0.6848 | 0.8115 | 0.7866 | 0.5431 |

For change from baseline in DLQI, Network meta-analysis results are expressed as MD (95% CI). The value of MD less than 0 indicate that the treatment specified in the row is more effective than that specified in the column. The bottom row contains the Surface Under the Cumulative Ranking (SUCRA) value for the column-defining treatment. Both in efficacy and safety, a higher score on the lower surface under the cumulative ranking curve (SUCRA) indicate better efficacy and higher safety.

**eFigure 15.** Cluster ranking plot of change in weekly urticaria activity score (UAS7) and proportion of patients achieved complete response (UAS7=0). Both in efficacy and safety, a higher score on the lower surface under the cumulative ranking curve (SUCRA) indicate better efficacy and higher safety.


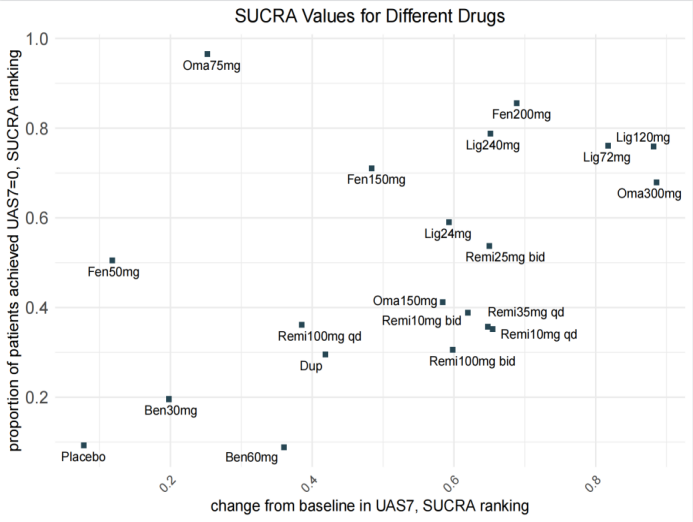


**eFigure 16.** Cluster ranking plot of itch severity and hives severity.


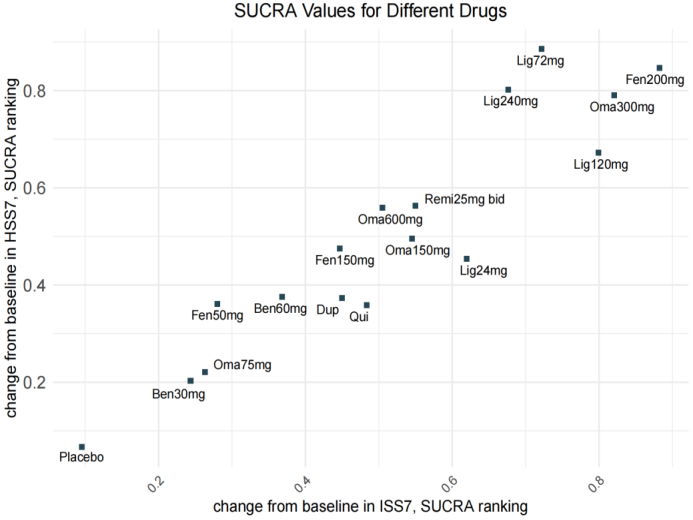


**eFigure 17.** Cluster ranking plot of adverse events and serious adverse events.


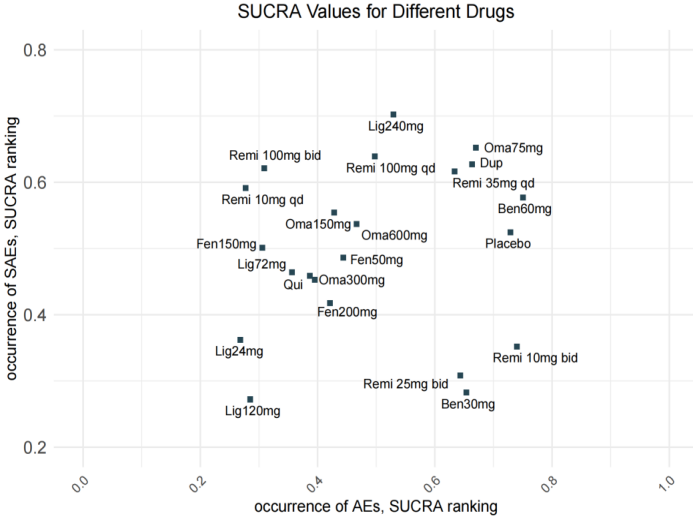


**eTable 14.** Analysis of heterogeneity. ("i2.pair" measures heterogeneity between each pair of studies, while "i2.cons" measures heterogeneity in the overall effect across all studies. "incons.p" indicates the significance level of heterogeneity, with p < 0.05 suggesting non-random heterogeneity possibly due to genuine differences).

A. Change in weekly urticaria activity score (UAS7).

| **Per-comparison I-squared:** |  |  |  |  |
| --- | --- | --- | --- | --- |
| **Treatment1** | **Treatment2** | **i2.pair** | **i2.cons** | **incons.p** |
| AZD | Placebo | NA | NA | NA |
| Ben30mg | Ben60mg | NA | NA | NA |
| Ben30mg | Placebo | NA | NA | NA |
| Ben60mg | Placebo | NA | NA | NA |
| Dup | Placebo | 0 | 0 | NA |
| Fen150mg | Fen200mg | NA | NA | NA |
| Fen150mg | Fen50mg | NA | NA | NA |
| Fen150mg | Placebo | NA | NA | NA |
| Fen200mg | Fen50mg | NA | NA | NA |
| Fen200mg | Placebo | 0 | 0 | NA |
| Fen50mg | Placebo | NA | NA | NA |
| Lig120mg | Lig24mg | NA | 0 | 0.4212825 |
| Lig120mg | Lig72mg | 4.743991 | **66.677149** | **0.02545775** |
| Lig120mg | Oma300mg | 0 | 0 | 0.93096269 |
| Lig120mg | Placebo | 29.420629 | 0 | 0.72099183 |
| Lig240mg | Lig24mg | NA | NA | NA |
| Lig240mg | Lig72mg | NA | NA | NA |
| Lig240mg | Oma300mg | NA | NA | NA |
| Lig240mg | Placebo | NA | NA | NA |
| Lig24mg | Lig72mg | NA | 0 | 0.70115828 |
| Lig24mg | Oma300mg | NA | 0 | 0.95822557 |
| Lig24mg | Placebo | NA | 0 | 0.97701052 |
| Lig72mg | Oma300mg | 0 | 0 | NA |
| Lig72mg | Placebo | 0 | 0 | NA |
| Oma150mg | Oma300mg | 6.67592 | 8.59073 | NA |
| Oma150mg | Oma75mg | 0 | 0 | 0.56919367 |
| Oma150mg | Placebo | 0 | 0 | NA |
| Oma300mg | Oma600mg | NA | NA | NA |
| Oma300mg | Oma75mg | 0 | 0 | NA |
| Oma300mg | Placebo | 0 | 0 | NA |
| Oma300mg | Tez210mg | NA | NA | NA |
| Oma300mg | Tez420mg | NA | NA | NA |
| Oma600mg | Oma75mg | NA | NA | NA |
| Oma600mg | Placebo | NA | NA | NA |
| Oma75mg | Placebo | 0 | 0 | NA |
| Placebo | Qui | NA | NA | NA |
| Placebo | Remi_100mg_bid | NA | NA | NA |
| Placebo | Remi_100mg_qd | NA | NA | NA |
| Placebo | Remi_10mg_bid | NA | NA | NA |
| Placebo | Remi_10mg_qd | NA | NA | NA |
| Placebo | Remi_25mg_bid | 0 | 4.385713 | NA |
| Placebo | Remi_35mg_qd | NA | NA | NA |
| Placebo | Ril | NA | NA | NA |
| Placebo | Tez210mg | NA | NA | NA |
| Placebo | Tez420mg | NA | NA | NA |
| Remi_100mg_bid | Remi_100mg_qd | NA | NA | NA |
| Remi_100mg_bid | Remi_10mg_bid | NA | NA | NA |
| Remi_100mg_bid | Remi_10mg_qd | NA | NA | NA |
| Remi_100mg_bid | Remi_25mg_bid | NA | NA | NA |
| Remi_100mg_bid | Remi_35mg_qd | NA | NA | NA |
| Remi_100mg_qd | Remi_10mg_bid | NA | NA | NA |
| Remi_100mg_qd | Remi_10mg_qd | NA | NA | NA |
| Remi_100mg_qd | Remi_25mg_bid | NA | NA | NA |
| Remi_100mg_qd | Remi_35mg_qd | NA | NA | NA |
| Remi_10mg_bid | Remi_10mg_qd | NA | NA | NA |
| Remi_10mg_bid | Remi_25mg_bid | NA | NA | NA |
| Remi_10mg_bid | Remi_35mg_qd | NA | NA | NA |
| Remi_10mg_qd | Remi_25mg_bid | NA | NA | NA |
| Remi_10mg_qd | Remi_35mg_qd | NA | NA | NA |
| Remi_25mg_bid | Remi_35mg_qd | NA | NA | NA |
| Tez210mg | Tez420mg | NA | NA | NA |
| **Global I-squared:** |  |  |  |  |
| **i2.pair** | **i2.cons** |  |  |  |
| 0 | 0 |  |  |  |

1. Proportion of patients achieved well-controlled disease (UAS7≤6).

| **Per-comparison I-squared:** |  |  |  |  |
| --- | --- | --- | --- | --- |
| **Treatment1** | **Treatment2** | **i2.pair** | **i2.cons** | **incons.p** |
| Ben30mg | Ben60mg | NA | NA | NA |
| Ben30mg | Placebo | NA | NA | NA |
| Ben60mg | Placebo | NA | NA | NA |
| Dup | Placebo | NA | NA | NA |
| Fen150mg | Fen200mg | NA | NA | NA |
| Fen150mg | Fen50mg | NA | NA | NA |
| Fen150mg | Placebo | NA | NA | NA |
| Fen200mg | Fen50mg | NA | NA | NA |
| Fen200mg | Placebo | 0 | 0 | NA |
| Fen50mg | Placebo | NA | NA | NA |
| Oma150mg | Oma300mg | 29.57298 | 35.38055 | NA |
| Oma150mg | Oma75mg | 0 | 0 | NA |
| Oma150mg | Placebo | 0 | 0 | NA |
| Oma300mg | Oma75mg | 0 | 0 | NA |
| Oma300mg | Placebo | 0 | 0 | NA |
| Oma75mg | Placebo | 0 | 0 | NA |
| Placebo | Remi_100mg_bid | NA | NA | NA |
| Placebo | Remi_100mg_qd | NA | NA | NA |
| Placebo | Remi_10mg_bid | NA | NA | NA |
| Placebo | Remi_10mg_qd | NA | NA | NA |
| Placebo | Remi_25mg_bid | 0 | 0 | NA |
| Placebo | Remi_35mg_qd | NA | NA | NA |
| Remi_100mg_bid | Remi_100mg_qd | NA | NA | NA |
| Remi_100mg_bid | Remi_10mg_bid | NA | NA | NA |
| Remi_100mg_bid | Remi_10mg_qd | NA | NA | NA |
| Remi_100mg_bid | Remi_25mg_bid | NA | NA | NA |
| Remi_100mg_bid | Remi_35mg_qd | NA | NA | NA |
| Remi_100mg_qd | Remi_10mg_bid | NA | NA | NA |
| Remi_100mg_qd | Remi_10mg_qd | NA | NA | NA |
| Remi_100mg_qd | Remi_25mg_bid | NA | NA | NA |
| Remi_100mg_qd | Remi_35mg_qd | NA | NA | NA |
| Remi_10mg_bid | Remi_10mg_qd | NA | NA | NA |
| Remi_10mg_bid | Remi_25mg_bid | NA | NA | NA |
| Remi_10mg_bid | Remi_35mg_qd | NA | NA | NA |
| Remi_10mg_qd | Remi_25mg_bid | NA | NA | NA |
| Remi_10mg_qd | Remi_35mg_qd | NA | NA | NA |
| Remi_25mg_bid | Remi_35mg_qd | NA | NA | NA |
| **Global I-squared:** |  |  |  |  |
| **i2.pair** | **i2.cons** |  |  |  |
| 0 | 0 |  |  |  |

1. Proportion of patients achieved complete response to treatment (UAS7=0).

| **Per-comparison I-squared:** |  |  |  |  |
| --- | --- | --- | --- | --- |
| **Treatment1** | **Treatment2** | **i2.pair** | **i2.cons** | **incons.p** |
| Ben30mg | Ben60mg | NA | NA | NA |
| Ben30mg | Placebo | NA | NA | NA |
| Ben60mg | Placebo | NA | NA | NA |
| Dup | Placebo | NA | NA | NA |
| Fen150mg | Fen200mg | NA | NA | NA |
| Fen150mg | Fen50mg | NA | NA | NA |
| Fen150mg | Placebo | NA | NA | NA |
| Fen200mg | Fen50mg | NA | NA | NA |
| Fen200mg | Placebo | NA | NA | NA |
| Fen50mg | Placebo | NA | NA | NA |
| Lig120mg | Lig24mg | NA | 10.52476 | 0.31690678 |
| Lig120mg | Lig72mg | 37.91391 | 53.59251 | NA |
| Lig120mg | Oma300mg | 0 | 0 | NA |
| Lig120mg | Placebo | **75.99644** | **81.49518** | **0.01933547** |
| Lig240mg | Lig24mg | NA | NA | NA |
| Lig240mg | Lig72mg | NA | NA | NA |
| Lig240mg | Oma300mg | NA | NA | NA |
| Lig240mg | Placebo | NA | NA | NA |
| Lig24mg | Lig72mg | NA | 0 | 0.75572273 |
| Lig24mg | Oma300mg | NA | 0 | 0.3888719 |
| Lig24mg | Placebo | NA | 0 | 0.76898447 |
| Lig72mg | Oma300mg | 62.05286 | 60.40259 | NA |
| Lig72mg | Placebo | 52.90324 | 75.37415 | NA |
| Oma150mg | Oma300mg | 0 | 0 | NA |
| Oma150mg | Oma75mg | NA | NA | NA |
| Oma150mg | Placebo | 0 | 0 | NA |
| Oma300mg | Oma75mg | NA | NA | NA |
| Oma300mg | Placebo | 26.66824 | 27.0064 | NA |
| Oma75mg | Placebo | NA | NA | NA |
| Placebo | Remi_100mg_bid | NA | NA | NA |
| Placebo | Remi_100mg_qd | NA | NA | NA |
| Placebo | Remi_10mg_bid | NA | NA | NA |
| Placebo | Remi_10mg_qd | NA | NA | NA |
| Placebo | Remi_25mg_bid | 0 | 0 | NA |
| Placebo | Remi_35mg_qd | NA | NA | NA |
| Remi_100mg_bid | Remi_100mg_qd | NA | NA | NA |
| Remi_100mg_bid | Remi_10mg_bid | NA | NA | NA |
| Remi_100mg_bid | Remi_10mg_qd | NA | NA | NA |
| Remi_100mg_bid | Remi_25mg_bid | NA | NA | NA |
| Remi_100mg_bid | Remi_35mg_qd | NA | NA | NA |
| Remi_100mg_qd | Remi_10mg_bid | NA | NA | NA |
| Remi_100mg_qd | Remi_10mg_qd | NA | NA | NA |
| Remi_100mg_qd | Remi_25mg_bid | NA | NA | NA |
| Remi_100mg_qd | Remi_35mg_qd | NA | NA | NA |
| Remi_10mg_bid | Remi_10mg_qd | NA | NA | NA |
| Remi_10mg_bid | Remi_25mg_bid | NA | NA | NA |
| Remi_10mg_bid | Remi_35mg_qd | NA | NA | NA |
| Remi_10mg_qd | Remi_25mg_bid | NA | NA | NA |
| Remi_10mg_qd | Remi_35mg_qd | NA | NA | NA |
| Remi_25mg_bid | Remi_35mg_qd | NA | NA | NA |
| **Global I-squared:** |  |  |  |  |
| **i2.pair** | **i2.cons** |  |  |  |
| 29.78372 | 0 |  |  |  |

1. Adverse Events (AEs)

| **Per-comparison I-squared:** |  |  |  |  |
| --- | --- | --- | --- | --- |
| **Treatment 1** | **Treatment 2** | **i2.pair** | **i2.cons** | **incons.p** |
| AZD | Placebo | NA | NA | NA |
| Ben30mg | Ben60mg | NA | NA | NA |
| Ben30mg | Placebo | NA | NA | NA |
| Ben60mg | Placebo | NA | NA | NA |
| Dup | Placebo | NA | NA | NA |
| Fen150mg | Fen200mg | NA | NA | NA |
| Fen150mg | Fen50mg | NA | NA | NA |
| Fen150mg | Placebo | NA | NA | NA |
| Fen200mg | Fen50mg | NA | NA | NA |
| Fen200mg | Placebo | 0 | 0 | NA |
| Fen50mg | Placebo | NA | NA | NA |
| Lig120mg | Lig24mg | NA | 1.85622 | 0.34495518 |
| Lig120mg | Lig72mg | 0 | 0 | NA |
| Lig120mg | Oma300mg | 70.420615 | 82.014797 | NA |
| Lig120mg | Placebo | 0 | **59.66527** | **0.02786529** |
| Lig240mg | Lig24mg | NA | NA | NA |
| Lig240mg | Lig72mg | NA | NA | NA |
| Lig240mg | Oma300mg | NA | NA | NA |
| Lig240mg | Placebo | NA | NA | NA |
| Lig24mg | Lig72mg | NA | 0 | 0.73045531 |
| Lig24mg | Oma300mg | NA | 0 | 0.45675556 |
| Lig24mg | Placebo | NA | 0 | 0.71666589 |
| Lig72mg | Oma300mg | 24.903998 | 44.340668 | NA |
| Lig72mg | Placebo | 0 | 39.326655 | NA |
| Oma150mg | Oma300mg | 0 | 0 | NA |
| Oma150mg | Oma75mg | 0 | 0 | 0.6843887 |
| Oma150mg | Placebo | 0 | 0 | 0.58164053 |
| Oma300mg | Oma600mg | NA | NA | NA |
| Oma300mg | Oma75mg | 0 | 0 | NA |
| Oma300mg | Placebo | 8.298555 | 8.531712 | NA |
| Oma600mg | Oma75mg | NA | NA | NA |
| Oma600mg | Placebo | NA | NA | NA |
| Oma75mg | Placebo | 0 | 0 | NA |
| Placebo | Qui | NA | NA | NA |
| Placebo | Remi_100mg_bid | NA | NA | NA |
| Placebo | Remi_100mg_qd | NA | NA | NA |
| Placebo | Remi_10mg_bid | NA | NA | NA |
| Placebo | Remi_10mg_qd | NA | NA | NA |
| Placebo | Remi25mg | 17.527734 | 27.457744 | NA |
| Placebo | Remi35mg | NA | NA | NA |
| Remi_100mg_bid | Remi_100mg_qd | NA | NA | NA |
| Remi_100mg_bid | Remi_10mg_bid | NA | NA | NA |
| Remi_100mg_bid | Remi_10mg_qd | NA | NA | NA |
| Remi_100mg_bid | Remi25mg | NA | NA | NA |
| Remi_100mg_bid | Remi35mg | NA | NA | NA |
| Remi_100mg_qd | Remi_10mg_bid | NA | NA | NA |
| Remi_100mg_qd | Remi_10mg_qd | NA | NA | NA |
| Remi_100mg_qd | Remi25mg | NA | NA | NA |
| Remi_100mg_qd | Remi35mg | NA | NA | NA |
| Remi_10mg_bid | Remi_10mg_qd | NA | NA | NA |
| Remi_10mg_bid | Remi25mg | NA | NA | NA |
| Remi_10mg_bid | Remi35mg | NA | NA | NA |
| Remi_10mg_qd | Remi25mg | NA | NA | NA |
| Remi_10mg_qd | Remi35mg | NA | NA | NA |
| Remi25mg | Remi35mg | NA | NA | NA |
| **Global I-squared:** |  |  |  |  |
| **i2.pair** | **i2.cons** |  |  |  |
| 0 | 0 |  |  |  |

1. Serious Adverse Events (SAEs)

| **Per-comparison I-squared:** |  |  |  |  |
| --- | --- | --- | --- | --- |
| **Treatment 1** | **Treatment 2** | **i2.pair** | **i2.cons** | **incons.p** |
| Benralizumab_30mg_qd | Benralizumab_60mg_qd | NA | NA | NA |
| Benralizumab_30mg_qd | Placebo | NA | NA | NA |
| Benralizumab_60mg_qd | Placebo | NA | NA | NA |
| Dupilumab | Placebo | NA | NA | NA |
| Fenebrutinib_150mg_qd | Fenebrutinib_200mg_bid | NA | NA | NA |
| Fenebrutinib_150mg_qd | Fenebrutinib_50mg_qd | NA | NA | NA |
| Fenebrutinib_150mg_qd | Placebo | NA | NA | NA |
| Fenebrutinib_200mg_bid | Fenebrutinib_50mg_qd | NA | NA | NA |
| Fenebrutinib_200mg_bid | Placebo | 0 | 0 | NA |
| Fenebrutinib_50mg_qd | Placebo | NA | NA | NA |
| Ligelizumab_120mg_q4w | Ligelizumab_24mg_q4w | NA | 0 | 0.8335556 |
| Ligelizumab_120mg_q4w | Ligelizumab_72mg_q4w | 0 | 0 | 0.3530878 |
| Ligelizumab_120mg_q4w | Omalizumab_300mg_q4w | 0 | 0 | 0.748527 |
| Ligelizumab_120mg_q4w | Placebo | 0 | 0 | 0.4349489 |
| Ligelizumab_240mg_q4w | Ligelizumab_24mg_q4w | NA | NA | NA |
| Ligelizumab_240mg_q4w | Ligelizumab_72mg_q4w | NA | NA | NA |
| Ligelizumab_240mg_q4w | Omalizumab_300mg_q4w | NA | NA | NA |
| Ligelizumab_240mg_q4w | Placebo | NA | NA | NA |
| Ligelizumab_24mg_q4w | Ligelizumab_72mg_q4w | NA | 0 | 0.7920557 |
| Ligelizumab_24mg_q4w | Omalizumab_300mg_q4w | NA | 0 | 0.5779255 |
| Ligelizumab_24mg_q4w | Placebo | NA | 0 | 0.4635898 |
| Ligelizumab_72mg_q4w | Omalizumab_300mg_q4w | 0 | 0 | NA |
| Ligelizumab_72mg_q4w | Placebo | 0 | 0 | NA |
| Omalizumab_150mg_q4w | Omalizumab_300mg_q4w | 0 | 0 | NA |
| Omalizumab_150mg_q4w | Omalizumab_75mg_q4w | 0 | 0 | 0.9193058 |
| Omalizumab_150mg_q4w | Placebo | 0 | 0 | NA |
| Omalizumab_300mg_q4w | Omalizumab_600mg_q4w | NA | NA | NA |
| Omalizumab_300mg_q4w | Omalizumab_75mg_q4w | 0 | 0 | NA |
| Omalizumab_300mg_q4w | Placebo | 0 | 0 | NA |
| Omalizumab_600mg_q4w | Omalizumab_75mg_q4w | NA | NA | NA |
| Omalizumab_600mg_q4w | Placebo | NA | NA | NA |
| Omalizumab_75mg_q4w | Placebo | 0 | 0 | NA |
| Placebo | Quilizumab_450mg_q4w | NA | NA | NA |
| Placebo | Remibrutinib_100mg_bid | NA | NA | NA |
| Placebo | Remibrutinib_100mg_qd | NA | NA | NA |
| Placebo | Remibrutinib_10mg_bid | NA | NA | NA |
| Placebo | Remibrutinib_10mg_qd | NA | NA | NA |
| Placebo | Remibrutinib_25mg_bid | 27.18263 | 28.76127 | NA |
| Placebo | Remibrutinib_35mg_qd | NA | NA | NA |
| Remibrutinib_100mg_bid | Remibrutinib_100mg_qd | NA | NA | NA |
| Remibrutinib_100mg_bid | Remibrutinib_10mg_bid | NA | NA | NA |
| Remibrutinib_100mg_bid | Remibrutinib_10mg_qd | NA | NA | NA |
| Remibrutinib_100mg_bid | Remibrutinib_25mg_bid | NA | NA | NA |
| Remibrutinib_100mg_bid | Remibrutinib_35mg_qd | NA | NA | NA |
| Remibrutinib_100mg_qd | Remibrutinib_10mg_bid | NA | NA | NA |
| Remibrutinib_100mg_qd | Remibrutinib_10mg_qd | NA | NA | NA |
| Remibrutinib_100mg_qd | Remibrutinib_25mg_bid | NA | NA | NA |
| Remibrutinib_100mg_qd | Remibrutinib_35mg_qd | NA | NA | NA |
| Remibrutinib_10mg_bid | Remibrutinib_10mg_qd | NA | NA | NA |
| Remibrutinib_10mg_bid | Remibrutinib_25mg_bid | NA | NA | NA |
| Remibrutinib_10mg_bid | Remibrutinib_35mg_qd | NA | NA | NA |
| Remibrutinib_10mg_qd | Remibrutinib_25mg_bid | NA | NA | NA |
| Remibrutinib_10mg_qd | Remibrutinib_35mg_qd | NA | NA | NA |
| Remibrutinib_25mg_bid | Remibrutinib_35mg_qd | NA | NA | NA |
| **Global I-squared:** |  |  |  |  |
| **i2.pair** | **i2.cons** |  |  |  |
| 0 | 0 |  |  |  |

**eFigure 18.** Subgroup and sensitivity analysis.

1. Change in weekly urticaria activity score (UAS7)

A1. Forest plot for change in weekly urticaria activity score (UAS7) in the subgroup of adults.


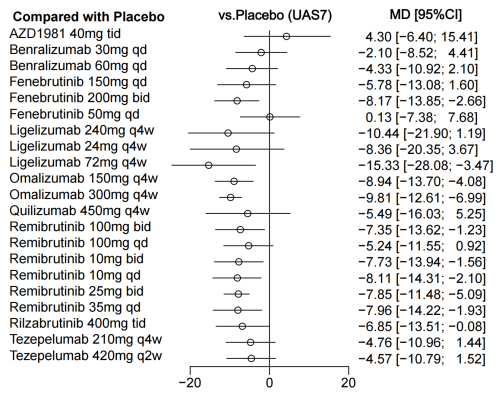


A2. Forest plot for change in weekly urticaria activity score (UAS7) in the subgroup of treatment duration≤16 weeks.


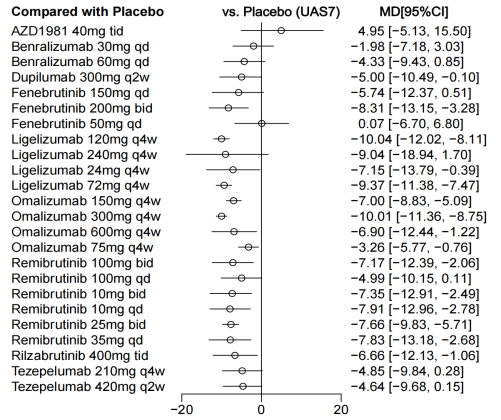


A3. Sensitivity analysis of network meta-analysis: change in the weekly urticaria activity score. (The y-axis represents the excluded drug, and the x-axis represents the overall mean response rate. The length of the bars indicates the overall mean response rate after excluding the respective drug. Longer bars signify a higher overall mean response rate after excluding the drug, suggesting that the remaining drugs perform better when the excluded drug is not considered.)


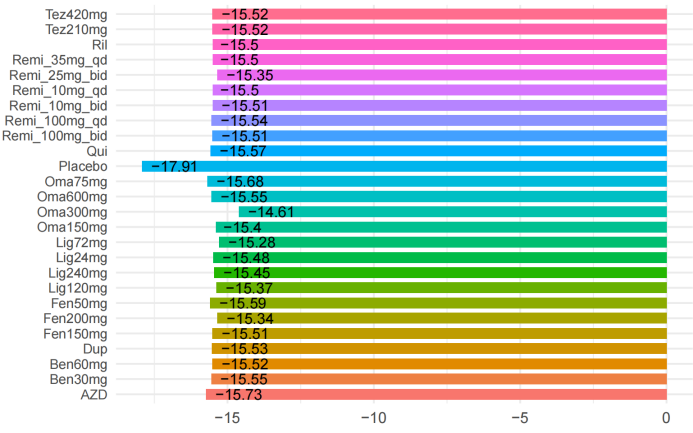


1. Proportion of patients achieved well-controlled disease (UAS7≤6)

B1. Forest plot for proportion of patients achieved well-controlled disease (UAS7≤6) in the subgroup of adults.


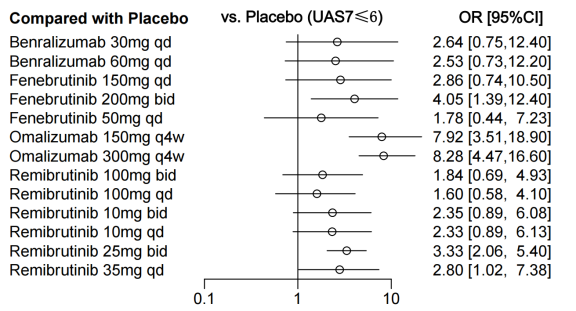


B2. Sensitivity analysis of network meta-analysis: proportion of patients achieved well-controlled disease. (The y-axis represents the excluded drug, and the x-axis represents the overall mean response rate. The length of the bars indicates the overall mean response rate after excluding the respective drug. Longer bars signify a higher overall mean response rate after excluding the drug, suggesting that the remaining drugs perform better when the excluded drug is not considered.)


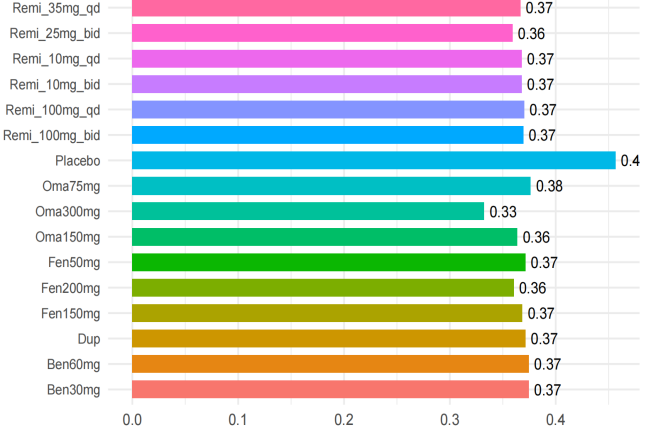


1. Proportion of patients achieved complete response to treatment (UAS7=0)

C1. Forest plot for proportion of patients achieved complete response to treatment (UAS7=0) in the subgroup of adults.


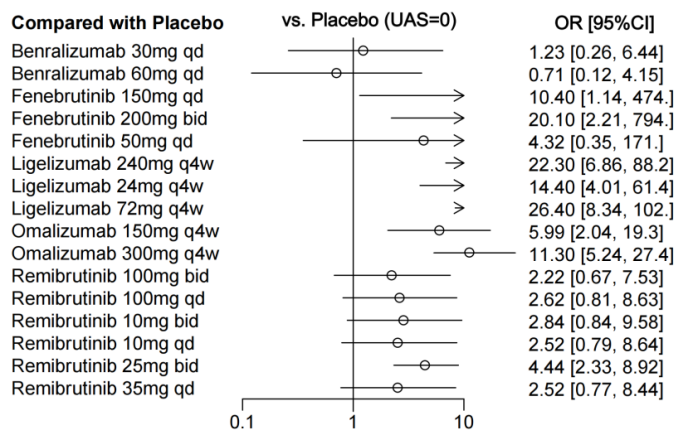


C2. Sensitivity analysis of network meta-analysis: proportion of patients achieved complete response to treatment. (The y-axis represents the excluded drug, and the x-axis represents the overall mean response rate. The length of the bars indicates the overall mean response rate after excluding the respective drug. Longer bars signify a higher overall mean response rate after excluding the drug, suggesting that the remaining drugs perform better when the excluded drug is not considered.)


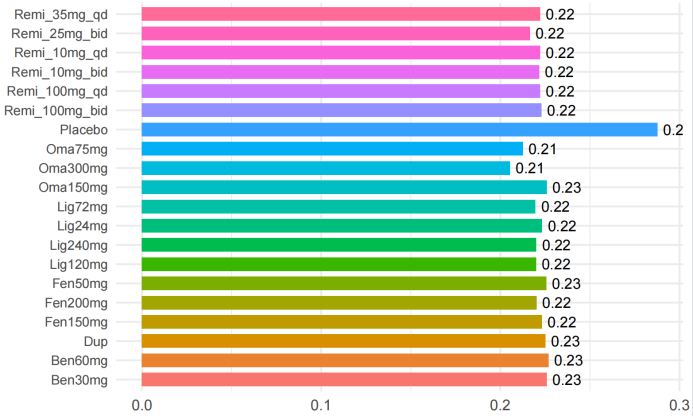


1. Adverse events (AEs)

D1. Forest plot for adverse events (AEs) in the subgroup of adults.


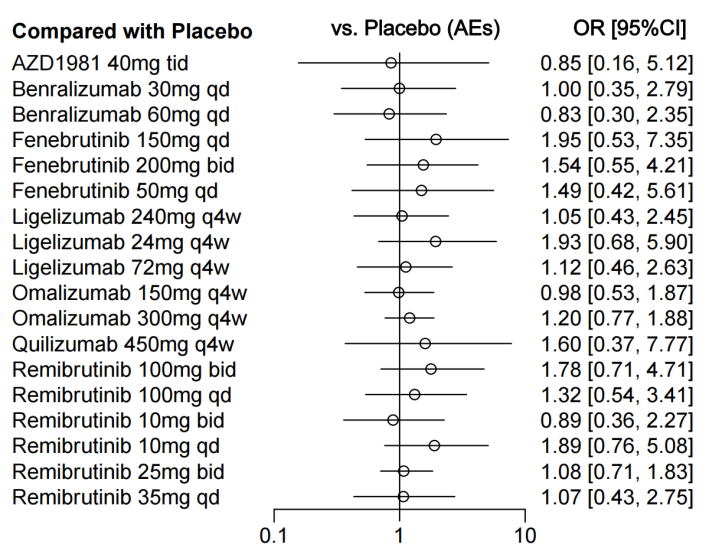


D2. Forest plot for adverse events (AEs) in the subgroup of treatment duration≤16 weeks.


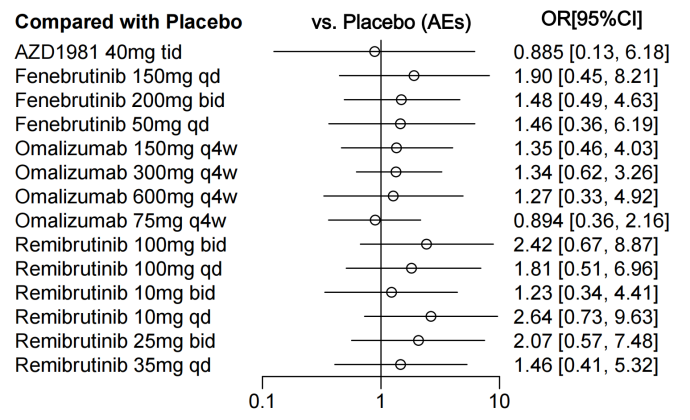


D3. Forest plot for adverse events (AEs) in the subgroup of treatment duration＞16 weeks.


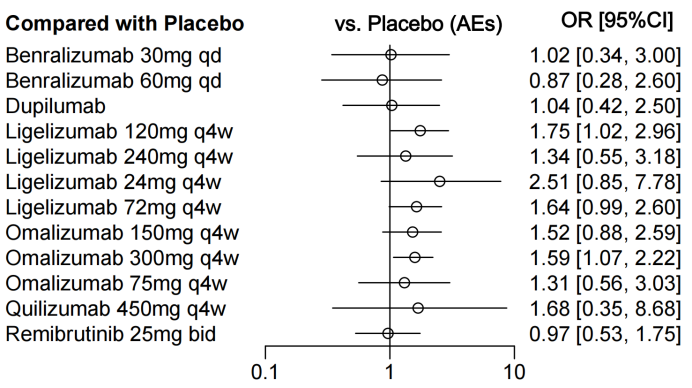


D4. Sensitivity analysis of network meta-analysis: adverse events. (The y-axis represents the excluded drug, and the x-axis represents the overall mean response rate. The length of the bars indicates the overall mean response rate after excluding the respective drug. Longer bars signify a higher overall mean response rate after excluding the drug, suggesting that the remaining drugs perform better when the excluded drug is not considered.)


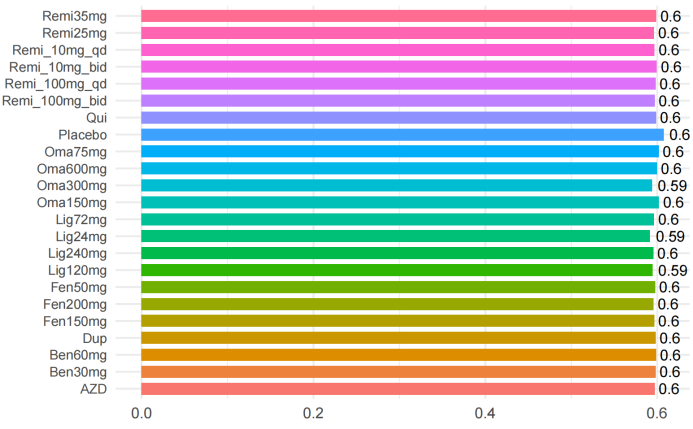


**eFigure 19.** Net splitting for the network analysis. (A statistically significant inconsistency was considered to exist for a comparison group if p-value was significantly less than 0.05 or CI did not contain 0)

1. Change in weekly urticaria activity score (UAS7).


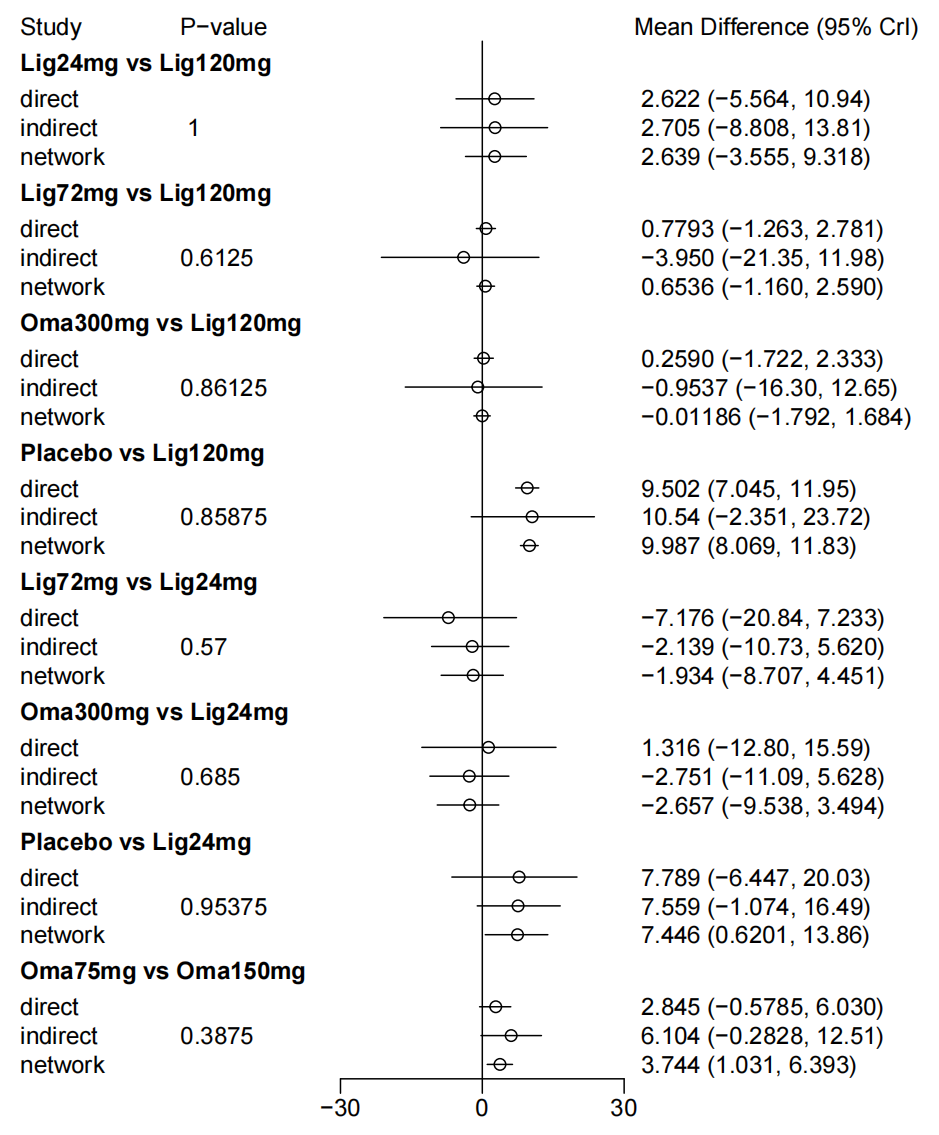


1. Proportion of patients achieved complete response to treatment (UAS7=0).


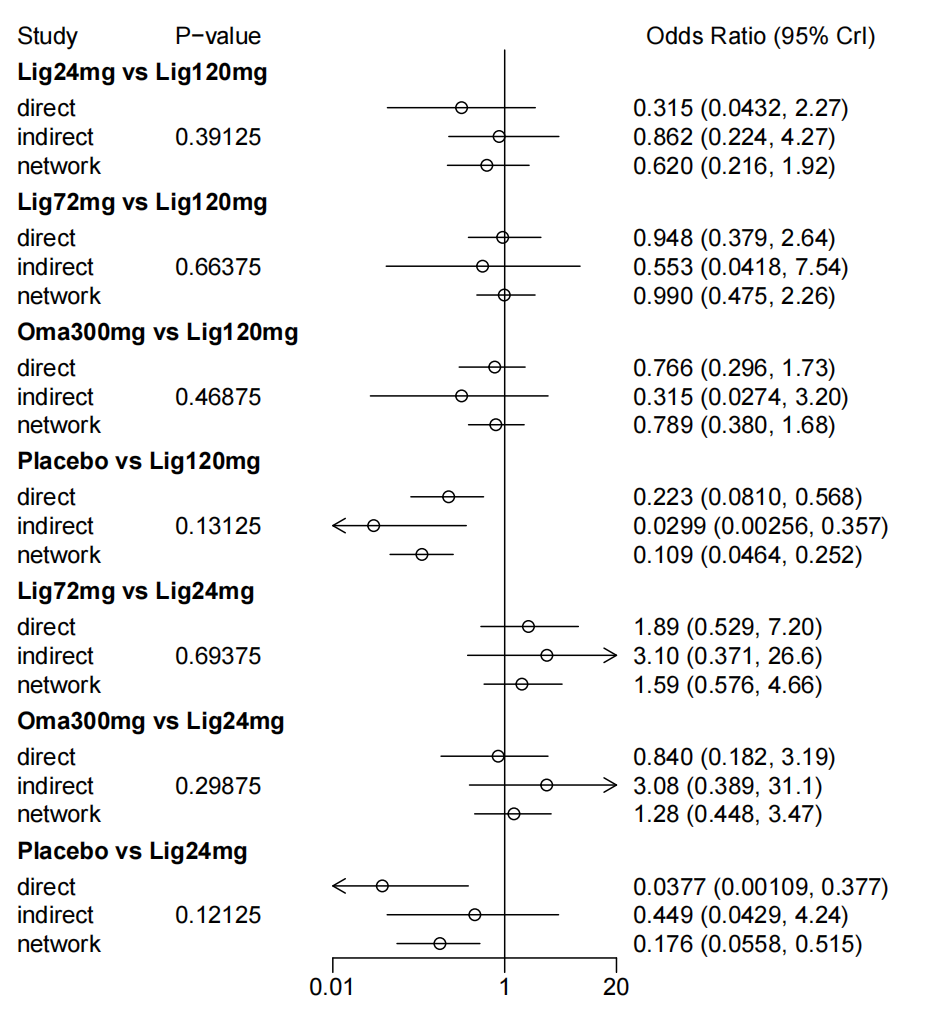


1. Weekly itch severity score (ISS7).


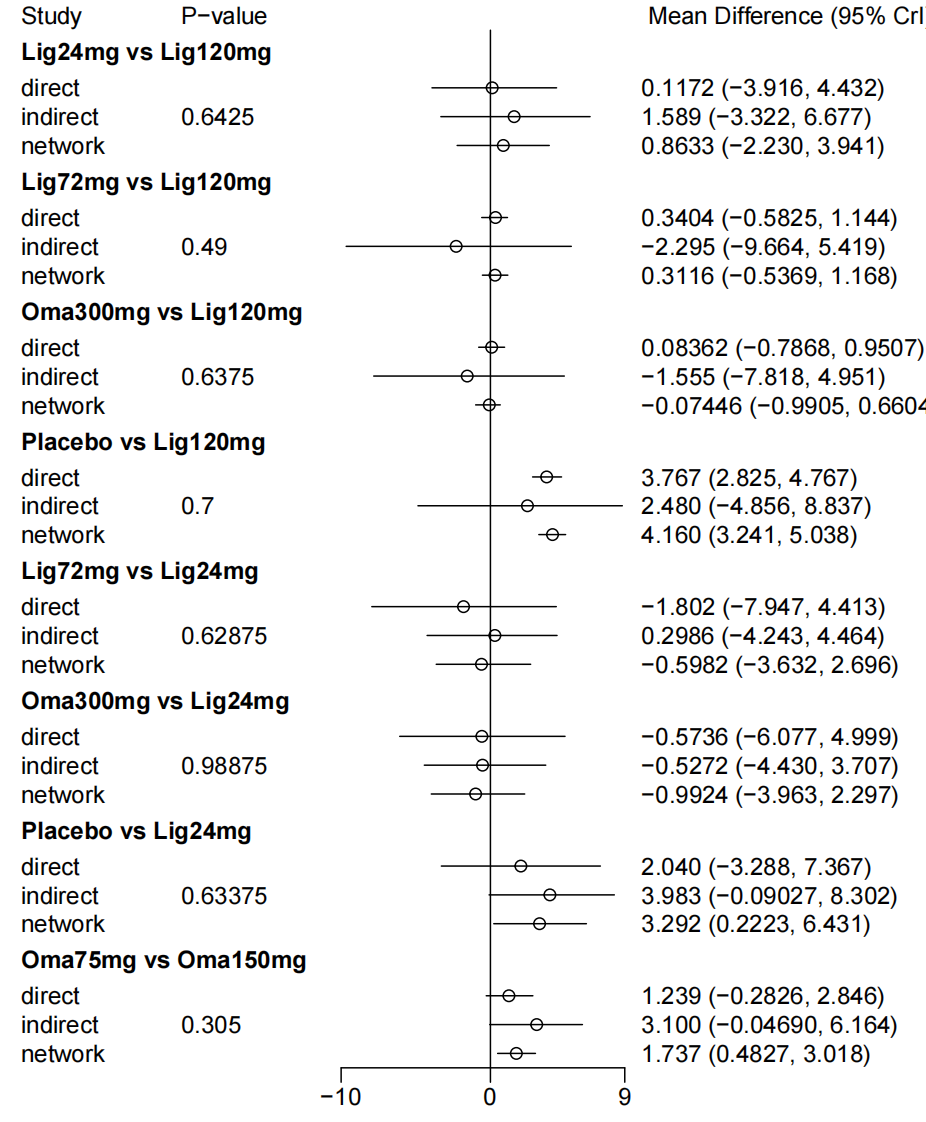


1. Weekly hive severity score (HSS7)


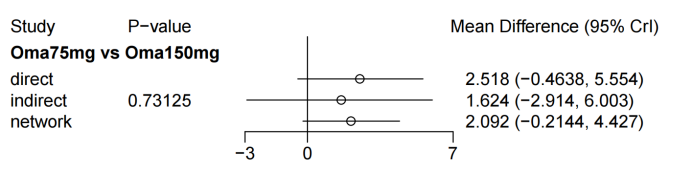


1. Adverse Events (AEs)


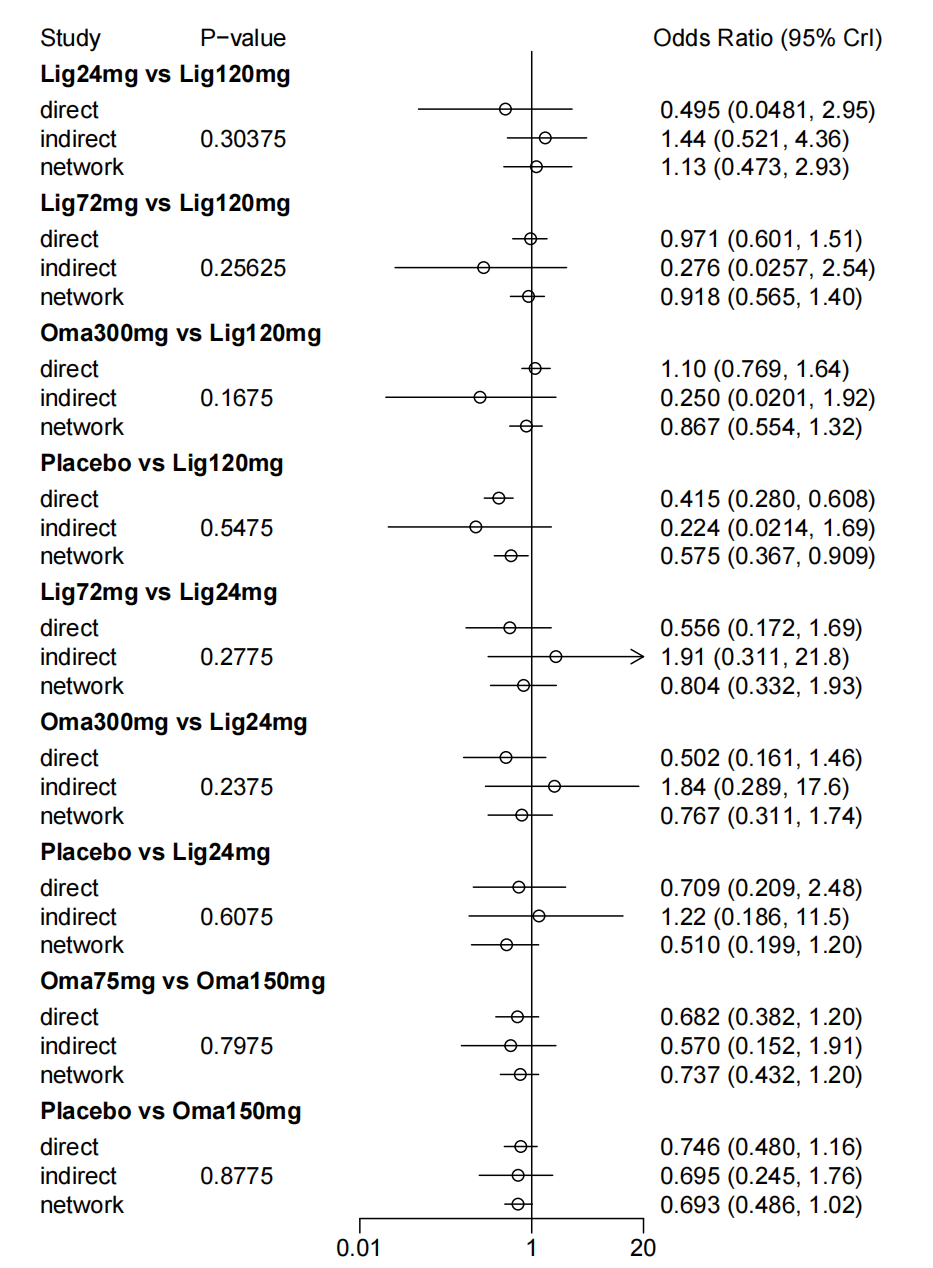


1. Serious Adverse Events (SAEs)


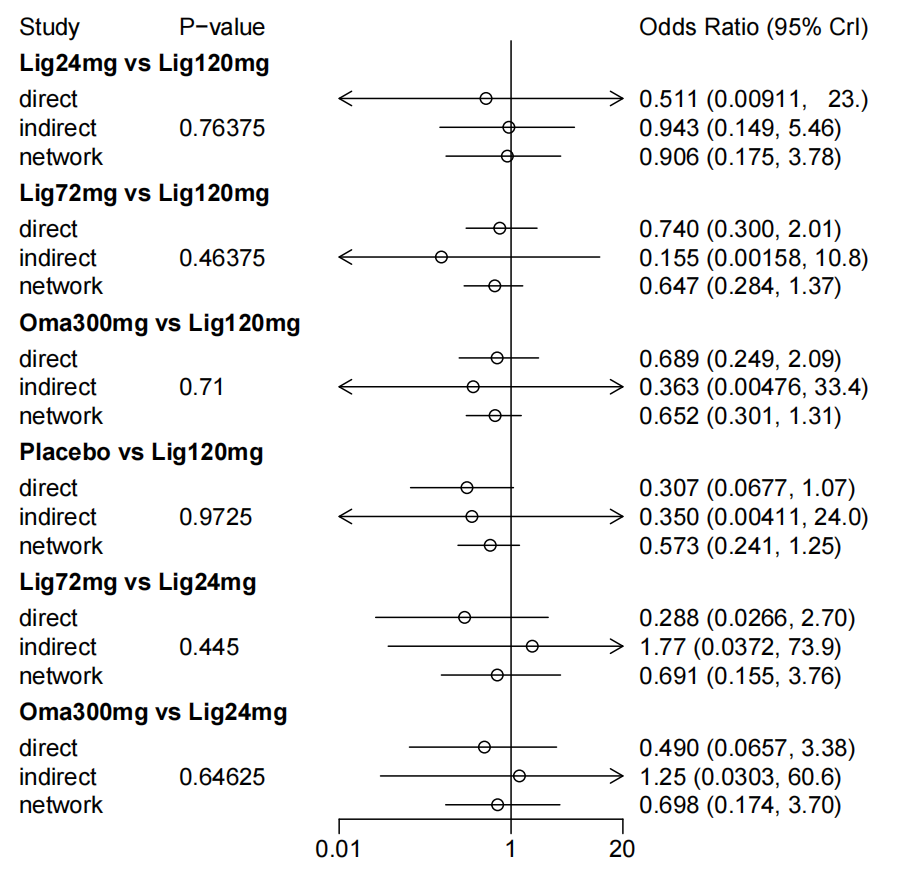


**eFigure 20.** Comparison-adjusted funnel plot

1. Change in weekly urticaria activity score (UAS7)


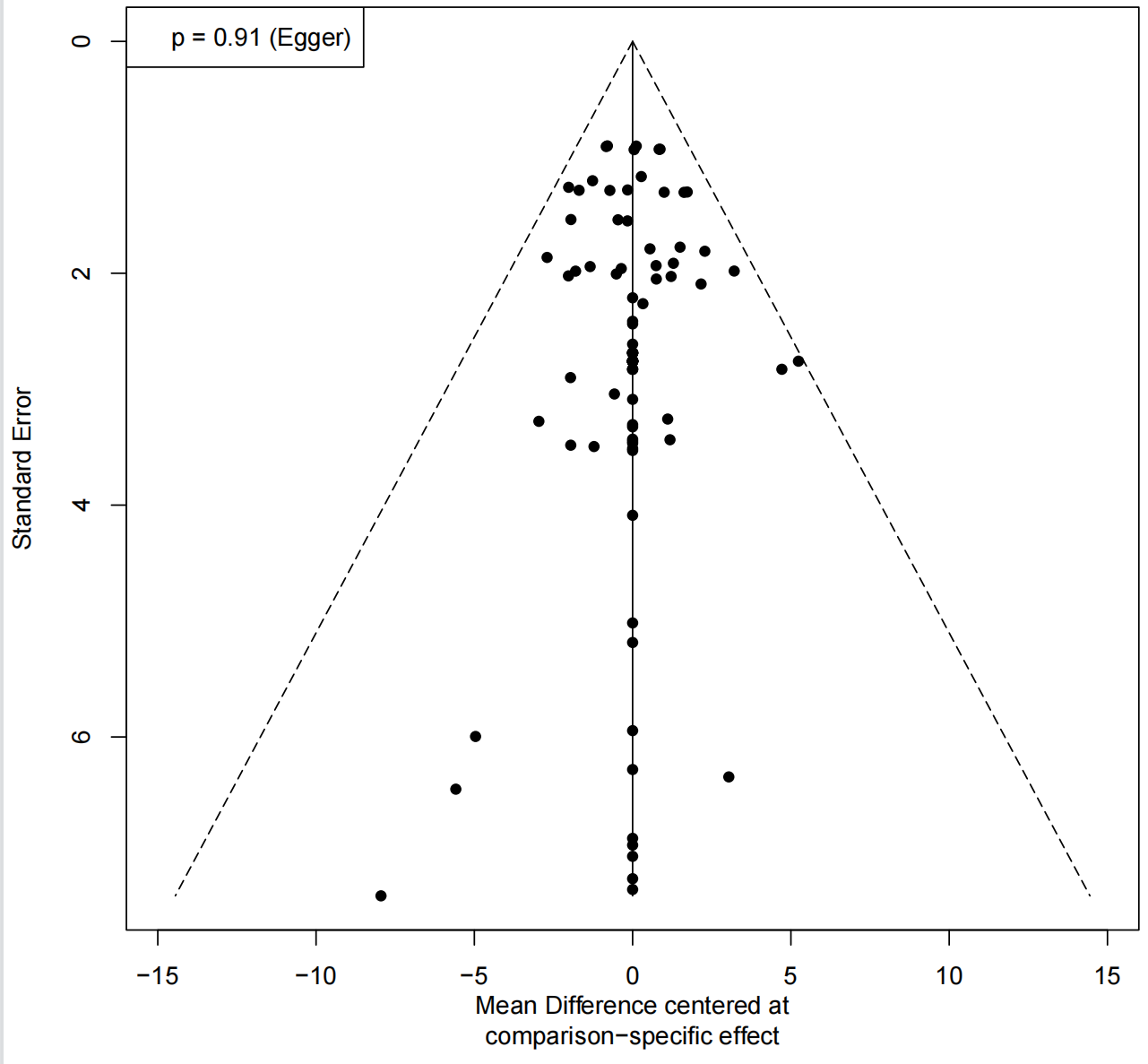


1. Proportion of patients achieved well-controlled disease (UAS7≤6)


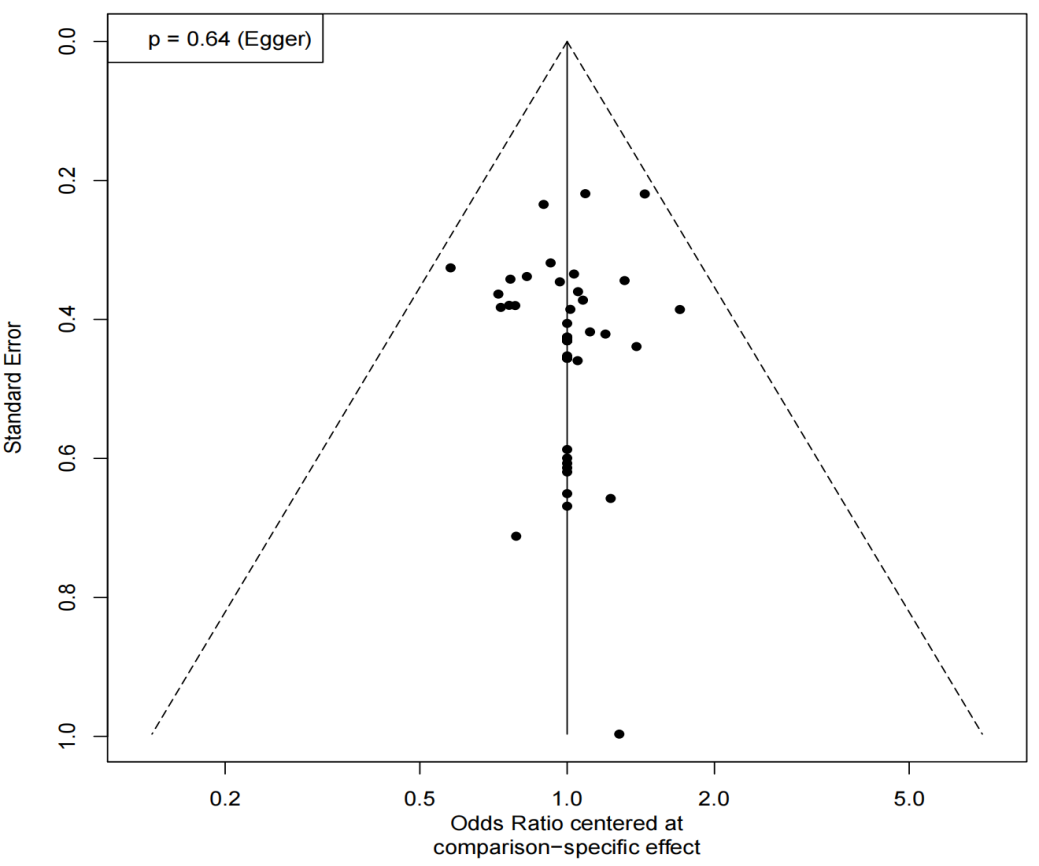


1. Proportion of patients achieved complete response to treatment (UAS7=0)


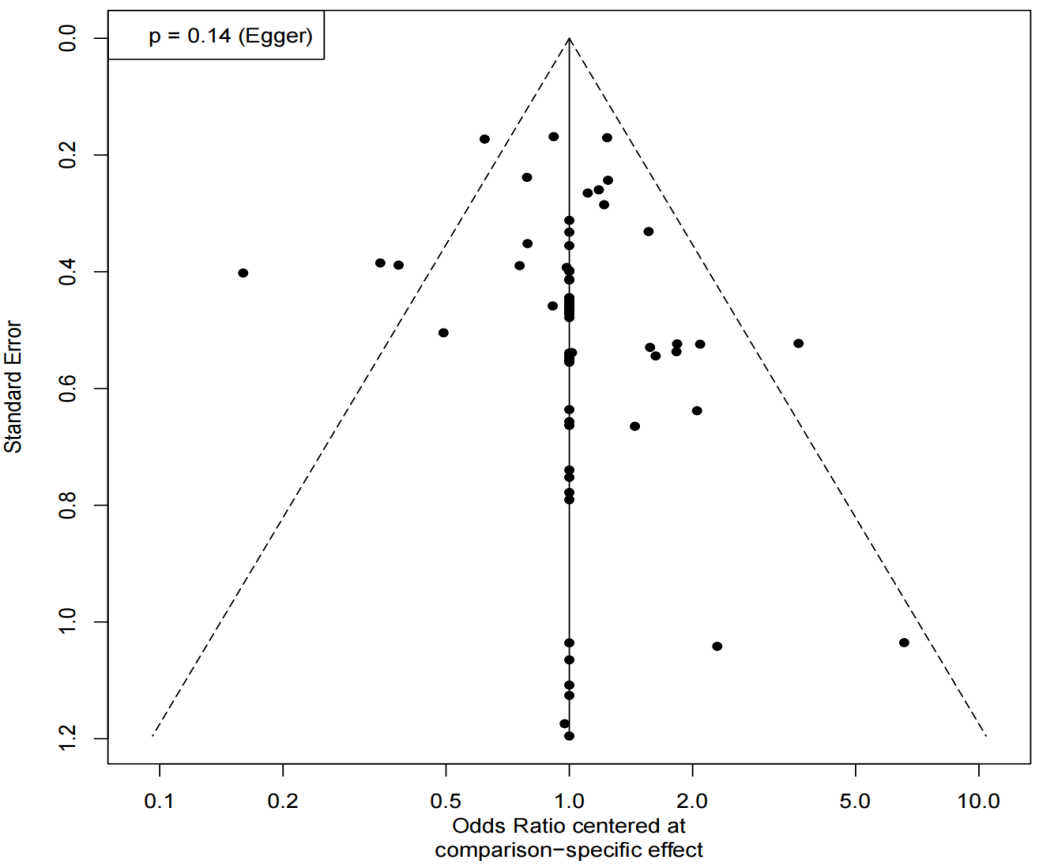


1. Weekly itch severity score (ISS7)


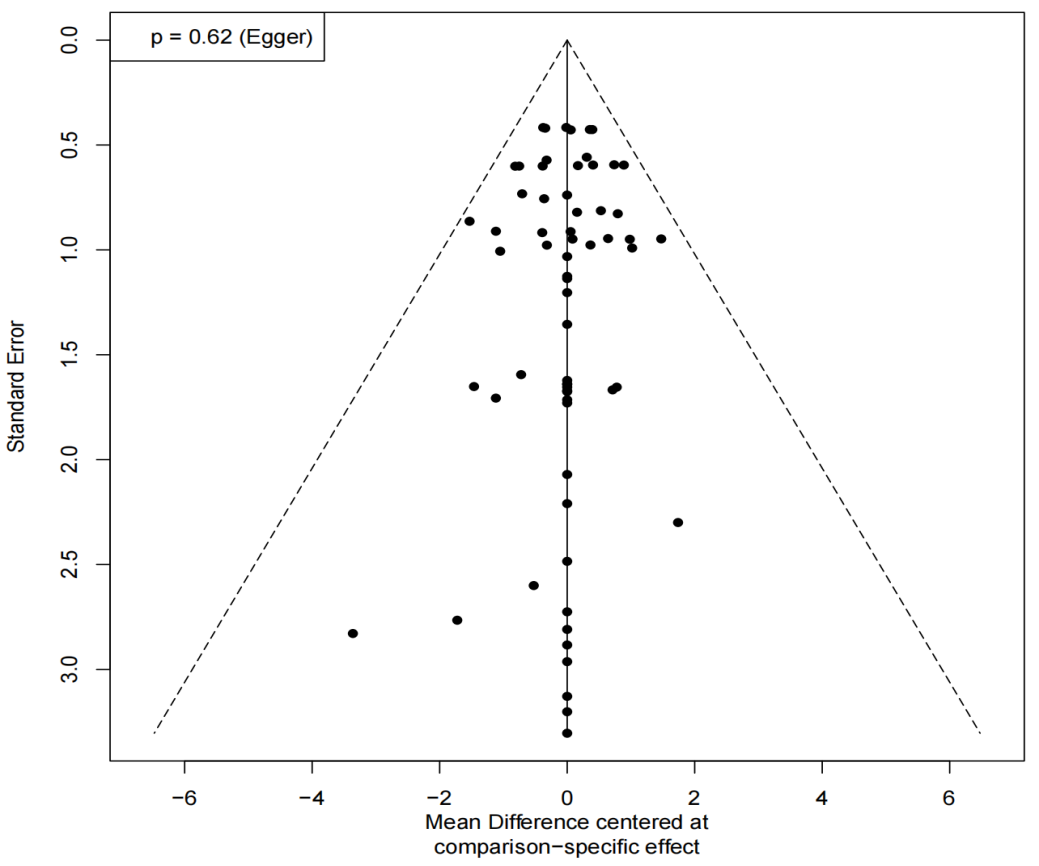


1. Weekly hive severity score (HSS7)


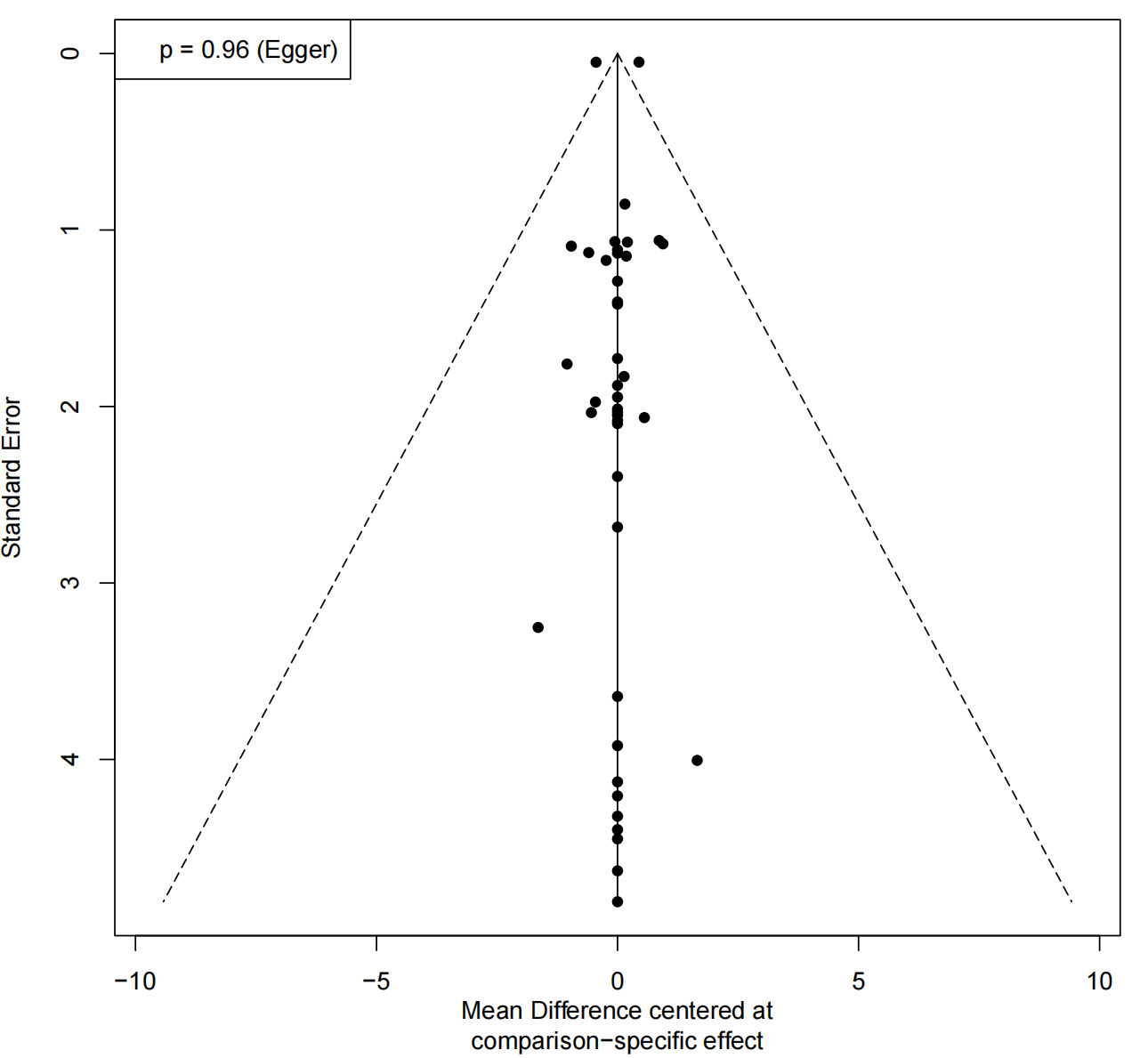


1. Dermatology life quality index (DLQI)


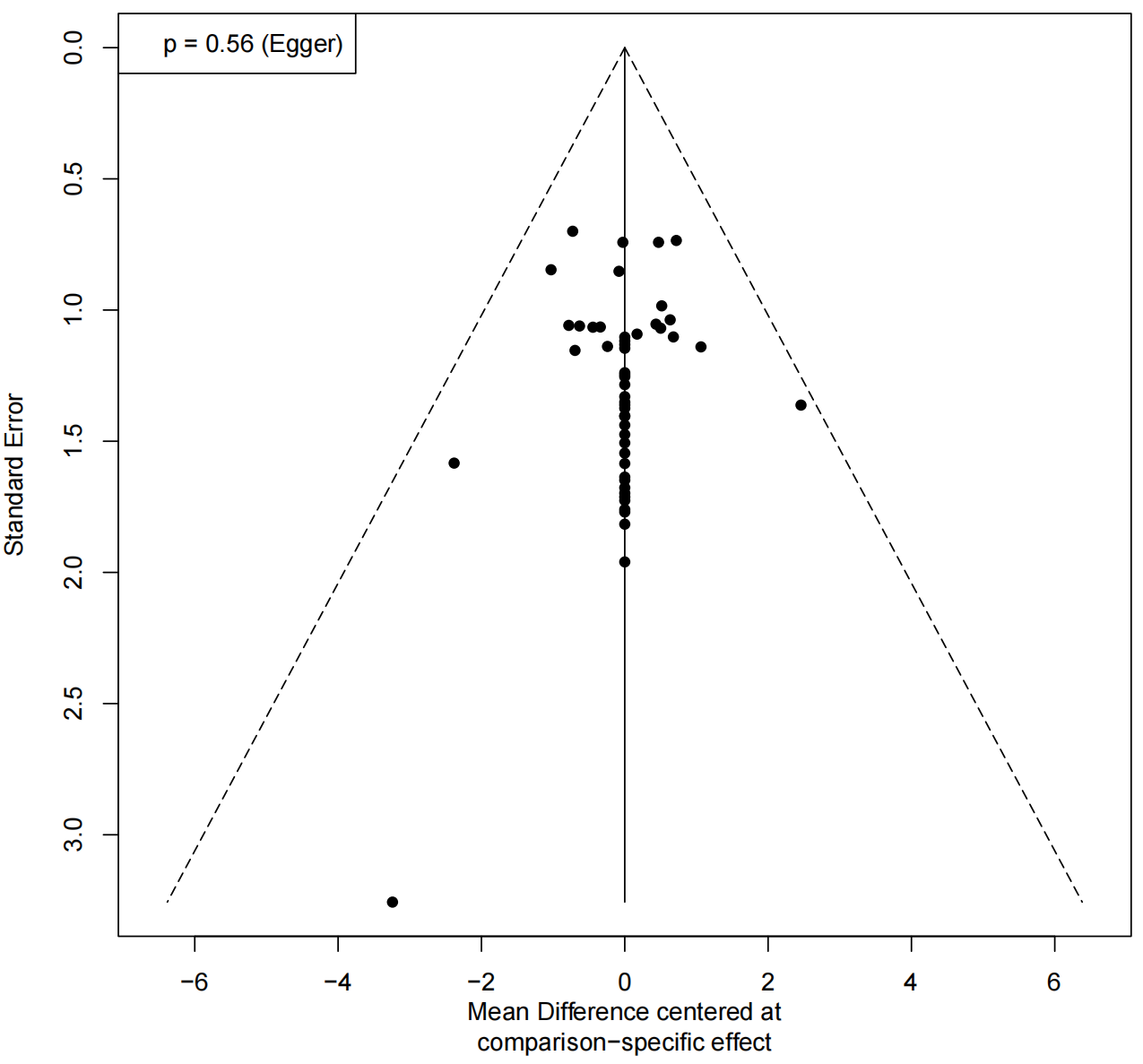


1. Adverse events (AEs): patient with one at least adverse event


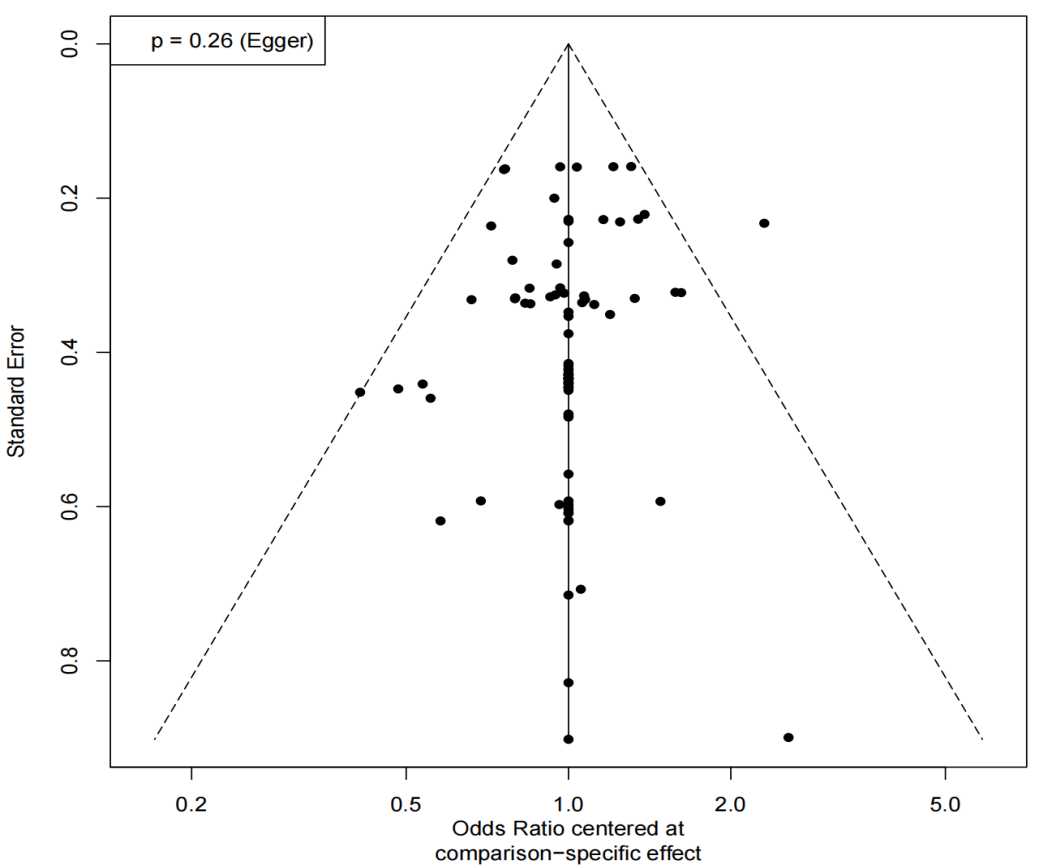


1. Serious adverse events (SAEs): patient with one at least adverse serious event


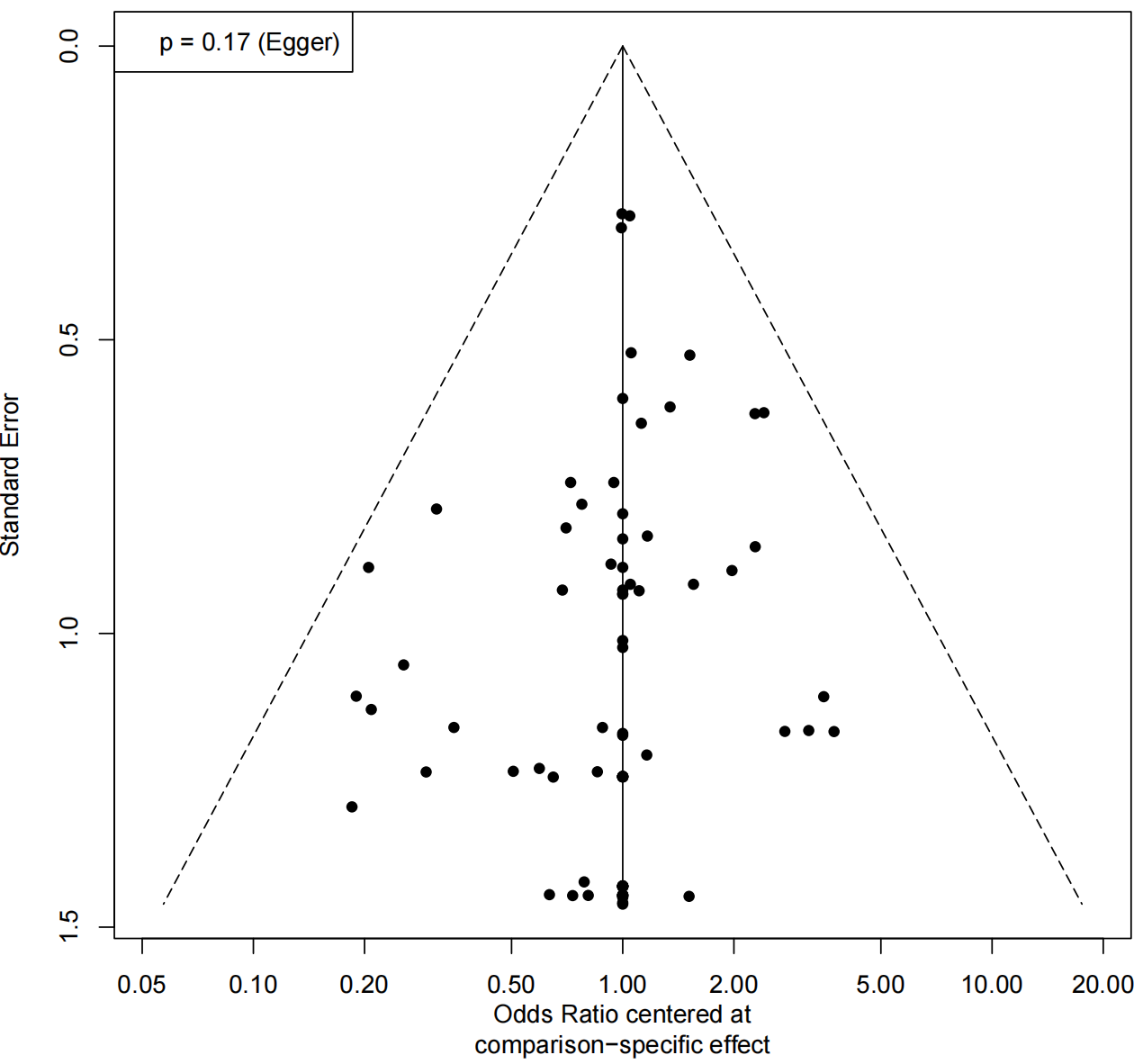

Supplement: Supplementary file 1 — Supporting Information S1 [file CLT2-15-e70052-s001.docx]
